# How cycloalkane fusion enhances the cycloaddition reactivity of dibenzocyclooctynes†‡

**DOI:** 10.1039/d3sc05789e

**Published:** 2024-01-08

**Authors:** Dennis Svatunek, Anton Murnauer, Zhuoting Tan, K. N. Houk, Kathrin Lang

**Affiliations:** a Department of Chemistry and Biochemistry, University of California Los Angeles California 90095-1569 USA; b Institute of Applied Synthetic Chemistry, TU Wien Getreidemarkt 9 1060 Vienna Austria dennis.svatunek@tuwien.ac.at; c Department of Chemistry and Applied Biosciences, ETH Zurich 8093 Zurich Switzerland kathrin.lang@org.chem.ethz.ch

## Abstract

Dibenzoannulated cyclooctynes have emerged as valuable compounds for bioorthogonal reactions. They are commonly used in combination with azides in strain-promoted 1,3-dipolar cycloadditions. They are typically, however, unreactive towards 3,6-disubstituted tetrazines in inverse electron-demand Diels–Alder cycloadditions. Recently a dibenzoannulated bicyclo[6.1.0]nonyne derivative (DMBO) with a cyclopropane fused to the cyclooctyne core was described, which showed surprising reactivity towards tetrazines. To elucidate the unusual reactivity of DMBO, we performed density functional theory calculations and revealed that a tub-like structure in the transition state results in a much lower activation barrier than in the absence of cyclopropane fusion. The same transition state geometry is found for different cycloalkanes fused to the cyclooctyne core albeit higher activation barriers are observed for increased ring sizes. This conformation is energetically unfavored for previously known dibenzoannulated cyclooctynes and allows tetrazines and azides to approach DMBO from the face rather than the edge, a trajectory that was hitherto not observed for this class of activated dieno- and dipolarophiles.

## Abstract 2

*Cis*-fusion of cyclopropane to dibenzocyclooctyne (DMBO) results in high cycloaddition reactivity *via* enforcing a unique tub-like structure in the transition state that allows tetrazines and azides to approach DMBO from an unhindered face trajectory.

## Graphical abstract

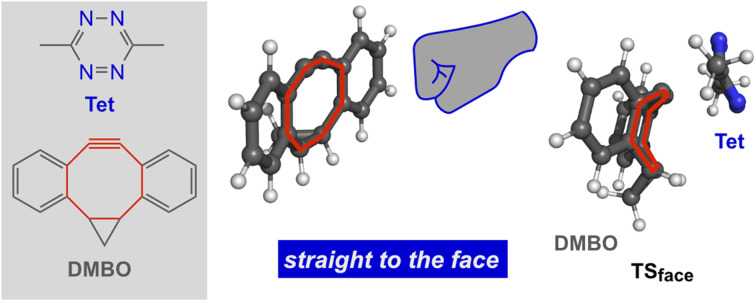

## Introduction

**Fig. 1:**
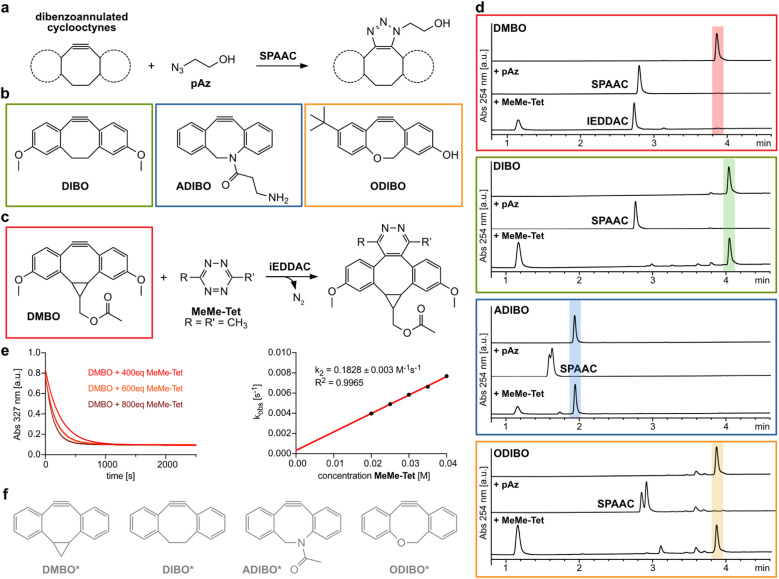
Dibenzoannulated cyclooctynes in SPAAC and iEDDAC reactions. (a) Dibenzoannulated cyclooctynes engage in SPAAC reactions with azides. (b) Structures of dibenzocyclooctyne derivatives DIBO, ADIBO and ODIBO used in this study. (c) DMBO engages in iEDDAC with 3,6-disubstituted 1,2,4,5-tetrazines such as MeMe-Tet. (d) Liquid-chromatography mass spectrometry (LC-MS) analysis reveals that only DMBO shows iEDDAC reactivity towards MeMe-Tet, while DIBO as well as ADIBO and ODIBO do not result in any product formation. In contrast, all four dibenzoannulated cyclooctynes DMBO, DIBO, ADIBO and ODIBO react quantitatively with pAz in SPAAC; reaction conditions: 250 μM cyclooctyne, 2.5 mM pAz or MeMe-Tet, room temperature in methanol, 40 hours. Complete data set can be found in ESI Fig. S1–S4 and S7–S10.‡ (e) Determination of second order rate constant between DMBO and MeMe-Tet. (Left) Exponential decay in DMBO absorbance at 327 nm in the presence of 400–800-fold excess of MeMe-Tet over time. Data were recorded at 25 °C in MeOH/PBS (1/1, v/v). By fitting the data to a single exponential equation, *k*_obs_ values were determined. (Right) *k*_obs_ values for different concentrations were plotted against concentration of MeMe-Tet and subjected to a linear fit; the slope of the plot yields the rate constant *k*_2_. Complete data sets are shown in ESI, ESI Fig. S5 and S6.‡ (f) Model structures of dibenzoannulated cyclooctynes used in DFT calculations.

Cyclooctynes are important reactants in bioorthogonal chemistry. They engage with azides in strain-promoted azide–alkyne cycloadditions (SPAAC) to form stable triazoles, establishing a versatile copper-free version of the popular click reaction (Fig. 1a).^1–5^ Simple aliphatic cyclooctynes display however rather slow reaction rates in SPAAC, compromising their utility for applications in biomolecule labeling.^4,6,7^ More reactive dibenzoannulated cyclooctynes such as DIBO (Fig. 1b)^8^ and ADIBO (Fig. 1b)^9^ were introduced by the groups of Boons and van Delft. These dibenzocyclooctynes show enhanced SPAAC reactivity due to the distortion of the alkyne carbons towards the transition state geometry resulting in lowered energies of distortion to achieve these transition states.^4,10^ One of the most reactive dibenzocyclooctynes – ODIBO (Fig. 1b) – was introduced by Popik *et al.* in 2012 and surpasses the previous cyclooctynes in terms of SPAAC-reactivity and stability.^11^ Numerous dibenzocyclooctyne-based bioorthogonal probes are nowadays commercially available and are frequently used for labeling azide-modified biomolecules. One notable exception to the rule that dibenzoannulation is a necessity to achieve acceptable SPAAC reactivity is BCN, bicyclo[6.1.0]non-4-yne. BCN was first introduced as a dienophile by Meier and coworkers,^12^ and later used by van Delft and coworkers for SPAAC.^13^ Here, reactivity is induced by *cis*-ring fusion of a cyclopropane to cyclooctyne. BCN is special in a further regard, as it is the only cyclooctyne that reacts also with 3,6-disubstituted 1,2,4,5-tetrazines in inverse electron-demand Diels–Alder cycloadditions (iEDDAC) with appreciable kinetic rates.^14–16^ The SPAAC-reactive sterically demanding dibenzoannulated cyclooctynes show typically extremely sluggish kinetics towards tetrazines.^17,18^ Houk *et al.* identified the steric demand of the benzo groups as the origin of this low reactivity.^10^ In particular, the *ortho*-hydrogen atoms of the aromatic rings of the dibenzocyclooctynes interfere sterically with the tetrazine substituents. While azide cycloadditions are generally not affected by the steric demand of the benzo groups, lower reactivity of tertiary azides with ADIBO were reported.^19^

By contrast, one of our groups showed that a cyclopropane-fused dibenzocyclooctyne (DMBO, Fig. 1c) reacts rapidly with 3,6-disubstituted tetrazines with reported on-protein rates of up to 50 M^−1^ s^−1^ in aqueous buffers.^20^ This high reactivity of DMBO towards tetrazines is surprising, given the established model that tetrazines are sterically hindered from reacting with the triple bond in dibenzocyclooctyne derivatives.

We have now investigated how *cis*-fusion of cyclopropane and larger cycloalkanes to the dibenzocyclooctyne core in DMBO increases their reactivity towards 3,6-disubstituted tetrazines and sterically-demanding azides.

## Results and discussion

First, we investigated the reactivity of tetrazines with dibenzocyclooctynes. Experimentally, incubation of DMBO (250 μM) with a 10-fold excess of 3,6-dimethyl-1,2,4,5-tetrazine (MeMe-Tet, Fig. 1d and S1‡) led to quantitative conversion to the corresponding iEDDAC product, while incubation of DIBO and MeMe-Tet under the same reaction conditions did not result in any pyridazine formation even after 40 hours (Fig. 1d and S2‡). By following the exponential decay of the characteristic DMBO absorption at 327 nm, we determined a second order rate constant of 0.18 M^−1^ s^−1^ for the reaction of DMBO with MeMe-Tet under pseudo-first order conditions in MeOH/PBS (1/1, v/v) at 25 °C (Fig. 1e, S5 and S6‡). On-protein and in aqueous buffer a second order rate constant of *ca.* 50 M^−1^ s^−1^ had been reported between DMBO and a protein bearing a more reactive tetrazine (3-(hydroxymethylphenyl)-6-methyl-1,2,4,5-tetrazine),^20^ attesting to the fact that Diels–Alder cycloadditions of 1,2,4,5-tetrazines proceed faster in water than in organic solvents.^21–24^

Our computational investigations of the unusual reactivity of DMBO with tetrazines employed MeMe-Tet and a model DMBO structure (DMBO*, Fig. 1f) and compared their reaction to that between a model DIBO (DIBO*, Fig. 1f) and MeMe-Tet.

Density functional theory (DFT) has been successfully used to unravel the origin of reactivity and selectivity in cycloaddition reactions and in particular bioorthogonal cycloadditions.^19,25–30^

DFT calculations were performed using Gaussian 16. We used the M06-2X-D3 functional^31–33^ in combination with the 6-311+G(d,p)^34^ basis set and the SMD solvent model^35^ (for details see ESI‡).

To gain mechanistic insight, we also employed distortion/interaction analysis (DIA) combined with an energy decomposition analysis (EDA) using the ADF software package.^36,37^ In the distortion/interaction analysis, the electronic energy along a reaction coordinate is dissected into two contributing energy terms, the distortion energy, Δ*E*_dist_, and the interaction energy, Δ*E*_int_. Δ*E*_dist_ is the energy needed to distort the reactants. Δ*E*_int_ is the interaction energy when the distorted reactants are brought together. The interaction energy can further be decomposed using EDA. Δ*E*_OI_ accounts for orbital interactions between filled and empty orbitals. Δ*V*_elstat_ describes electrostatic interactions between the reactants. The Pauli repulsion term, Δ*E*_Pauli_, accounts for destabilizing interactions between filled orbitals of the reactants and is responsible for steric repulsion.

**Fig. 2:**
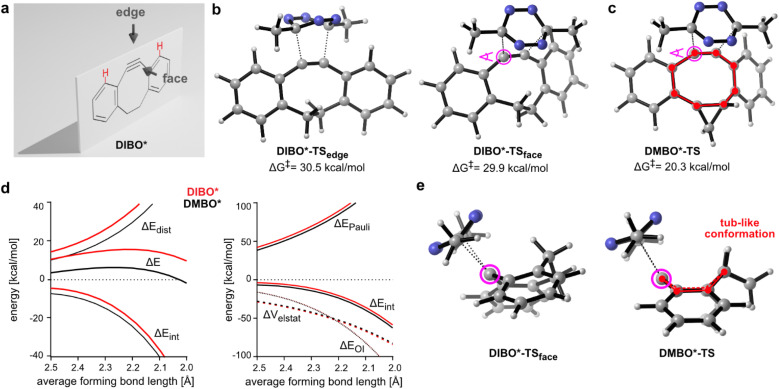
DFT calculations for TSs of DIBO* and DMBO* in iEDDAC reaction with MeMe-Tet. (a) Schematic representation of the face and edge approach for dibenzoannulated cyclooctynes. (b) Transition state geometries and Gibbs free energy barriers for the face and edge approach of MeMe-Tet on DIBO* calculated using M06-2X-D3/6-311+G(d,p) SMD(water). Forming bond lengths are given in angstrom. (c) Transition state geometry and Gibbs free energy barrier for the reaction of MeMe-Tet and DMBO* calculated using M06-2X-D3/6-311+G(d,p) SMD(water). Forming bond lengths are given in angstrom. (d) Distortion/interaction analysis (DIA; left side) and energy decomposition analysis (EDA; right side) along the intrinsic reaction coordinate for the reaction of MeMe-Tet with DIBO* or DMBO*. The analysis was performed in ADF using M06-2X-D3/TZ2P on M06-2X-D3/6-311+G(d,p) SMD(water) calculated geometries. (e) View along the dibenzocyclooctyne triple bond for the face transition state of DIBO* with MeMe-Tet and DMBO* with MeMe-Tet as indicated in purple in (b) and (c). Tub-like conformation of the DMBO*-TS is highlighted. An interactive website for 3D visualization of modelled compounds can be accessed at: https://dsvatunek.github.io/cycloalkane_fused_dibenzocyclooctynes/.

There are two different trajectories a tetrazine can follow when reacting with the triple bond of dibenzocyclooctynes leading to the same product. The tetrazine can either approach the plane of the cyclooctyne from the face or from the edge (Fig. 2a). Previously, for dibenzoannulated cyclooctynes such as DIBO only the approach at the edge was considered, and steric repulsion with the *ortho*-hydrogen atoms on the benzo moieties was identified as the reason for the low reactivity.^10^ Our calculations show an almost identical activation barrier of approx. 30 kcal mol^−1^ for the reaction between MeMe-Tet and DIBO* for both the face and edge transition states (Fig. 2b). While in the edge case the high barrier is associated with the previously reported steric repulsion and an accompanying high distortion, for the face approach the steric repulsion and distortion are lowered. The overall interaction is however decreased due to lowered orbital interaction (ESI Tables S1 and S2‡).

For the cyclopropane-fused DMBO*, only one feasible trajectory is found. Attack of MeMe-Tet from the face of the DMBO* plane lowers the free energy barrier by almost 10 kcal mol^−1^ to 20.3 kcal mol^−1^ (Fig. 2c). A comparable activation barrier of 20.4 kcal mol^−1^ was also calculated when taking the influence of the methoxy groups into account (ESI Fig. S11‡), establishing DMBO* as suitable and valid model compound and verifying that electronic effects play a subordinate role. The calculated activation energy of 20.3 kcal mol^−1^ is in reasonable agreement with our determined experimental free energy of activation of 18.4 kcal mol^−1^. DIA and EDA for reactions of DIBO* and DMBO* with MeMe-Tet show that along the whole reaction coordinate both the distortion and interaction energy favour DMBO* (Fig. 2d). Further analysis of Δ*E*_int_ shows that the favourable interaction energy is rooted in a lowered Pauli repulsion. Orbital interaction strength (Δ*E*_OI_) and electrostatic interactions (Δ*V*_elstat_) are almost identical for DIBO* and DMBO* along the reaction coordinate (ESI Table S1‡). An additional EDA/DIA analysis, which takes the asynchronicity of the TS between DIBO* and MeMe-Tet into account, shows the same trend as using average bond forming lengths (ESI Table S3‡). When looking along the triple bond of the DIBO*- and DMBO*-transition states (TSs), the highly twisted nature of DIBO*-TS_face_ becomes apparent, while DMBO*-TS adopts a tub-like conformation due to the *cis*-fused cyclopropane ring (Fig. 2e). The cyclopropane in DMBO* thus significantly reduces the distortion energy of the dibenzocyclooctyne, which accounts for most of the reduction in activation energy. Additionally, the tub-like conformer allows the benzo groups to be pointed away from the tetrazine, reducing Pauli repulsion. Importantly, identical tub-like transition state geometries were also obtained when including the electronic properties of the methoxy substituents into our calculations (ESI Fig. S11‡). A similar concept of conformational acceleration of strained dienophiles has been identified by Fox and coworkers in the case of *trans*-cyclooctene/tetrazine cycloadditions.^21,38^

**Fig. 3:**
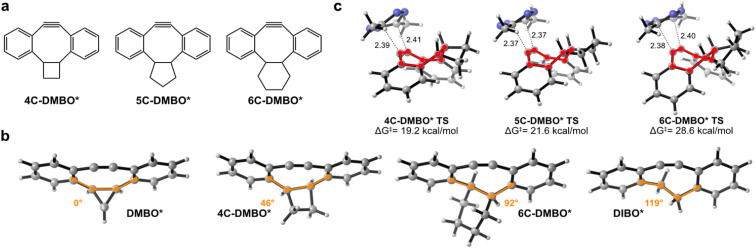
Influence of different cycloalkane-fusions on structure and reactivity of dibenzoannulated cyclooctynes. (a) Structures of hypothesized cyclobutane- (4C-DMBO*), cyclopentane- (5C-DMBO*) and cyclohexane- (6C-DMBO*) fused dibenzocyclooctynes used for DFT calculations. (b) Ground state geometries of DMBO*, 4C-DMBO*, 6C-DMBO* and DIBO* obtained by DFT calculations using M06-2X-D3/6-311+G(d,p) SMD(water). The dihedral angle of the C-4 backbone unit is highlighted in orange and absolute deviation from a planar 0° angle is given. (c) Transitions state geometries and activation barriers for the reaction between MeMe-Tet and 4C-DMBO*, 5C-DMBO* or 6C-DMBO* obtained by DFT calculations using M06-2X-D3/6-311+G(d,p) SMD(water). Face approach and tub-like structure (highlighted in red) is observed for all cycloalkane-fused dibenzocyclooctynes. Forming bond lengths are given in angstrom. An interactive website for 3D visualization of modelled compounds can be accessed at: https://dsvatunek.github.io/cycloalkane_fused_dibenzocyclooctynes/.

The intriguing reactivity observed between DMBO and MeMe-Tet prompted us to investigate if larger *cis*-fused cycloalkanes would exhibit similar effects as the cyclopropane in DMBO. To explore this, we evaluated the reaction between MeMe-Tet and hypothesized derivatives with cyclobutane, cyclopentane, or cyclohexane fused to the dibenzocyclooctyne core, which we named 4C-DMBO*, 5C-DMBO*, and 6C-DMBO* (Fig. 3a and S12a‡). We first compared their ground state geometries. While DMBO* shows a near planar 8-membered ring, showcasing an enforced *cis* conformation at the bridgehead carbons along the sigma C-2 bond, the cyclooctyne ring becomes progressively less constrained, as we proceed from cyclopropane to cyclobutane, cyclopentane, and finally cyclohexane (Fig. 3b and S12b‡). Larger rings provide thereby increasing flexibility to the C-2 sigma bond. In fact, 6C-DMBO* exhibits an s-*trans* configuration along the C2-sigma bond, mirroring the configuration observed in DIBO* (Fig. 3b and S12b‡). Notably, for the attack of MeMe-Tet on these cyclooctyne derivatives, we identified exclusively face approaches for all of them (Fig. 3c and S13‡). Activation barriers are increasing with fused cycloalkane size from 19.2 to 21.6 and 28.6 kcal mol^−1^ for 4C-DMBO* to 5C-DMBO* and 6C-DMBO*, respectively. 4C-DMBO* shows even slightly higher reactivity than cyclopropane-fused DMBO* in the reaction with MeMe-Tet, attesting that enhanced iEDDAC reactivity is not exclusive to cyclopropane fusion. This increased reactivity, stemming from a reduced distortion energy and increased orbital interaction, indicates that this ring size might offer a suitable balance between rigidity and flexibility (ESI Tables S3 and S4‡). 5C-DMBO* presents a slightly reduced reactivity, with an activation barrier 1.3 kcal mol^−1^ higher than DMBO*. Interestingly, the 6-membered ring derivative, 6C-DMBO*, offers only a marginal improvement of 1.3 kcal mol^−1^ over the unmodified DIBO* for face approach of MeMe-Tet. A closer examination of the transition state structures revealed a tub-like conformation in all three derivatives (Fig. 3c and S13‡). The energy necessary to achieve this tub-like transition state rises in tandem with the reduced confinement and strain of the cyclooctynes as demonstrated by DIA/EDA analysis (ESI Table S4‡), highlighting the potential of *cis*-cycloalkane fusions to the cyclooctyne core. Specifically, small rings such as cyclopropanes and cyclobutanes, which robustly enforce a highly planar conformation of the cyclooctyne in their ground state geometry and thereby a s-*cis* configuration at the C-2 unit, have the ability to greatly expedite reactivity of dibenzocyclooctynes when reacting with tetrazines in iEDDAC.

**Fig. 4:**
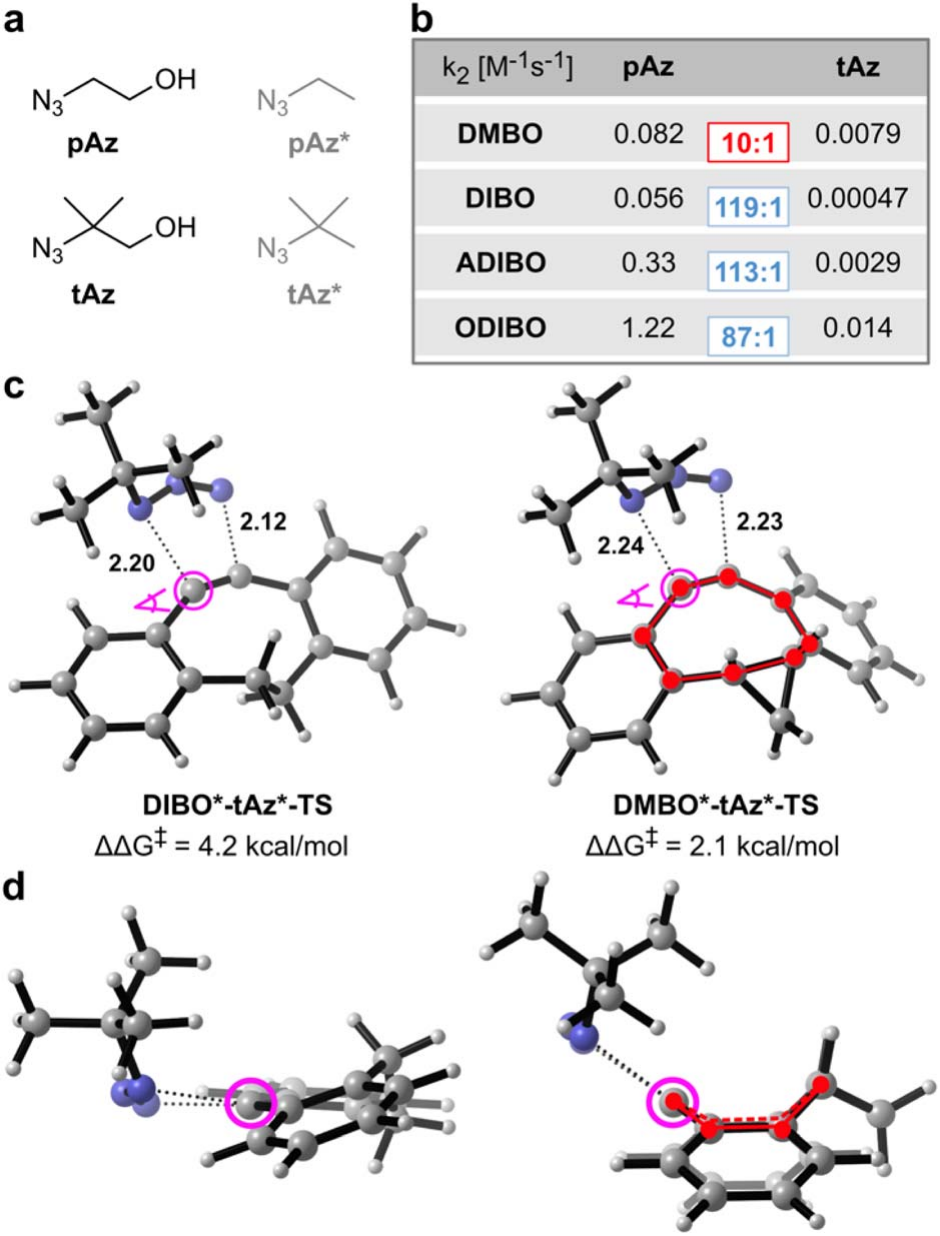
Dibenzoannulated cyclooctynes in SPAAC reactions with primary and tertiary azides. (a) Structures of primary and tertiary azides used experimentally (pAz and tAz) and model structures used for DFT calculations (pAz* and tAz*). (b) Second-order rate constants for all four cyclooctynes were determined under pseudo-first order conditions with 50–3000-fold excess of pAz and tAz at 25 °C in pure methanol. Ratios between *k*_2_ with pAz and tAz are shown in colored rectangles. Original kinetics data are shown in ESI Fig. S14–S21.‡ (c) Transition state geometries of tAz* with DIBO* and DMBO* using M06-2X-D3/6-311+G(d,p) SMD(methanol). Forming bond lengths are given in angstrom. ΔΔ*G*^‡^ indicates the difference between the Gibbs free energy barrier of tAz* and pAz* with the given cyclooctyne. Gibbs free energy barriers are listed in ESI Table S3.‡ (d) Transition state geometry for DIBO*-tAz*-TS and DMBO*-tAz*-TS with view along the dibenzocyclooctyne triple bond as indicated in (c). Tub-like conformation in the DMBO*-tAz*-TS is highlighted.

Given the observed reduced steric interactions of DMBO in reactions with tetrazines, we were curious if this effect can also be observed with sterically demanding azides (Fig. 4a). We synthesized four different dibenzocyclooctynes (DIBO, ADIBO, ODIBO, and DMBO, Fig. 1b and c, ESI‡) some of which had been reported to be very reactive SPAAC partners. While only DMBO was reactive towards MeMe-Tet, and no pyridazine product was observed when reacting 250 μM DIBO, ADIBO, and ODIBO with 2.5 mM MeMe-Tet for 40 hours, all four cyclooctynes reacted readily with azides (Fig. 1d, S1–S4 and S7–S10‡). We measured second order rate constants of all four cyclooctynes with a primary azide (pAz) and tertiary azide (tAz) under pseudo-first order conditions by following the exponential decay in characteristic cyclooctyne absorption between 309 and 327 nm in methanol (Fig. 4b and S14–S21‡). As expected, for ODIBO, ADIBO, and DIBO the primary azide is favoured by a reactivity difference of 90 to 120-fold compared to the sterically more demanding tertiary azide. However, in case of DMBO this difference is lowered to only 10-fold, suggesting that a similar effect as with tetrazines is at play. We calculated energy barriers of SPAAC reactions for model cyclooctynes DIBO*, ADIBO*, ODIBO* and DMBO* (Fig. 1f), and model azides pAz* and tAz* (Fig. 4a). In qualitative agreement with the experimental results, the difference in Gibbs free energy of activation between tertiary and primary azide, ΔΔ*G*^‡^, ranges from 3.8 to 4.2 kcal mol^−1^ for DIBO*, ADIBO*, and ODIBO* while it is reduced to only 2.1 kcal mol^−1^ for DMBO* (ESI Tables S5 and S6‡). The calculated difference in activation barrier suggests a 30-fold faster reaction for DMBO* with pAz* over tAz*. This value is in qualitatively good agreement with the 10-fold difference determined in our kinetic studies. For model compounds DIBO*, ADIBO*, and ODIBO* the calculated energy barriers of 3.8 to 4.2 kcal mol^−1^ suggest an approx. 1000-fold faster reaction with pAz*, systematically overestimating the experimentally determined *ca.* 100-fold acceleration by roughly one order of magnitude (1.4 kcal mol^−1^). Nonetheless, despite this small systematic error the calculations reflect the trend observed in the kinetic studies, testifying that DMBO better alleviates steric restraints in SPAAC reactions with bulky azides than previously reported cyclooctynes.^19^ To understand the origin of difference in reaction of tAz and pAz with dibenzocyclooctynes, we compared the transition states of tAz* with DIBO* and DMBO* (Fig. 4c and d). In the transition state for the reaction between DIBO* and tAz*, the dibenzocyclooctyne adopts a geometry that leads to steric repulsion between the tertiary alkyl group of tAz* and one of the benzo groups as described before for ADIBO*.^19^ The shorter intermolecular distance between the *ortho*-benzo hydrogen of DIBO* and one of the *tert*-butyl hydrogens is only 2.45 Å (ESI Fig. S22‡). Additionally, this causes a more asynchronous reaction. For DMBO*, once again the tub-like conformation is adapted, which twists the benzo groups away from the sterically demanding alkyl group, leading to less repulsion and a more synchronous reaction. The shortest distance between a *tert*-butyl hydrogen and the *ortho*-benzo hydrogen of DMBO* is 2.73 Å. An additional DIA/EDA analysis on the SPAAC reaction of DMBO* and DIBO* shows that the tub-like transition state geometry adopted by DMBO* reduces the Pauli repulsion during the reaction (ESI Table S7‡).

Despite the ability of DMBO to lower steric repulsion with cycloaddition reaction partners, the tertiary azide still reacts one order of magnitude slower than the equivalent primary azide pAz. Analysis of the transition state geometry of DMBO* and pAz* combined with EDA reveals that a lower Pauli repulsion and distortion energy in case of pAz* is cause of this difference, confirming the increased steric hindrance in case of tAz* (ESI Fig. S23 and Table S8‡).

## Conclusions

In conclusion we have shown that the unusual reactivity of DMBO towards tetrazines is based on the conformation enforced by the *cis*-fused cyclopropane. DFT calculations have determined a tub-like structure of DMBO in the transition state allowing tetrazines to approach the strained alkyne from the face side resulting in a *ca.* 10 kcal mol^−1^ lowered activation barrier with MeMe-Tet compared to dibenzocyclooctynes lacking a cyclopropane fusion. DIA and EDA showed that a reduction in distortion energy and Pauli repulsion between DMBO and MeMe-Tet caused by a tub-like transition state geometry of the cyclooctyne are the main contributors for the unusual reactivity of DMBO towards tetrazines.

Expanding on this, the investigations into larger *cis*-fused cycloalkanes like cyclobutane, cyclopentane, and cyclohexane further deepen our understanding. Our calculations reveal a discernible dependence on ring size. As the fused cycloalkane ring increases in size, the constrained conformation enforced by the *cis*-fusion becomes less stringent, leading to varied iEDDAC reactivity with tetrazines. Specifically, while the cyclobutane derivative mirrors the reactivity observed for DMBO*, the cyclopentane and cyclohexane derivatives display progressively diminished reactivity, with the latter showing only a marginally reduced activation energy compared to DIBO*.

Interestingly, the novel face-side attack onto dibenzoannulated cyclooctynes is also transferable to SPAAC reactions. In DFT calculations the difference in activation barrier between sterically more demanding tertiary azides and the corresponding primary azides is much smaller for DMBO* compared to literature known dibenzoannulated cyclooctynes like DIBO*, ADIBO*, and ODIBO* (ΔΔ*G*^‡^ of 2.1 kcal mol^−1^*vs.* 3.8–4.2 kcal mol^−1^). These computational results are nicely confirmed by kinetic studies, where only a tenfold difference in *k*_2_ between pAz and tAz was determined for DMBO, while DIBO, ADIBO, and ODIBO reacted two orders of magnitude faster with pAz compared to tAz.

Our results reveal a previously unrecognized factor influencing the rates of reactions of strained alkynes, allowing to explain and predict their selectivity towards different bioorthogonal groups and we disclose a new face trajectory for cycloaddition of dibenzoannulated cyclooctynes. This will facilitate design of novel cyclooctynes to expand the existing toolbox of bioorthogonal compounds and should also enable cyclooctyne reporters of contrasting reactivity for dual orthogonal bioorthogonal labeling.

## Data availability

The datasets supporting this article have been uploaded as part of the ESI.‡

## Author contributions

D. S. performed DFT calculations, the associated data evaluation, and designed the website for 3D structure visualization of modelled compounds. A. M. performed the synthetic organic work, LC-MS assays, kinetic experiments and the associated data evaluation. Z. T. helped with DFT calculations. The publication was written by D. S. and K. L. with inputs from A. M. and K. N. H. The work was supervised by K. N. H. and K. L.

## Conflicts of interest

There are no conflicts to declare.

## Supplementary Material

SC-015-D3SC05789E-s001

SC-015-D3SC05789E-s002
