# Supplementary material for: How cycloalkane fusion enhances the cycloaddition reactivity of dibenzocyclooctynes
Source: Chem Sci. 2024 Jan 8;15(6):2229–35. doi: 10.1039/d3sc05789e (PMC10848739; doi:10.1039/d3sc05789e)
Supplement: SC-015-D3SC05789E-s002 [file SC-015-D3SC05789E-s002.pdf]

Electronic Supplementary Material (ESI) for Chemical Science.
This journal is © The Royal Society of Chemistry 2024

Supporting Information

How Cycloalkane Fusion Enhances the Cycloaddition Reactivity of Dibenzocyclooctynes

Dennis Svatunek#*,[a,b] Anton Murnauer#,[c] Zhuoting Tan,[a] K. N. Houk[a] and Kathrin Lang*[c]

[a] Dr. Dennis Svatunek, Zhuoting Tan, Prof. Dr. K. N. Houk
Department of Chemistry and Biochemistry, University of California, Los Angeles, California 90095-1569, USA
[b] Dr. Dennis Svatunek
Institute of Applied Synthetic Chemistry, TU Wien, Getreidemarkt 9, 1060 Vienna, Austria
[c] Anton Murnauer, Prof. Dr. K. Lang
Laboratory of Organic Chemistry, Department of Chemistry and Applied Biosciences, ETH Zürich, Vladimir-Prelog-Weg 3, 8093 Zurich, Switzerland
Email: kathrin.lang@org.chem.ethz.ch

# These authors contributed equally.

* to whom correspondence should be addressed: kathrin.lang@org.chem.ethz.ch and dennis.svatunek@tuwien.ac.at

Abstract: Dibenzoannulated cyclooctynes have emerged as valuable compounds for bioorthogonal reactions. They are commonly used in combination with azides in strain-promoted 1,3-dipolar cycloadditions. They are typically, however, unreactive towards 3,6-disubstituted tetrazines in inverse electron-demand Diels-Alder cycloadditions. Recently a dibenzoannulated bicyclo[6.1.0]nonyne derivative (**DMBO**) with a cyclopropane fused to the cyclooctyne core was described, which showed surprising reactivity towards tetrazines. To elucidate the unusual reactivity of **DMBO**, we performed density functional theory calculations and revealed that a tub-like structure in the transition state results in a much lower activation barrier than in the absence of cycloproprane fusion. The same transition state geometry is found for different cycloalkanes fused to the cylclooctyne core albeit higher activation barriers are observed for increased ring sizes. This conformation is energetically unfavored for previously known dibenzoannulated cyclooctynes and allows tetrazines and azides to approach **DMBO** from the *face* rather than the *edge*, a trajectory that was hitherto not observed for this class of activated dieno- and dipolarophiles.

Table of Content

General ... 3
Synthesis ... 4
Synthesis of photo-DMBO ... 4
Synthesis of photo-DIBO ... 8
Synthesis of photo-ODIBO ... 9
Handling and storage of cyclopropenones ... 12
LC-MS Assays ... 13
Photo Deprotection ... 13
Synthesis of iEDDAC products ... 13
Synthesis of SPAAC products ... 13
Kinetics ... 15
UV-Vis characterization of cyclooctynes and reaction products ... 15
Kinetic Measurements and data evaluation ... 15
Computational Methods and Results ... 16
Discussion on the used DFT Method ... 16
Supplementary Tables S1-S8 ... 17
Supplementary Figures S1 – S23 ... 21
References ... 44
NMR Spectra ... 45

General

All commercially available chemicals and solvents (Sigma Aldrich, Acros, Carbolution, Alfa Aesar, TCI) were used without further purification unless otherwise stated. ADIBO was purchased from Sigma Aldrich. All air or moisture sensitive reactions were carried out under argon atmosphere using standard Schlenk techniques in oven-dried glassware, which was further dried under vacuum using a heat gun before usage. Flash column chromatography used for product purification was performed on silica gel 60 (230-400 mesh). Thin-layer chromatography (TLC) was performed on Merck Millipore silica gel 60 F-254 plates. The developed silica plates were visualized by UV light (254nm) and/or staining with potassium permanganate, ninhydrin or ceric ammonium molybdate (CAM) solutions, followed by heating.

NMR spectra were recorded on Bruker AVHD300 (300 MHz for ^{1}H-NMR, 75 MHz for ^{13}C- NMR), Bruker AVHD400 (400 MHz for ^{1}H-NMR, 101 MHz for ^{13}C-NMR) or Bruker AVANCE 500 UltraShield^{TM} spectrometer (500 MHz for ^{1}H-NMR, 125 MHz for ^{13}C-NMR). Chemical shifts (δ), reported in ppm, are referenced to the residual proton solvent signal. Coupling constants (J) are reported in Hertz (Hz) while peak multiplicities are descripted as follows: s (singlet), d (doublet), t (triplet), q (quartet), quint (quintet), m (multiplet), b (broad) or combinations thereof.

Small molecule LC-MS was carried out on an Agilent Technologies 1260 Infinity LC-MS system with a 6310 Quadrupole spectrometer. The solvent system consisted of 0.1 % formic acid in water as buffer A and 0.1 % formic acid in ACN as buffer B. Small molecule LC-MS was carried out on a Phenomenex Luna^{TM} Omega PS C18 (pore size 100 Å; 100 × 2.1 mm, particle size 3 µm). The samples were analysed in both positive and negative mode as well as UV absorbance at 193, 254 and/or 280 nm.

For photo-deprotection of the cyclopropenones a Vilber Lourmat VL-215.L UV lamp (2 x 15W 365 nm lamps) was used. UV-Vis spectra and kinetics were recorded on a Cary 3500 UV-Vis spectrophotometer by Agilent. The measurements were performed in capped Hellma Ultra-Micro quartz glass cuvettes with a volume of 160 µl and an optical path length of 10 mm.

All volumes containing methanol for assays or kinetics were transferred using Hamilton syringes with a total volume of 25, 100 or 500 µl.

Synthesis

Synthesis of photo-DMBO

Bis(3-methoxyphenyl)acetylene[1]

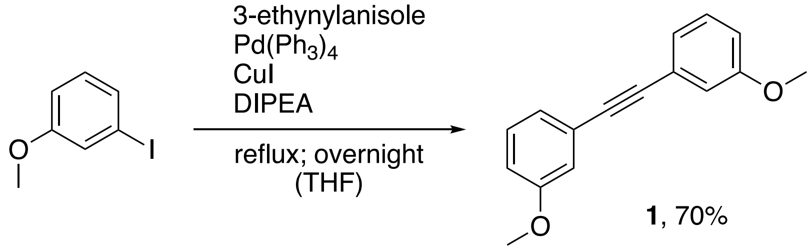

DIPEA (2.17 ml, 12.7 mmol, 3.0 eq.) was added to a mixture of 3-iodoanisole (509 µl, 4.23 mmol, 1.0 eq.), 3-ethynylanisole (672 µl, 5.08 mmol, 1.2 eq.), copper(I) iodide (81.0 mg; 423 µmol; 0.1 eq.) and tetrakis(triphenylphosphin)palladium(0) (244 mg, 212 µmol, 0.05 eq.) in 21 ml anhydrous THF and the resulting reaction mixture heated to reflux overnight. The reaction was cooled to room temperature and the solvent removed under reduced pressure. The crude product was purified by flash column chromatography (pentane : ethyl acetate = 99:1). The resulting product was further purified by recrystallization. Therefore, it was dissolved in minimal amount of DCM, then a tenfold excess of pentane was added, and the solution stored at 4 °C overnight. The crystals were washed with cold pentane, dried under reduced pressure and that yielded the product as colorless crystals in 70% yield (0.72 g, 3.02 mmol).
^{1}H NMR (300 MHz, $CDCl_3$) δ = 7.29 – 7.23 (t, ^3J = 7.9 Hz, 2H), 7.13 (dt, ^3J = 7.6, ^4J = 1.2 Hz, 2H), 7.07 (dd, ^4J = 2.6, 1.4 Hz, 2H), 6.90 (ddd, ^3J = 8.3, ^4J = 2.6, 1.0 Hz, 2H), 3.83 (s, 6H).
MS(ESI), *m/z* calculated for $C_{16}H_{13}O_2$: 238.10; found: 239.1[M+H]$^+$.
R_f= 0.42 (pentane : ethyl acetate = 98:2)
The characterization is in agreement with the literature.[1]

(Z)-3,3′-Dimethoxystilbene[1]

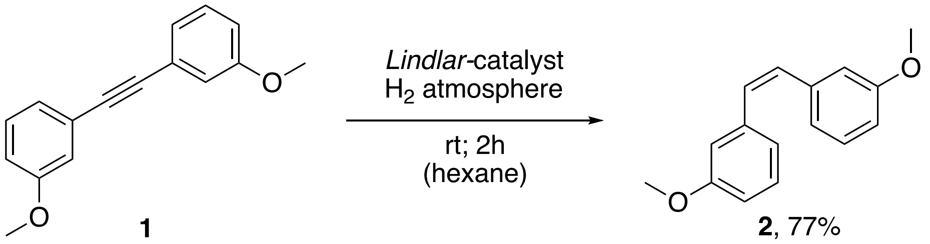

1 (816 mg, 3.42 mmol, 1.0 eq.) and *Lindlar*-catalyst (163 mg, 20% w/w of **1**) were stirred under H_2 atmosphere for 1.5 hours in 21 mL hexane at rt. After that LCMS showed complete conversion of the starting material, the reaction

mixture was filtered, and the solvent removed under reduced pressure. The crude product was purified by flash column chromatography (pentane : ethyl acetate = 98:2). This way **2** was isolated as a mixture with the

corresponding alkane in 77% yield as a colorless oil (616 mg, 2.56 mmol). The product was used without further purification.

^{1}H NMR (300 MHz, $CDCl_3$) δ = 7.15 (d, ^3J = 7.8 Hz, 2H), 6.85 (dt, ^3J = 7.6, ^4J = 1.3 Hz, 2H), 6.82 – 6.80 (m, 2H), 6.75 (ddd, ^3J = 8.2, ^4J = 2.6, 0.9 Hz, 2H), 6.58 (s, 2H), 3.67 (s, 6H).
MS(ESI), *m/z* calculated for $C_{16}H_{15}O_2$: 240.12; found: 241.1 $[M+H]^+$.
R_f= 0.46 (pentane : ethyl acetate = 98:2)
The characterization is in agreement with the literature.[1]

Ethyl 2,3-bis(3-methoxyphenyl)cyclopropane-1-carboxylate

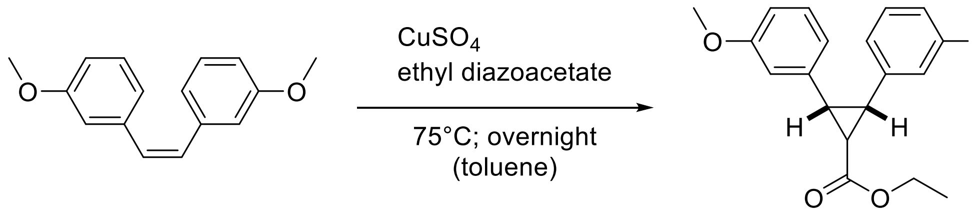

3, 17%

Ethyl diazoacetate (13% DCM, 1.41 g, 10.5 mmol, 2.5 eq.) was added to a mixture of copper(**II**) sulfate (40.0 mg, 0.25 mmol, 0.06 eq.) and **2** (1.01 g, 4.2 mmol, 1.0 eq.) in 6.8 mL anhydrous toluene over 4 hours at 75 °C and then further stirred overnight at that temperature. The reaction mixture was filtered and the solvent removed under reduced pressure. The crude product was purified by flash column chromatography (pentane : ethyl acetate = 98:2 → 92:8). This way the unreacted starting material was recovered (roughly 66%) and **3** was isolated as a mixture with diethyl maleate as a yellow oil in 18% yield (0.23 g, 0.71 mmol). The product was used without further purification.
^{1}H NMR (300 MHz, $CDCl_3$) δ = 7.05 (d, ^3J = 7.9 Hz, 2H), 6.66 (ddd, ^3J = 8.3, ^4J = 2.6, 0.9 Hz, 2H), 6.60 (dt, ^3J = 7.6, ^4J = 1.2 Hz, 2H), 6.47 (dd, ^4J = 2.4, 1.7 Hz, 2H), 4.24 (d, ^3J = 7.1 Hz, 2H), 3.62 (s, 6H), 3.02 (d, ^3J = 5.3 Hz, 2H), 2.51 (t, ^3J = 5.3 Hz, 1H), 1.32 (t, ^3J = 7.1 Hz, 3H).
^{13}C NMR (101 MHz, $CDCl_3$) δ = 173.4, 159.4, 137.4, 129.1, 121.5, 114.4, 112.5, 61.1, 55.2, 33.4, 27.7, 14.4.
MS(ESI), *m/z* calculated for $C_{20}H_{22}O_4$: 326.15; found: 327.2 $[M+H]^+$, 281.2 $[M-OEt]^+$.
HRMS(ESI), *m/z* $[M+H]^+$ calculated for $C_{20}H_{22}O_4$: 327.1596; found: 327.1591.
R_f= 0.57 (pentane : ethyl acetate = 9:1)

(2,3-bis(3-methoxyphenyl)cyclopropyl)methanol

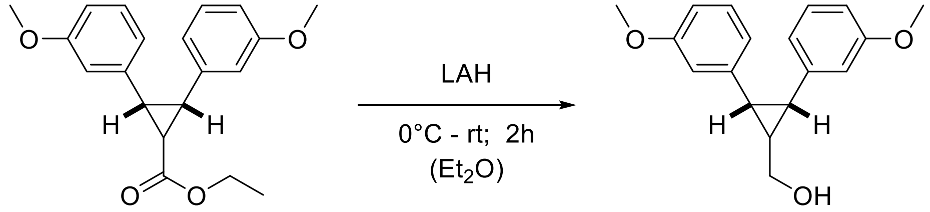

3 **4**, 68%

$LiAlH_4$ (1 M in Et_2O, 1.45 ml, 1.45 mmol, 2.0 eq.) was added dropwise to a solution of **3** (237 mg, 726 µmol, 1.0 eq.) in 6.9 mL anhydrous diethyl ether at 0 °C. The reaction was stirred at room temperature for 2 hours until TLC showed full conversion of starting material. The reaction was quenched by addition of 3 mL isopropanol and 5 mL water. The resulting suspension was filtered over celite, the organic phase washed with 5 mL brine, dried over Na_2SO_4 and the solvent removed under reduced pressure. The crude product was purified by flash column chromatography (dichloromethane : methanol = 99:1) and the **4** isolated as a yellow oil in 68 % yield (140 mg, 494 µmol).

^{1}H NMR (300 MHz, $CDCl_3$) δ = 7.04 (t, ^3J = 7.9 Hz, 2H), 6.65 – 6.57 (m, 4H), 6.46 (dd, ^4J = 2.4, 1.7 Hz, 2H), 3.84 (d, ^3J = 6.5 Hz, 2H), 3.62 (s, 6H), 2.39 (d, ^3J = 5.7 Hz, 2H), 2.12 – 2.01 (m, 1H), 1.62 (bs, 1H).

^{13}C NMR (75 MHz, $CDCl_3$) δ = 159.3, 139.2, 128.9, 121.6, 114.5, 111.9, 66.3, 55.2, 29.8, 28.3.

MS(ESI), *m/z* calculated for $C_{18}H_{20}O_3$: 284.14; found: 285.2 $[M+H]^+$, 307.1 $[M+Na]^+$, 267.1 $[M-OH]^+$.

HRMS(ESI), *m/z* $[M+H]^+$ calculated for $C_{18}H_{20}O_3$: 285.1491; found: 285.1485.

R_f= 0.47 (dichloromethane : methanol = 98:2)

(2,3-bis(3-methoxyphenyl)cyclopropyl)methyl acetate

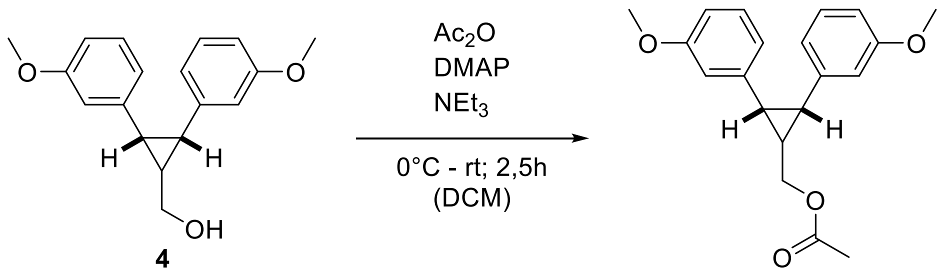

4 **5**, 70%

Acetic anhydride (68.0 µL, 711 µmol, 2.6 eq.) was added to a solution of **4** (79.0 mg, 0.277 mmol, 1.0 eq.), DMAP (2.0 mg, 14.0 mmol, 0.05 eq.) and triethylamine (189 µL, 1.40 mmol, 4.9 eq.) in 2 mL anhydrous DCM at 0 °C. The reaction was stirred for 2 hours at room temperature until TLC showed full conversion of starting material. The organic phase was washed with 2 mL 1 M HCl and 2 mL brine, dried over Na_2SO_4 and the solvent removed under reduced pressure. The crude product was purified by flash column chromatography (pentane : ethyl acetate = 95:5 → 90:10) and **5** isolated as yellow oil in 70% yield (58.0 mg, 178 µmol).

^{1}H NMR (300MHz, $CDCl_3$) δ = 7.04 (t, ^3J = 7.9 Hz, 2H), 6.63 (ddd, ^3J = 8.3, ^4J = 2.6, 0.9 Hz, 2H), 6.58 (dt, ^3J = 7.6, ^4J = 1.2 Hz, 2H), 6.44 (dd, ^4J = 2.4, 1.7 Hz, 2H), 4.26 (d, ^3J = 7.0 Hz, 2H), 3.62 (s, 6H), 2.43 (d, ^3J = 5.6 Hz, 2H), 2.13 – 2.07 (m, 4H).

^{13}C NMR (75 MHz, $CDCl_3$) δ = 171.4, 159.3, 138.9, 128.9, 121.6, 114.5, 111.9, 67.8, 55.2, 30.2, 24.6, 21.2.
MS(ESI), *m/z* calculated for $C_{20}H_{22}O_4$: 326.15; found: 349.1 $[M+Na]^+$, 267.1 $[M-OAc]^+$.
HRMS(ESI), *m/z* $[M+Na]^+$ calculated for $C_{20}H_{22}O_4$: 349.1416; found: 349.1410.
R_f= 0.28 (pentane : ethyl acetate = 9:1)

(3,9-dimethoxy-6-oxo-1,1a,6,10b-tetrahydrodibenzo[a,e]dicyclopropa[c,g][8]annulen-1-yl)methyl acetate (photo-DMBO)

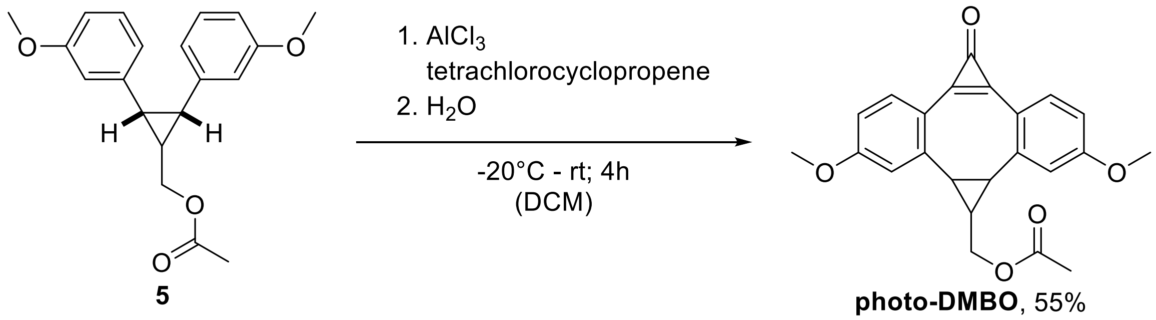

5 **photo-DMBO**, 55%

In a flame dried flask $AlCl_3$ (147 mg, 1.10 mmol, 3.0 eq.) and tetrachlorocyclopropene (54.0 mL, 441 µmol, 1.2 eq.) were stirred in 5.5 mL anhydrous DCM for 20 minutes at room temperature. The reaction mixture was cooled to -20 °C and a solution of **5** (120 mg, 368 µmol, 1.0 eq.) in 1.8 mL DCM was added dropwise. The reaction was stirred for 1 hour at -20 °C, then warmed to room temperature over 2 hours and the reaction stirred for an additional hour at room temperature. The reaction mixture was quenched with 5 mL water and stirred for 30 minutes. The aqueous phase was extracted with DCM (5 × 5 mL), the combined organic phases washed with 20 ml brine, dried over Na_2SO_4 and the solvent removed under reduced pressure. The crude product was purified by flash column chromatography (dichloromethane : methanol = 98:2) and **photo-DMBO** isolated as yellow oil in 55% yield (76.0 mg, 202 µmol).
^{1}H NMR (300 MHz, $CDCl_3$) δ = 7.70 (d, ^3J = 8.5 Hz, 2H), 7.12 (d, ^4J = 2.5 Hz, 2H), 6.87 (t, ^3J = 8.5, ^4J = 2.5 Hz, 2H), 4.38 (d, ^3J = 6.9 Hz, 2H), 3.86 (s, 6H), 2.38 (d, ^3J = 6.9 Hz, 2H), 2.17 (s, 3H), 2.01 (p, ^3J = 6.9 Hz, 1H).
^{13}C NMR (75 MHz, $CDCl_3$) δ = 171.1, 161.7, 153.1, 147.3, 142.3, 133.3, 118.6, 117.6, 112.8, 68.4, 55.6, 31.4, 28.3, 21.2.
MS(ESI), *m/z* calculated or $C_{23}H_{20}O_5$: 376.13; found: 377.1 $[M+H]^+$, 753.2 $[2M+H]^+$.
HRMS(ESI), *m/z* $[M+H]^+$ calculated for $C_{23}H_{20}O_5$: 377.1389; found: 377.1384.
R_f= 0.38 (dichloromethane : methanol = 98:2)

Synthesis of photo-DIBO

1,2-bis(3-methoxyphenyl)ethane

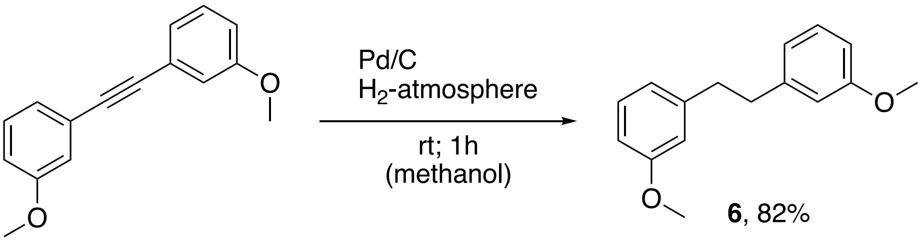

6, 82%

1 (266 mg, 1.12 mmol, 1.0 eq.) and palladium on charcoal (53.2 mg, 20 wt%) were added to 15 mL methanol. The reaction was then stirred for 1.5 hours under H_2-atmosphere. The reaction mixture was filtered, and the solvent removed under reduced pressure. The crude product was purified by column chromatography (pentane : ethyl acetate = 98:2) and **6** isolated as colorless oil in 82% yield (219 mg, 904 µmol).
^{1}H NMR (300 MHz, $CDCl_3$) δ = 7.20 (td, ^3J = 7.5, ^4J = 1.2 Hz, 2H), 6.82 – 6.72 (m, 6H), 3.79 (s, 6H), 2.90 (s, 4H).
MS(ESI), *m/z* calculated for $C_{16}H_{18}O_2$: 242.13; found: 243.1 $[M+H]^+$.

4,9-dimethoxy-6,7-dihydro-1H-dibenzo[a,e]cyclopropa[c][8]annulen-1-one (photo-DIBO)

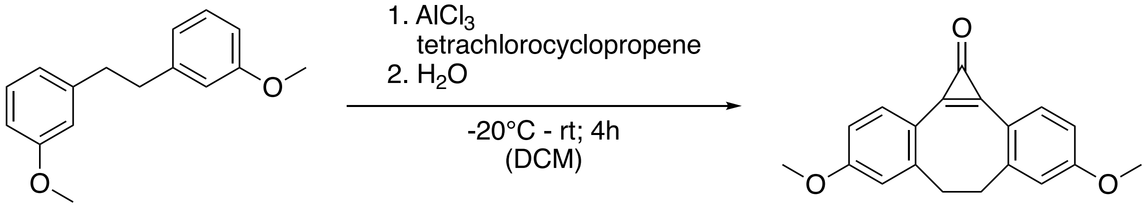

photo-DIBO, 56%

In a flame dried flask $AlCl_3$ (180 mg, 1.36 mmol, 1.5 eq.) and tetrachlorocyclopropene (111 µL, 904 mol, 1.0 eq.) were stirred in 9 mL anhydrous DCM for 20 minutes at room temperature. The yellow reaction mixture was cooled to –78 °C in a dry ice/isopropanol cold bath and a solution of **6** (219 mg, 904 µmol, 1.0 eq.) in 9 mL DCM was added dropwise over 15 minutes. The reaction was stirred for 2 hours and then TLC showed full conversion of the starting material. The cold bath was removed, 4 ml water carefully added using a Pasteur pipette and the reaction warmed to room temperature. The yellow mixture was transferred into a separatory funnel and washed twice with 20 ml water. The combined aqueous phase was extracted with 20 ml DCM once and the combined organic phases dried over Na_2SO_4, filtered and the solvent removed under reduced pressure. The crude product was purified by flash column chromatography (50 – 100% EtOAc in DCM) and **photo-DIBO** isolated as a colorless solid in 56% yield (148 mg, 506 µmol).
^{1}H NMR (500 MHz, $CDCl_3$) δ = 8.07 (d, ^3J = 8.3 Hz, 2H), 6.99 – 6.86 (m, 4H), 3.90 (s, 6H), 3.37 (d, ^3J = 10.7 Hz, 2H), 2.66 (d, ^3J = 10.7 Hz, 2H).
MS(ESI), *m/z* calculated for $C_{19}H_{16}O_3$: 292,11; found: 293.1 $[M+H]^+$.

<u>Synthesis of photo-ODIBO</u>

(3-((*tert*-butyldimethylsilyl)oxy)phenyl)methanol

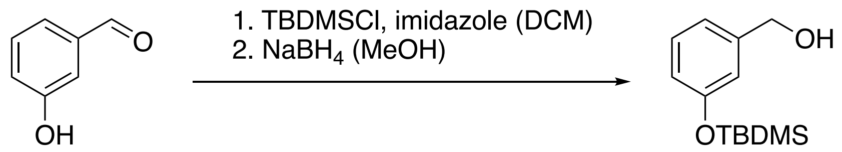

7; 97%

tert-Butyldimethylsilyl chloride (1.48 g, 9.80 mmol, 1.2 eq.) was added to a solution of 3-Hydroxybenzaldehyde (1.00 g, 8.19 mmol, 1.0 eq.) and imidazole (1.67 g, 24.6 mmol, 3.0 eq.) in 13 ml DCM. The reaction turned orange and was stirred for 1 hour until TLC showed full conversion of the starting material. The reaction mixture was washed with 15 ml 10% citric acid solution and 15 ml brine, dried over Na_2SO_4 and the solvent removed under reduced pressure. The crude product was dissolved in 25 ml methanol cooled to 0 °C and sodium borohydride (310 mg, 8.19 mmol, 1.0 eq.) was added in three portions over 30 minutes. The solution was stirred at that temperature for 1 hour until TLC showed full conversion of the starting material and 6 ml water were added to the reaction. The organic solvent was removed under reduced pressure and the product extracted with ethyl acetate (3 × 5 ml), dried over Na_2SO_4 and the solvent removed under reduced pressure. The crude product was purified by column chromatography (pentane : ethyl acetate = 85:15) and **7** isolated as a colorless oil in 97% yield (1.89 g, 7.94 mmol) over two steps.

^{1}H NMR (500 MHz, $CDCl_3$) δ = 7.21 (t, ^3J = 7.8 Hz, 1H), 6.94 (d, ^3J = 7.6 Hz, 1H), 6.88 – 6.83 (m, 1H), 6.76 (dd, ^3J = 8.0, ^4J = 2.1 Hz, 1H), 4.64 (s, 2H), 1.62 (bs, 1H), 0.99 (s, 9H), 0.20 (s, 6H).

MS(ESI), *m/z* calculated for $C_{13}H_{22}O_2Si$: 238.14; found: 221.2 $[M-OH]^+$.

R_f= 0.25 (pentane : ethyl acetate = 85:15).

***tert*-butyl(3-((4-(*tert*-butyl)phenoxy)methyl)phenoxy)dimethylsilane**

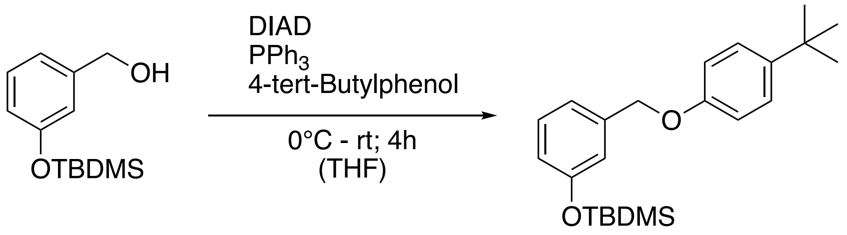

8; 84%

Diisopropyl azodicarboxylate (1.44 ml, 7.34 mmol, 1.0 eq.) was added at 0 °C to a solution of **7** (1.75 g, 7.34 mmol, 1.0 eq.), 4-*tert*-butylphenol (1.10 g, 7.34 mmol, 1.0 eq.) and triphenylphosphin (1.93 g, 7.34 mmol, 1.0 eq.) in 17 ml dry THF. The light yellow reaction solution was stirred for 4h and slowly warmed to room temperature during that time. After that TLC showed full conversion of the starting material, silica was added and the solvent removed under reduced pressure. The crude product was purified by column chromatography (pentane : ethyl acetate = 95:5) and **8** isolated as a colorless oil in 84% yield (2.28 g, 6.14 mmol).

^{1}H NMR (500 MHz, $CDCl_3$) δ = 7.33 – 7.26 (m, 2H), 7.23 (t, ^{3}J = 7.8 Hz, 1H), 7.01 (d, ^{3}J = 7.6 Hz, 1H), 6.93 – 6.87 (m, 3H), 6.78 (dd, ^{3}J = 7.9, ^{4}J = 1.6 Hz, 1H), 5.00 (s, 2H), 1.30 (s, 9H), 0.98 (s, 9H), 0.18 (s, 6H).
^{13}C NMR (75 MHz, $CDCl_3$) δ = 156.7, 156.0, 143.7, 139.0, 129.7, 126.4, 120.4, 119.6, 119.2, 114.5, 69.9, 34.2, 31.7, 25.8, 18.4, -4.3.
R_f= 0.83 (pentane : ethyl acetate = 95:5)

3-((4-(*tert*-butyl)phenoxy)methyl)phenol

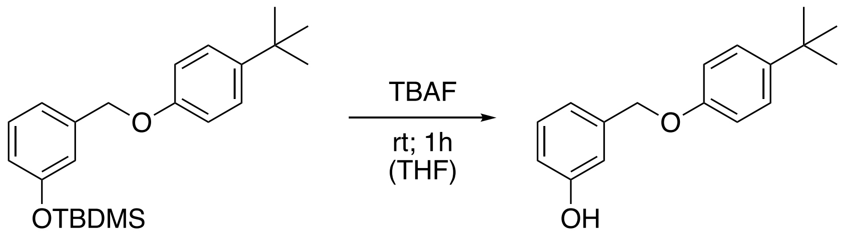

8 **9**, 91%

TBAF (6.14 ml 1 M in THF, 6.14 mmol, 1.0 eq.) was added to a solution of ***8*** (2.28 g, 6.14 mmol, 1.0 eq.) in 25 ml THF and stirred at room temperature for 1.5 hour until TLC showed full conversion off the starting material. The solvent was removed under reduced pressure and the crude product dissolved in 15 ml DCM and washed with 15 ml water. The aqueous phase is extracted once with 10 ml DCM, the combined organic phases dried over Na_2SO_4 and the solvent removed under reduced pressure. The crude product is purified by column chromatography (pentane : ethyl acetate = 92:8) and the product isolated as a colorless oil in 91% yield (1.43 g, 5.59 mmol).
^{1}H NMR (500 MHz, $CDCl_3$) δ = 7.33 – 7.29 (m, 2H), 7.25 (t, ^{3}J = 7.9 Hz, 1H), 6.99 (d, ^{3}J = 7.6 Hz, 1H), 6.95 – 6.88 (m, 3H), 6.78 (dd, ^{3}J = 8.1, ^{4}J = 2.4 Hz, 1H), 5.01 (s, 2H), 4.95 (s, 1H), 1.30 (s, 9H).
^{13}C NMR (75 MHz, $CDCl_3$) δ = 156.6, 155.9, 145.3, 137.1, 130.8, 124.8, 119.1, 115.4, 114.4, 114.3, 69.2, 34.2, 31.7.
R_f= 0.29 (pentane : ethyl acetate = 92:8)

Photo-ODIBO[2]

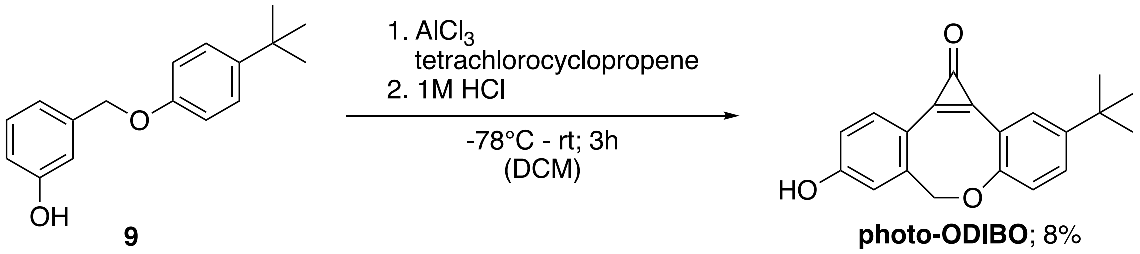

9 **photo-ODIBO**; 8%

Tetrachlorocyclopropene (96 µl; 780 µmol; 1.0 eq.) was added to a suspension of aluminium chloride (156 mg; 1.17 mmol; 1.5 eq.) in 8 ml DCM and stirred for 20 minutes at room temperature. The orange reaction was cooled to -78 °C and **9** (200 mg; 780 µmol; 1.0 eq.) in 8 ml was added dropwise. The reaction turned dark green and was stirred at -78 °C for 3 hours, afterwards warmed to room temperature and stirred for another 45 minutes. The reaction was quenched by addition of 12 ml 1 M HCl, the organic phase separated, and the aqueous phase extracted with DCM (5 × 8 ml), the combined organic phases dried over Na_2SO_4 and the solvent removed under

reduced pressure. The crude product was purified by column chromatography (pentane : ethyl acetate = 1:1 -> 3:7) and the product isolated as a colorless solid in 8% yield (19.1 mg; 62.4 µmol).

^{1}H NMR (400 MHz, DMSO) δ = 10.71 (s, 1H), 7.79 – 7.72 (m, 2H), 7.62 (dd, ^3J = 8.6, 2.6 Hz, 1H), 7.28 (d, ^3J = 8.5 Hz, 1H), 7.08 (d, ^4J = 2.5 Hz, 1H), 6.98 (dd, $^{3,4}J$ = 8.3, 2.5 Hz, 1H), 5.30 (d, ^3J = 12.2 Hz, 1H), 4.83 (d, ^3J = 12.2 Hz, 1H), 1.33 (s, 9H).

MS(ESI), *m/z* calculated for $C_{20}H_{18}O_3$: 306.13; found: 307.1 $[M+H]^+$.

Synthesis of azides and tetrazine

2-Azidoethanol (pAz)

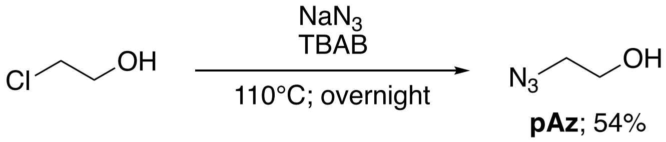

Sodium azide (845 mg; 15 mmol; 1.3 eq.), tetrabutylammonium bromide (97 mg; 0.3 mmol; 0.03 eq.) were added to 2-chloroethanol and heated to 110 °C overnight. The slightly yellow reaction was cooled to room temperature filtered and the solid washed with diethyl ether. The ether was removed under reduced pressure (careful product is slightly volatile) and the product isolated as a colorless liquid in 54% yield (474 mg; 5.44 mmol).

^{1}H NMR (400 MHz, $CDCl_3$) δ = 3.82 – 3.75 (m, 2H), 3.49 – 3.42 (m, 2H), 1.77 (s, 1H).

2-Azido-2-dimethylpropanol (tAz)

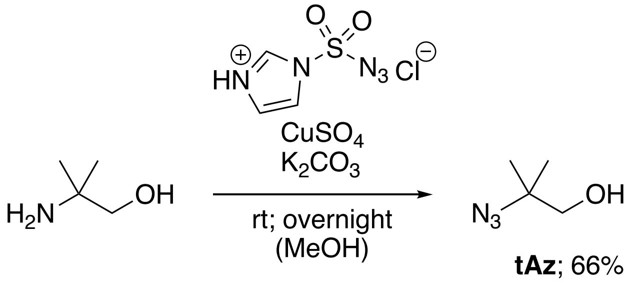

2-Amino-2-methylpropanol (1.00 g; 10.1 mmol; 1.0 eq.), potassium carbonate (1.67 g; 12.1 mmol; 1.2 eq.), and copper(II) sulfate (16 mg; 0.1 mmol; 0.01 eq.) were given into 42 ml methanol. Imidazole-1-sulfonyl azide hydrochloride[3] (2.7 g; 12.1 mmol; 1.2 eq.) was added portion-wise to the reaction and stirred at room temperature overnight. The solvent was removed under reduced pressure und the brown crude product was dissolved in water, acidified with 3 M HCl to pH=3 and extracted three times with ethyl acetate. The collected organic phases were dried over sodium sulfate, the solvent removed under reduced pressure and the crude product purified via column chromatography (pentane : ethyl acetate = 2:1). This way **tAz** was isolated as a colorless liquid in 66% yield (765 mg, 6.65 mmol).

^{1}H NMR (300 MHz, $CDCl_3$) δ = 3.44 (s, 2H), 1.77 (bs, 1H), 1.30 (s, 6H).

3,6-Dimethyltetrazine

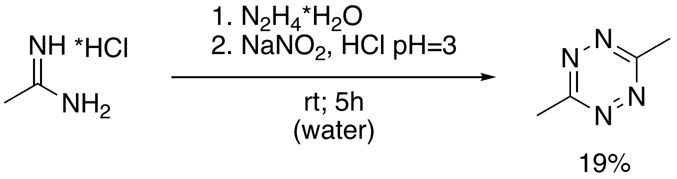

Hydrazine monohydrate (1.03 ml; 21.2 mmol; 2.0 eq.) was added dropwise to a solution of acetamidine hydrochloride (1.0 g, 10.6 mmol, 1.0 eq.) in 5 ml water. The reaction was stirred at room temperature for 5 hours and turned red over time. Then the reaction was diluted with an additional 5 ml of water, sodium nitrite (3.5 g, 53.0 mmol, 5.0 eq.) was added and the reaction cooled in an ice bath. 6 M HCl was added dropwise until pH=3 was reached (caution nitrogen oxides are produced) and stirred for additional 15 minutes. The solution is transferred to a separation funnel and extracted three times with dichloromethane, the combined organic phases are dried over sodium sulfate and the solvent as good as possible removed under reduced pressure (product volatile). The crude product was obtained as a violet oil which still contained residual solvent and therefore pentane was added which crystallized the product as a pink solid. After drying on air, the product was obtain in 19% yield (219 mg, 1.99 mmol).

^{1}H NMR (500 MHz, $CDCl_3$) δ = 3.03 (s, 6H).

Handling and storage of cyclopropenones

All cyclopropenones were stored at -20 °C in screw cap vials and protected from light by aluminum foil. For all experiments 5 mM stock solutions in MeOH were prepared.

LC-MS Assays

All LC-MS samples contained a final concentration of 250 µM of the corresponding cyclopropenone, the strained alkyne or the reaction product. The resulting products and reaction mixtures were analyzed using LC-MS showing the absorption trace at 254 nm (Supplementary Fig. S1-S4 and S7-S10).

Photo Deprotection

Photo deprotection was carried out in a 4 °C cold room. 50 µl of the 5 mM cyclopropenone stock solutions in methanol were mixed with 450 µl methanol in a 12 well dish to get a final concentration of 500 µM and the well dish covered with its lid. The solution was irradiated under stirring with UV-light of 365 nm wavelength (Vilber Lourmat VL-215.L UV lamp (2 x 15W 365 nm lamps); distance to reaction roughly 8 cm) for 5, 6, or 10 minutes for **photo-DIBO**, **photo-ODIBO** and **photo-DMBO** respectively. The completion of the reaction was checked via LC-MS by mixing 20 µl reaction solution with 20 µl of methanol to reach a final concentration of 250 µM (see Supplementary Fig. S1a), S2a), and S4a). Afterwards the reaction solution was transferred into an Eppendorf tube and used for further reactions within one hour.

DMBO: **MS(ESI)**, *m/z* calculated for $C_{22}H_{20}O_4$: 348.14; found: 349.1 $[M+H]^+$.
DIBO: **MS(ESI)**, *m/z* calculated for $C_{18}H_{16}O_2$: 264.12; found: 265.1 $[M+H]^+$.
ODIBO: **MS(ESI)**, *m/z* calculated for $C_{19}H_{18}O_2$: 278.13; found: 279.1 $[M+H]^+$.

Synthesis of iEDDAC products

50 µl of a 500 µM solution of strained alkyne in methanol was mixed with 12.5 µl of 20 mM **MeMe-Tet** solution in methanol and reacted at room temperature for 40 hours. The reaction was then diluted to a total volume of 100 µl (250 µM based on strained alkyne) and analyzed by LC-MS (Supplementary Fig. S1-S4).

DMBO: **+MeMe-Tet MS(ESI)**, *m/z* calculated for $C_{26}H_{26}N_2O_4$: 430.19; found: 431.2 $[M+H]^+$ (quantitative conversion)

DIBO: **+MeMe-Tet MS(ESI)**, *m/z* calculated for $C_{22}H_{22}N_2O_2$: 346.17; found: 347.2 $[M+H]^+$ (conversion <1 %)

ADIBO: **+MeMe-Tet MS(ESI)**, *m/z* calculated for $C_{22}H_{22}N_4O$: 358.18; not found

ODIBO: **+MeMe-Tet MS(ESI)**, *m/z* calculated for $C_{23}H_{24}N_2O_2$: 360.18; found: 361.2 $[M+H]^+$ (conversion <1 %)

Synthesis of SPAAC products

50 µl of a 500 µM solution of strained alkyne in methanol was mixed with 2.5 µl of 100 mM **pAz** or 10 µl of 100 mM **tAz** solution in methanol and reacted at room temperature for 24 hours (**pAz**) or 72 hours (**tAz**; 120 h for **DIBO**). The reaction was then diluted to a total volume of 100 µl (250 µM based on strained alkyne) and analyzed by LC-MS (Supplementary Fig. S7-S10).

DMBO: **+pAz MS(ESI)**, *m/z* calculated for $C_{24}H_{25}N_3O_5$: 435.18; found: 436.2 $[M+H]^+$.
+tAz MS(ESI), *m/z* calculated for $C_{26}H_{29}$ N_3O_5: 463.21; found: 464.2 $[M+H]^+$.

ADIBO: **+pAz MS(ESI)**, *m/z* calculated for $C_{20}H_{21}N_5O_2$: 363.17; found: 364.2 $[M+H]^+$.
+tAz MS(ESI), *m/z* calculated for $C_{22}H_{25}N_5O_2$: 391.20; found: 392.2 $[M+H]^+$.

DIBO: **+pAz MS(ESI)**, *m/z* calculated for $C_{20}H_{21}N_3O_3$: 351.16; found: 352.2 $[M+H]^+$.
+tAz MS(ESI), *m/z* calculated for $C_{22}H_{25}N_3O_3$: 379.19; found: 380.2 $[M+H]^+$.

ODIBO: **+pAz MS(ESI)**, *m/z* calculated for $C_{21}H_{23}N_3O_3$: 365.17; found: 366.2 $[M+H]^+$.
+tAz MS(ESI), *m/z* calculated for $C_{23}H_{27}N_3O_3$: 393.21; found: 394.2 $[M+H]^+$.

Kinetics

UV-Vis characterization of cyclooctynes and reaction products

For all characterizations the final concentration of cylcooctynes and the corresponding reaction product was 50 µM in methanol. All spectra were recorded in methanol at room temperature. The spectra are shown in Supplementary Fig. S5.

Kinetic Measurements and data evaluation

All reactions were conducted in methanol and the final concentration of cylcooctyne was 50 µM in a total volume of 200 µl. The second-order rate constants were measured under pseudo first-order conditions with excess of azides or tetrazines (> 50 eq.). All solutions were transferred using Hamilton syringes with a total volume of 25, 100 or 500 µl.

For all reactions, stock solutions of 500 µM strained alkyne, 100 mM **pAz**, 200 mM **tAz** and 100 mM **MeMe-Tet** in methanol were used. First methanol or PBS and the corresponding azide/tetrazine were mixed in a quartz glass cuvette to a total volume of 180 µl and thoroughly mixed using a 100 µl Eppendorf pipette. This solution was used to measure the baseline absorption at the characteristic absorption maximum of the corresponding cyclooctyne (**DMBO**: 327 nm; **DIBO**: 320 nm; **ADIBO**: 309 nm; **ODIBO**: 322 nm). Then 20 µl of the corresponding strained cyclooctyne were added and the reaction again thoroughly mixed using an Eppendorf pipette. Afterwards the cuvette was capped, and the reaction followed by measuring the decay of absorption at the characteristic absorption maximum of the corresponding cyclooctyne. Data points were taken every 2 s (for **pAz** and **MeMe-Tet** reactions) and every 10 s (for **tAz**) and all reactions performed in triplicates.

The data evaluation was done using Prism software (GraphPad software Prism 9) assuming pseudo first-order conditions. Non-linear regression of the exponential decay gave different observed first-order rate constants k_{obs} which were then plotted against the concentration of the used azide/tetrazine. Linear regression (Prism software) gave the line of best fit, and its slope describes the second-order rate constant k_2 of the reaction.

Computational Methods and Results

DFT calculations were performed using Gaussian 16 Revision A.03.[4] The M06-2X functional[5] in combination with the 6-311+G(d,p) basis set,[6] Grimme's D3 empirical dispersion[7] as proposed by Grimme[8] and the SMD solvent model[9] were used. Stationary points were identified by no or exactly one imaginary frequency for minima and transition states, respectively. Conformer searches were performed for all structures using CREST 2.7.1[10] and XTB 6.2.[11] Default settings were applied. For transition states forming bonds were frozen to values obtained from an initially found transition state geometry. Entropies were corrected using the quasi-harmonic correction in GoodVibes[12] by setting all frequencies below 100 cm^{-1} to 100 cm^{-1}.

Energy decomposition analysis and distortion interaction analysis was performed using PyFrag[13] and ADF 2018[14-16] with the M06-2X-D3 functional, TZ2P basis set,[17] and a numerical quality of VeryGood in the gas phase using geometries obtained from SMD calculations in Gaussian. Distortion interaction analysis results from ADF were compared to those obtained from Gaussian using autoDIAS[18] to ensure no discrepancies between software packages.

Discussion on the used DFT Method

The density functional M06-2X consistently performs among the best hybrid meta-GGA functionals for reaction barriers. In combination with Pople basis sets, it has been successfully employed to study cycloadditions of strained alkenes and alkynes. In particular, it seems to be well suited to reproduce relative barrier heights. In published and unpublished work, we also found M06-2X to reproduce absolute barriers of such reactions reasonably well, a generally challenging task. In similar systems, M06-2X seems to slightly overestimate the entropic contribution to the absolute barrier by 2 kcal/mol. In this study, the calculated absolute barrier for the reaction of DMBO* with MeMe-Tet was calculated to be 20.3 kcal/mol while the measured rate constant corresponds to a barrier of 18.4 kcal/mol, again overestimating the barrier by 2 kcal/mol. Our calculated entropic contribution ($-T\Delta S$) at 298K was found to be -16.3 kcal/mol, a typical value for calculations this kind of Diels–Alder cycloadditions and comparable to previous calculations. As we are interested in drawing mechanistic insights from relative electronic barriers of activation, an overestimation of entropic contributions does not affect our analysis. The chosen computational method can therefore be considered as a good choice for this investigation.

Supplementary Tables S1-S8

Table S1 shows the results of the distortion/interaction and energy decomposition analysis of the transition states of **DIBO*** and **DMBO*** with **MeMe-Tet**. The analysis was performed in ADF using M06-2X-D3/TZ2P on Gaussian M06-2X-D3/6-311+G(d,p) SMD(water) calculated structures. For **DIBO*** both TS show similar electronic barriers. Distortion energy is considerably lower for the face approach, while at the same time Pauli repulsion is lowered as well, demonstrating the reduced steric interaction in this geometry. On the other hand, orbital interactions are also lower, which can be explained by a worse alignment of the triple bond with the tetrazine diene. For **DMBO*** only the *face* approach is found. Compared to **DIBO*** the distortion energy and Pauli repulsion are again considerably lower and are the main contributors to the lowered activation barrier when reacting with **Me-Me-Tet**.

Table S1: Distortion/interaction and energy decomposition analysis of **DIBO*-TS$_{edge}$**, **DIBO*-TS$_{face}$**, and **DMBO*-TS** at the respective transition state. All values are given in kcal mol^{-1}.

Structure	$\Delta E^{‡}$	$\Delta E^{‡}_{int}$	$\Delta E^{‡}_{dist}$	$\Delta E^{‡}_{Pauli}$	$\Delta E^{‡}_{OI}$	$\Delta V^{‡}_{elstat}$	$\Delta E^{‡}_{disp}$
**DIBO*-TS$_{edge}$**	15.87	-22.16	**38.03**	**119.64**	**-71.76**	-68.88	-1.15
**DIBO*-TS$_{face}$**	16.28	-16.76	**33.04**	**79.49**	**-46.72**	-48.4	-1.15
**DMBO*-TS**	6.86	-14.94	**21.80**	**67.08**	**-37.92**	-42.9	-1.19

Table S2 lists calculated energies for the reactions between **MeMe-Tet** and cyclooctynes.

Table S2: M06-2X-D3/6-311+G(d,p) SMD(water) calculated energies for the reaction of **MeMe-Tet** with cyclooctynes. All values are given in hartree.

Structure	ΔE (hartree)	ZPE (hartree)	ΔH_{298} (hartree)	ΔG_{298} (hartree)
**DIBO***	-616.764277	0.229275	-616.522476	-616.572013
**DMBO***	-654.825103	0.234673	-654.577204	-654.628098
**MeMe-Tet**	-374.923874	0.106761	-374.808358	-374.848928
**DIBO*-TS$_{edge}$**	-991.665682	0.339285	-991.306884	-991.369497
**DIBO*-TS$_{face}$**	-991.667858	0.338435	-991.309491	-991.373353
**DMBO*-TS**	-1029.742630	0.342831	-1029.379019	-1029.444560
**4C-DIBO***	-694.123173	0.263853	-693.845126	-693.898137
**5C-DIBO***	-733.452007	0.294027	-733.142944	-733.197626
**6C-DIBO***	-772.763030	0.323785	-772.423349	-772.479554
**4C-DIBO*-TS**	-1069.043295	0.372504	-1068.649112	-1068.716477
**5C-DIBO*-TS**	-1108.367591	0.402086	-1107.942672	-1108.012102
**6C-DIBO*-TS**	-1147.666763	0.431495	-1147.211306	-1147.282890
**MeO-DMBO***	-883.856760	0.300295	-883.538103	-883.599586
**MeO-DMBO*-TS**	-1258.774915	0.408427	-1258.340515	-1258.416378

Table S3 shows a DIA/EDA analysis of the reaction of **DMBO*** and **DIBO*** with **MeMe-Tet** investigating the effect of asynchronicity. Therefore DIA/EDA was conducted using two consistent geometries. First, both forming bond lengths were set to 2.32247 Å, the bond length encountered in the synchronous **DMBO*** transition state. Then the forming bond lengths were set to 2.22126 and 2.26285 Å, bond lengths encountered in the slightly asynchronous **DIBO*** transition state. In both the synchronous and asynchronous case **DIBO*** shows a considerably higher distortion energy compared to **DMBO***. In the asynchronous transition state, preferred by **DIBO***, distortion energy is also considerably increased compared to **DMBO***, while in the synchronous approach it is only slightly increased. This supports the data presented in Figure 2d.

Table S3: Distortion/interaction and energy decomposition analysis of synchronous and asynchronous transition states of **DIBO***and **DMBO*** with **MeMe-Tet**. All values are given in kcal mol^{-1}.

Structure	ΔE	ΔE_{int}	**ΔE_{dist}**	**ΔE_{Pauli}**	ΔE_{OI}	ΔV_{elstat}	ΔE_{disp}
**DMBO*-TS$_{synch}$**	6.91	-13.19	**20.1**	**60.7**	-34.13	-38.58	-1.18
**DIBO*-TS$_{synch}$**	14.58	-6.65	**21.23**	**68.35**	-31.85	-41.98	-1.17
**DMBO*-TS$_{asynch}$**	6.70	-20.09	**26.79**	**75.01**	-47.77	-28.86	-1.18
**DIBO*-TS$_{asynch}$**	16.26	-16.79	**33.05**	**79.49**	-46.72	-48.4	-1.15

Table S4 shows the result of an EDA analysis of **4C-DMBO***, **5C-DMBO***, and **6C-DMBO*** with **MeMe-Tet**. This analysis was performed at a consistent geometry with both forming bond lengths fixed at 2.32247 Å, essentially the distance found at the transition state for **DMBO*** with **MeMe-Tet**. This “consistent geometry" was chosen to eliminate any influence of the different lateness of the respective transition states and identify the underlying mechanism. Data shows that while ΔE_{OI}, ΔE_{Pauli}, ΔV_{elstat}, and ΔE_{disp} show little variation, ΔE_{dist} increases significantly with increased ring size. This highlights the importance of the small ring size which enforces a *cis*-configuration on the backbone C2 unit, allowing for lower energy access of the tub-like conformation.

Table S4: Distortion/interaction and energy decomposition analysis of **4C-DMBO***, **5C-DMBO***, and **6C-DMBO*** with **MeMe-Tet**. All values are given in kcal mol^{-1}.

Structure	ΔE	ΔE_{int}	**ΔE_{dist}**	ΔE_{Pauli}	ΔE_{OI}	ΔV_{elstat}	ΔE_{disp}
**4C-DMBO*-TS$_{2.322}$**	5.3	-14.1	**19.45**	60.8	-34.5	-39.2	-1.26
**5C-DMBO*-TS$_{2.322}$**	7.7	-15.2	**22.9**	60.2	-34.8	-39.3	-1.36
**6C-DMBO*-TS$_{2.322}$**	15.1	-16.0	**31.1**	59.6	-35.2	-38.9	-1.44

Table S5 lists energy barriers for the reactions between **pAz*** or **tAz*** and cyclooctynes. Difference in Gibbs Free Energy barriers between **tAz*** and **pAz*** reactions are approx. 4 kcal mol^{-1} for **DIBO***, **ODIBO***, and **ADIBO***. For **DMBO*** this difference is decreased to 2.1 kcal/mol.

Table S5: M06-2X-D3/6-311+G(d,p) SMD(methanol) calculated energy barriers of the reactions between azides and cyclooctynes. Differences of free energy barriers ($\Delta\Delta G_{298}^{‡}$) between the reaction of tAz and pAz.

Reaction between	$\Delta E^{‡}$ (kcal mol^{-1})	$\Delta G_{298}^{‡}$ (kcal mol^{-1})	$\Delta\Delta G_{298}^{‡}$ (kcal mol^{-1}) **tAz-pAz**
**DIBO* pAz***	8.2	23.2	4.0
**DIBO* tAz***	11.7	27.3	
**DMBO* pAz***	8.3	22.4	2.1
**DMBO* tAz***	9.8	24.5	
**ODIBO* pAz***	5.8	20.8	3.8
**ODIBO* tAz***	9.1	24.7	
**ADIBO* pAz***	6.9	22.0	4.2
**ADIBO* tAz***	10.4	26.2	

Table S6 lists calculated energies for the reactions between **pAz*** or **tAz*** and cyclooctynes.

Table S6: M06-2X-D3/6-311+G(d,p) SMD(methanol) calculated energies for the reaction of azides with cyclooctynes.

Structure	ΔE (hartree)	ZPE (hartree)	ΔH_{298} (hartree)	ΔG_{298} (hartree)
**DIBO***	-616.774368	0.229189	-616.532648	-616.582193
**DMBO***	-654.835473	0.234592	-654.587652	-654.638550
**ODIBO***	-652.672267	0.204768	-652.455176	-652.504363
**ADIBO***	-785.466616	0.255399	-785.195490	-785.251661
**pAz***	-243.369258	0.079430	-243.283285	-243.318171
**tAz***	-321.987883	0.135385	-321.843615	-321.883876
**DIBO*-pAz-TS**	-860.130569	0.310317	-859.801909	-859.863319
**DIBO*-tAz-TS**	-938.743544	0.365757	-938.356820	-938.422589
**DMBO*-pAz-TS**	-898.191576	0.314591	-897.857689	-897.921004
**DMBO*-tAz-TS**	-976.807766	0.370188	-976.415694	-976.483360
**ODIBO*-pAz-TS**	-896.032264	0.285963	-895.728157	-895.789313
**ODIBO*-tAz-TS**	-974.645717	0.341519	-974.283459	-974.348941
**ADIBO*-pAz-TS**	-1028.824847	0.336405	-1028.466861	-1028.534766
**ADIBO*-tAz-TS**	-1107.437953	0.392140	-1107.021721	-1107.093826

Table S7 shows the result of an EDA analysis on the reaction between **pAz*** and **tAz*** with **DMBO*** and **DIBO***. Due to the different asynchronicities in the observed transition states, a consistent geometry was used across all four systems using 2.25 Å for both forming bond lengths. In this case $\Delta E^{\ddagger}_{dist}$ is higher for **DMBO*** than **DIBO***. However, adopting the higher energy tub-like geometry allows for considerably reduced $\Delta E^{\ddagger}_{Pauli}$ in case of **DMBO*** compared to **DIBO*** for reactions with both **pAz*** and **tAz***. In addition, the steric repulsion in case of DMBO* is only increased by 2 kcal/mol when going from **pAz*** to **tAz*** while in case of **DIBO*** a 3.4 kcal/mol difference is observed. This highlights the reduced steric demand of **DMBO*** in the tub-like geometry.

Table S7: Results of EDA calculated in ADF using M06-2X-D3/TZ2P at Gaussian M06-2X-D3/6-311+G(d,p)-SMD(methanol) calculated geometries. All energies are given in kcal mol^{-1}.

Structure	$\Delta E^{\ddagger}_{int}$	**$\Delta E^{\ddagger}_{Pauli}$**	$\Delta E^{\ddagger}_{OI}$	$\Delta V^{\ddagger}_{elstat}$	$\Delta E^{\ddagger}_{disp}$	$\Delta E^{\ddagger}_{dist}$
**DMBO*-pAz*-TS$_{2.25}$**	-10.2	**63.66**	-32.46	-40.54	-0.85	20.00
**DIBO*-pAz*-TS$_{2.25}$**	-7.31	**67.61**	-31.08	-42.98	-0.87	16.02
**DMBO*-tAz*-TS$_{2.25}$**	-9.44	**65.66**	-32.54	-41.43	-1.13	20.18
**DIBO*-tAz*-TS$_{2.25}$**	-4.83	**71.05**	-30.03	-44.69	-1.15	15.93

Table S8 shows the result of an EDA analysis on the transition state geometries of **pAz*** and **tAz*** with **DMBO***. **pAz*** shows a lowered activation barrier due to lowered distortion energy. In addition, Pauli repulsion is considerably lower in case of **pAz***. Both point to a lowered steric hindrance.

Table S8: Results of EDA calculated in ADF using M06-2X-D3/TZ2P at Gaussian M06-2X-D3/6-311+G(d,p)-SMD(methanol) calculated geometries. All energies are given in kcal mol^{-1}.

Structure	$\Delta E^{\ddagger}_{int}$	$\Delta E^{\ddagger}_{Pauli}$	$\Delta E^{\ddagger}_{OI}$	$\Delta V^{\ddagger}_{elstat}$	$\Delta E^{\ddagger}_{disp}$	$\Delta E^{\ddagger}_{dist}$	$\Delta E^{\ddagger}_{dist,azide}$	$\Delta E^{\ddagger}_{dist,DMBO}$
**DMBO*-pAz*-TS**	-10.7	66.71	-34.21	-42.34	-0.85	20.41	14.17	6.24
**DMBO*-tAz*-TS**	-11.27	69.69	-36.29	-43.54	-1.13	22.2	14.88	7.32

Supplementary Figures S1 – S23

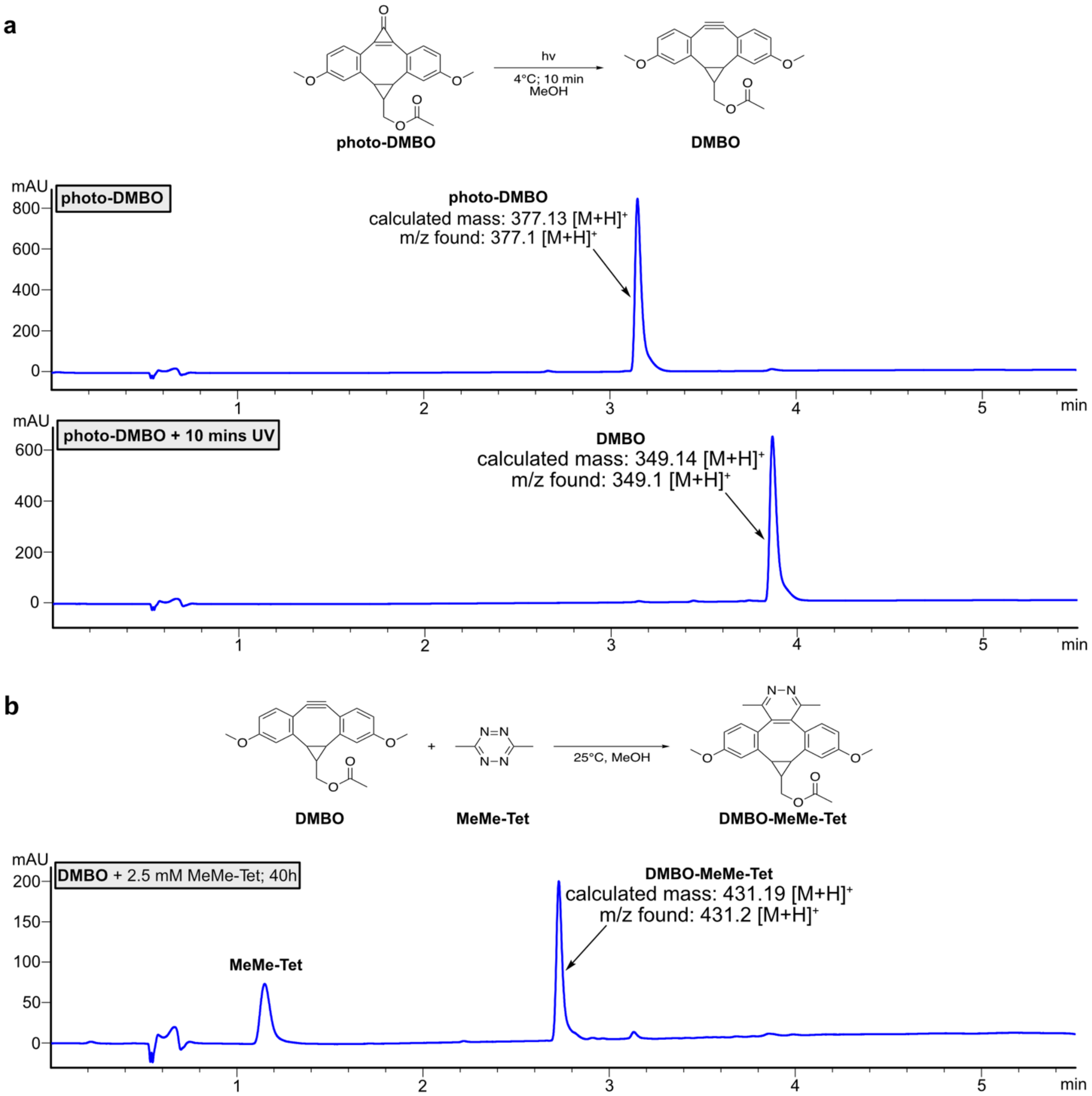

Supplementary Fig S1: LC-MS assay of **photo-DMBO**, **DMBO** and its reaction with **MeMe-Tet** (reaction conditions: concentration **DMBO**: 250 µM and concentration **MeMe-Tet**: 2.5 mM, in MeOH, 25 °C). a) Shows the photo-deprotection reaction of **photo-DMBO** to **DMBO** and the corresponding LC-MS absorption trace (254 nm) of **photo-DMBO** and **DMBO** after 10 minutes of irradiation with 365 nm UV-light. b) Depicts the reaction equation of **DMBO** and **MeMe-Tet** as well as the LC-MS absorption trace (254 nm) of the reaction after 40 hours showing full conversion of **DMBO** to the iEDDAC product **DMBO-MeMe-Tet**.

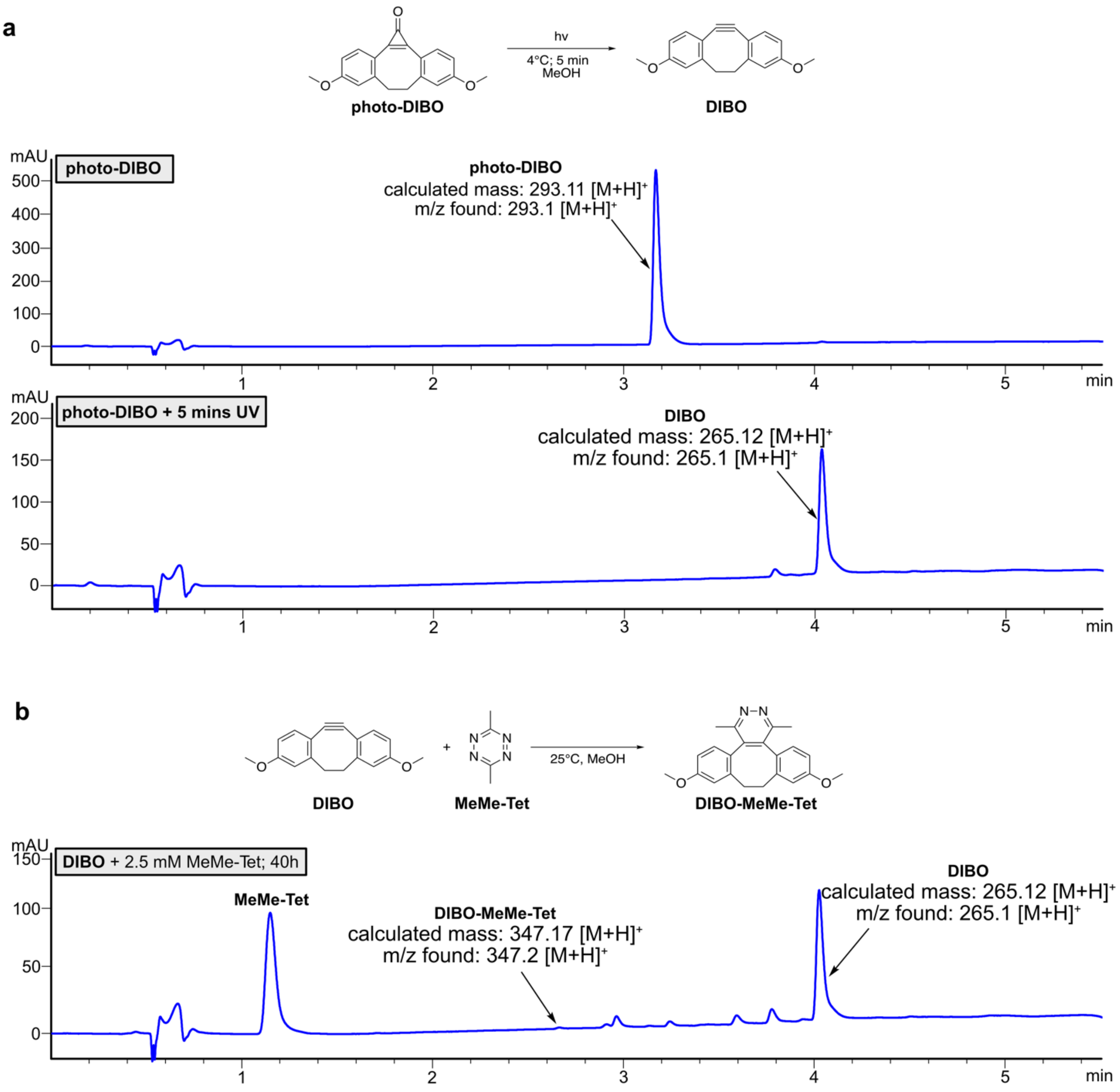

Supplementary Fig S2: LC-MS assay of **photo-DIBO**, **DIBO** and its reaction with **MeMe-Tet** (reaction conditions: concentration **DIBO**: 250 µM and concentration **MeMe-Tet**: 2.5 mM, in MeOH, 25 °C). a) Shows the photo-deprotection reaction of **photo-DIBO** to **DIBO** and the corresponding LC-MS absorption trace (254 nm) of **photo-DIBO** and **DIBO** after 5 minutes of irradiation with 365 nm UV-light. b) Depicts the reaction equation of **DIBO** and **MeMe-Tet** as well as the LC-MS absorption trace (254 nm) of the reaction after 40 hours showing nearly no conversion of **DIBO** to the iEDDAC product **DIBO-MeMe-Tet**.

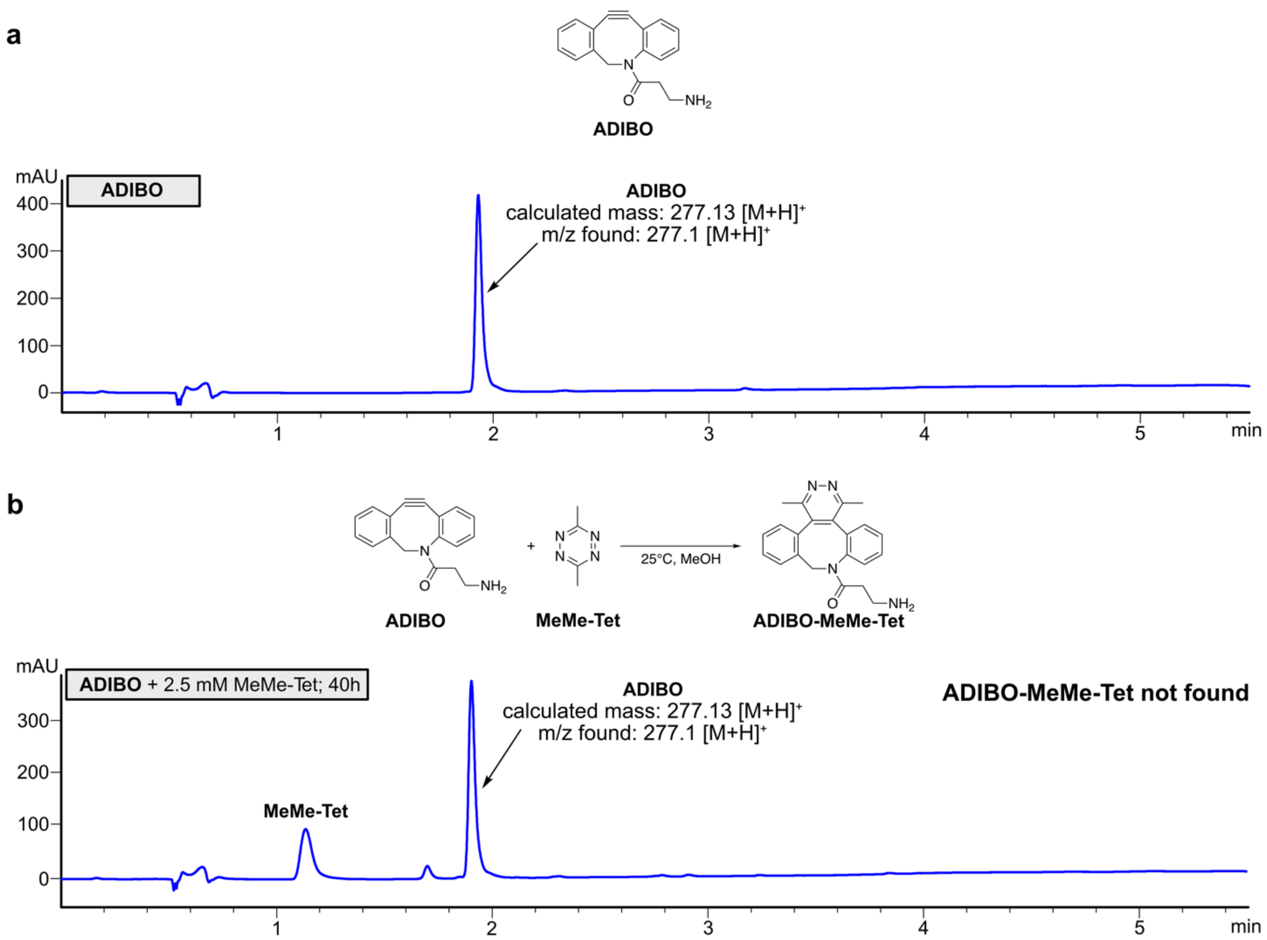

Supplementary Fig S3: LC-MS assay of **ADIBO** and its reaction with **MeMe-Tet** (ADIBO concentration: 250 µM and concentration **MeMe-Tet**: 2.5 mM, in MeOH, 25 °C). a) Shows the LC-MS absorption trace (254 nm) of **ADIBO**. b) Depicts the reaction equation of **ADIBO** and **MeMe-Tet** as well as the LC-MS absorption trace (254 nm) of the reaction after 40 hours showing no formation of the iEDDAC product **ADIBO-MeMe-Tet**.

a

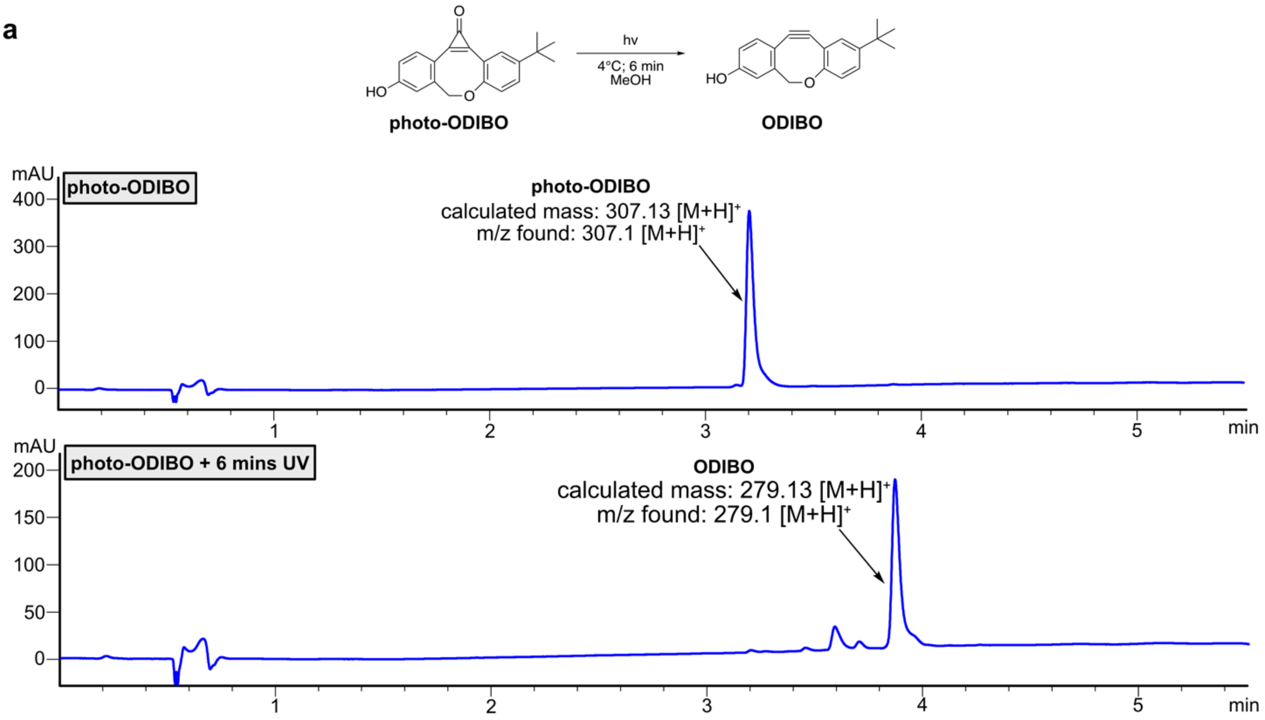

b

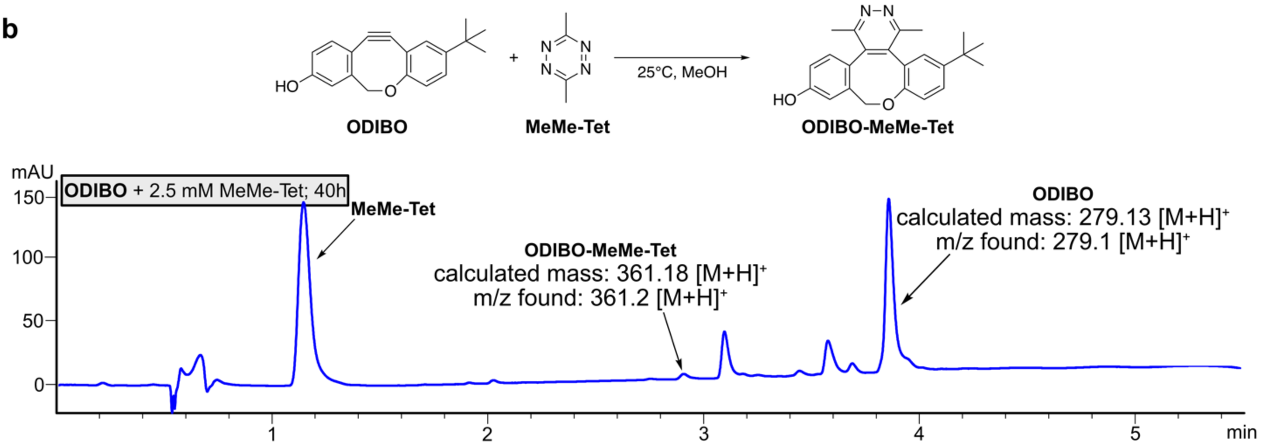

Supplementary Fig S4: LC-MS assay of **photo-ODIBO**, **ODIBO** and its reaction with **MeMe-Tet** (reaction conditions: concentration **ODIBO**: 250 µM and concentration **MeMe-Tet**: 2.5 mM, in MeOH, 25 °C). a) Shows the photo-deprotection reaction of **photo-ODIBO** to **ODIBO** and the corresponding LC-MS absorption trace (254 nm) of **photo-ODIBO** and **ODIBO** after 6 minutes of irradiation with 365 nm UV-light. b) Depicts the reaction equation of **ODIBO** and **MeMe-Tet** as well as the LC-MS absorption trace (254 nm) of the reaction after 40 hours showing nearly no conversion of **ODIBO** to the iEDDAC product **ODIBO-MeMe-Tet**.

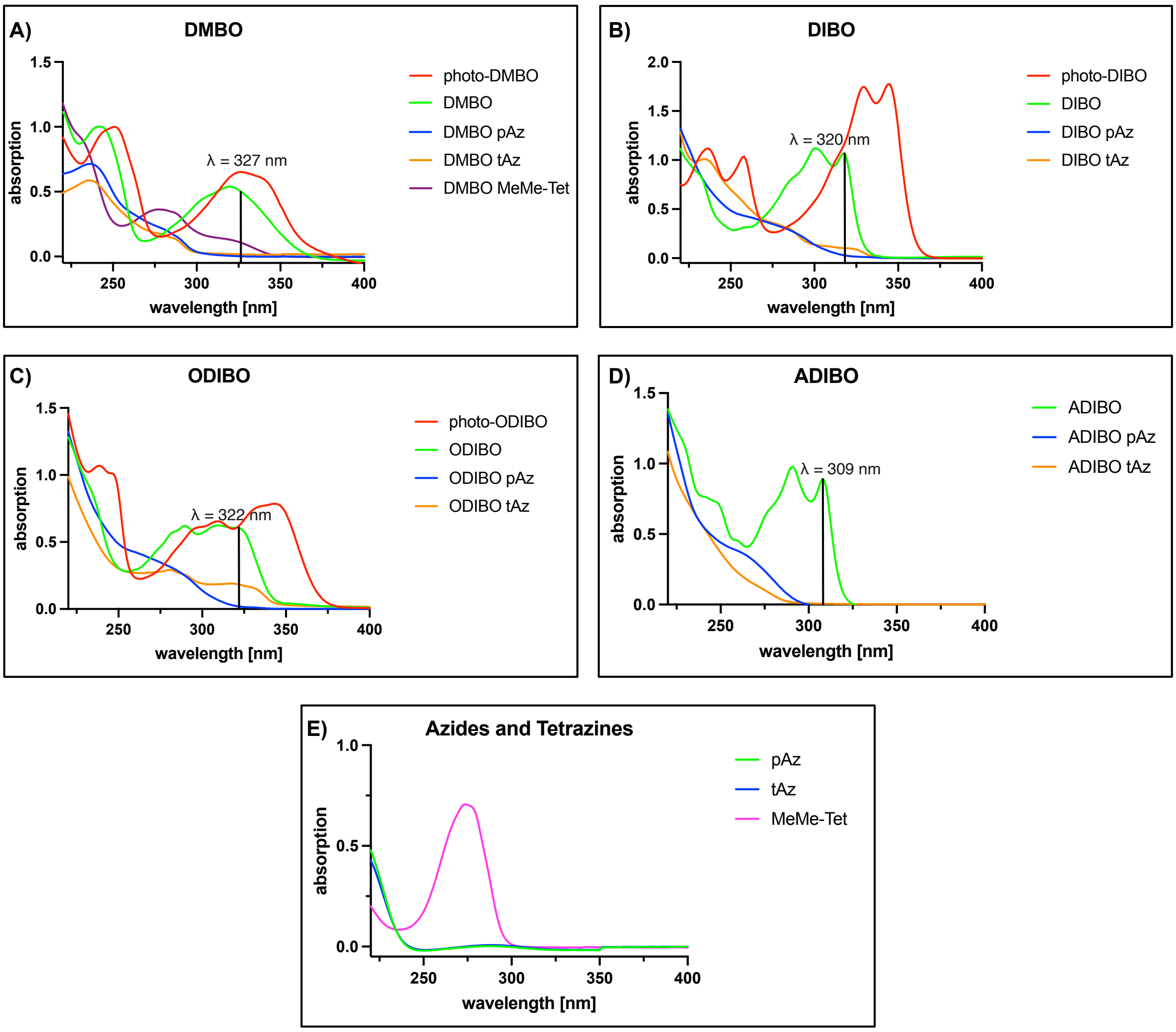

Supplementary Fig S5: UV-Vis spectra of 50 μM cyclooctynes (red and green curves) and the corresponding SPAAC or iEDDAC reaction products (blue, orange and purple curves). The black line indicates the wavelength at which kinetics were measured. A) UV-spectra of **DMBO** and reaction products in MeOH. B) UV-spectra of **DIBO** and reaction products in MeOH. C) UV-spectra of **ODIBO** and reaction products in MeOH. D) UV-spectra of **ADIBO** and reaction products in MeOH. E) UV-spectra of azides (**pAz** and **tAz**) and **MeMe-tet** each at 250 μM in MeOH.

A)

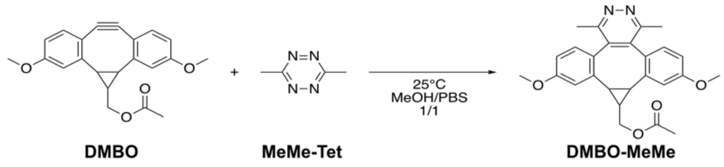

B)

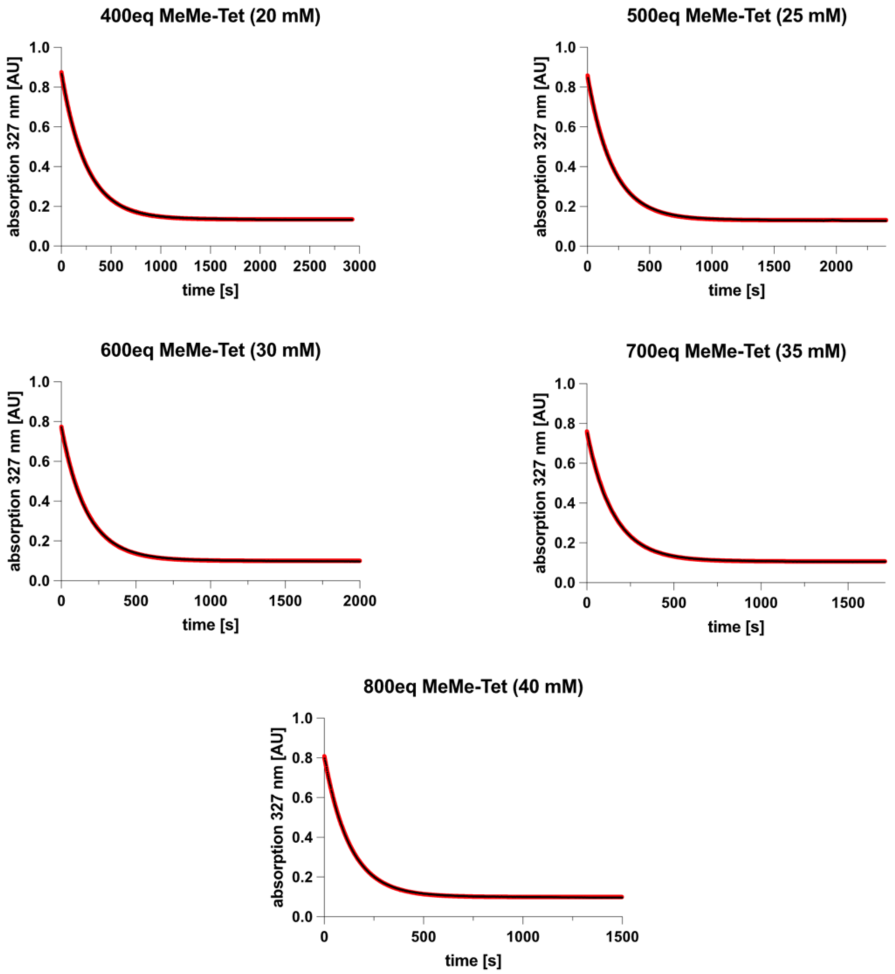

C)

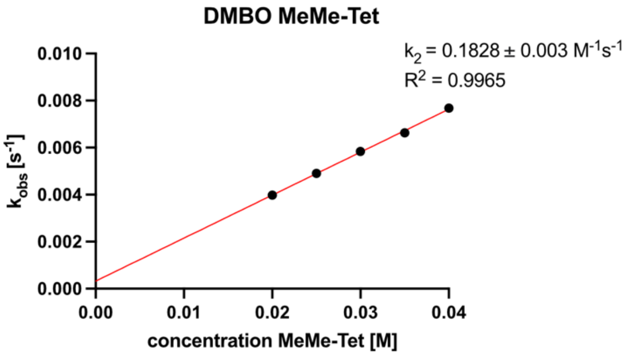

Supplementary Fig S6: Pseudo-first order kinetics between **DMBO** and **MeMe-Tet** in MeOH/PBS (1/1). a) Reaction equation between **DMBO** and **MeMe-Tet**. b) Shown is the decay of the characteristic **DMBO** absorption at 327 nm (data points are the black dots) in the presence of varying **MeMe-Tet** concentrations (20 mM – 40 mM) over time and the nonlinear regression calculated by prism software (fit curve is the red line). Note: only one of the triplicate runs is shown. c) Average k_{obs} from triplicate measurements are plotted against **MeMe-Tet** concentration and linear regression by prism software yielded the second-order rate constant k_2 of reaction between **DMBO** and **MeMe-Tet** (slope of line). Note: Error bars are not visible if they are smaller than the data point for the corresponding concentration of **MeMe-Tet**. **DMBO** concentration is 50 µM in MeOH/PBS (1/1).

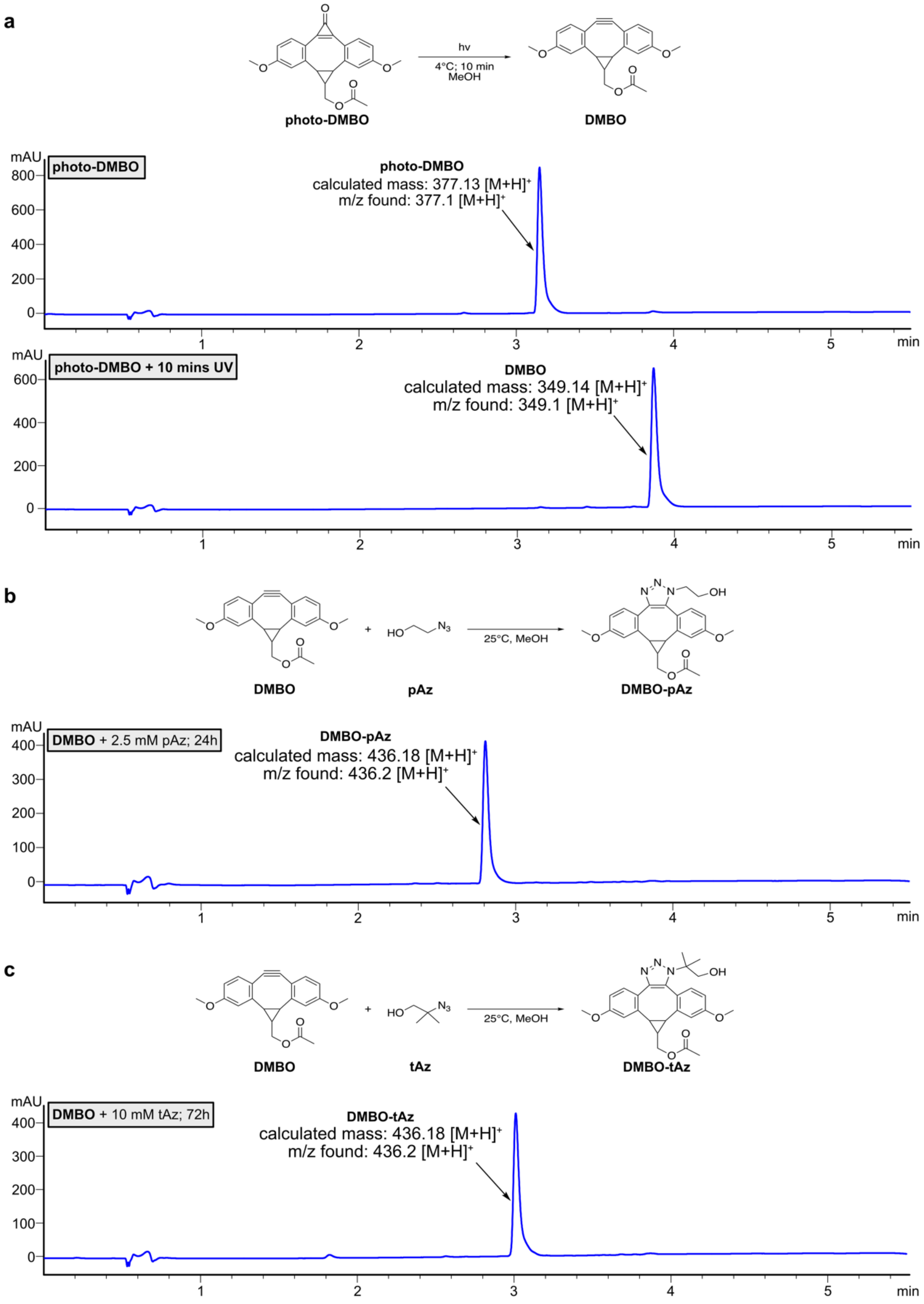

Supplementary Fig S7: LC-MS assay of **photo-DMBO**, **DMBO** and its reaction with primary or tertiary azides (cyclooctyne concentration is 250 µM for all experiments). a) Shows the photo-deprotection reaction of **photo-DMBO** to **DMBO** and the corresponding LC-MS absorption trace (254 nm) of **photo-DMBO** and **DMBO** after 10 minutes of irradiation with 365 nm UV-light. b) Depicts the reaction equation of **DMBO** and **pAz** as well as the LC-MS absorption trace (254 nm) of the reaction after 24 hours showing full conversion of **DMBO** to the SPAAC product **DMBO-pAz**. c) Depicts the reaction equation of **DMBO** and **tAz** as well as the LC-MS absorption trace (254 nm) of the reaction after 72 hours showing full conversion of **DMBO** to the SPAAC product **DMBO-tAz**.

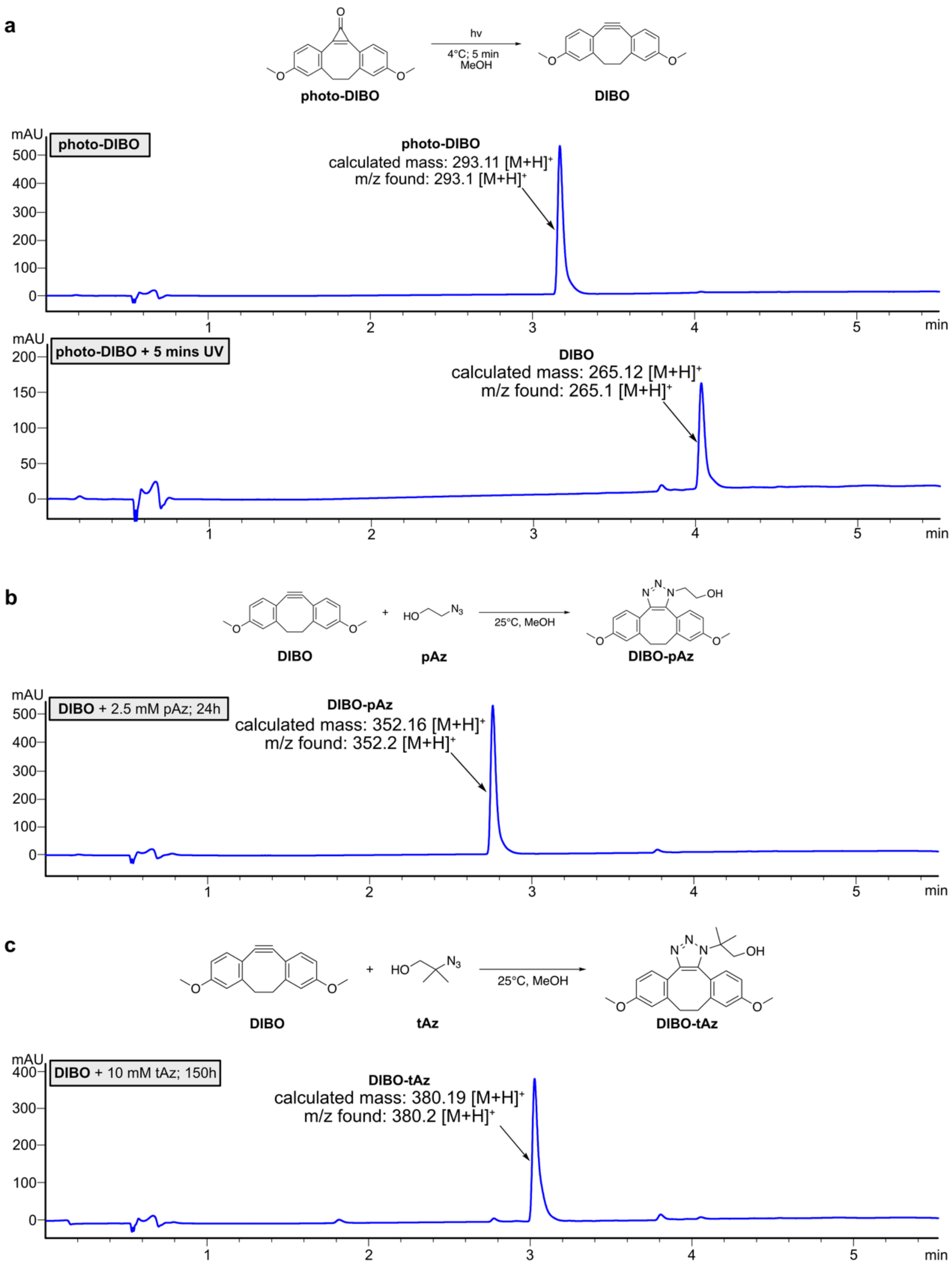

Supplementary Fig S8: LC-MS assay of **photo-DIBO**, **DIBO** and its reaction with primary or tertiary azides (cyclooctyne concentration is 250 µM for all experiments). a) Shows the photo-deprotection reaction of **photo-DIBO** to **DIBO** and the corresponding LC-MS absorption trace (254 nm) of **photo-DIBO** and **DIBO** after 5 minutes of irradiation with 365 nm UV-light. b) Depicts the reaction equation of **DIBO** and **pAz** as well as the LC-MS absorption trace (254 nm) of the reaction after 24 hours showing full conversion of **DIBO** to the SPAAC product **DIBO-pAz**. c) Depicts the reaction equation of **DIBO** and **tAz** as well as the LC-MS absorption trace (254 nm) of the reaction after 150 hours showing full conversion of **DIBO** to the SPAAC product **DIBO-tAz**.

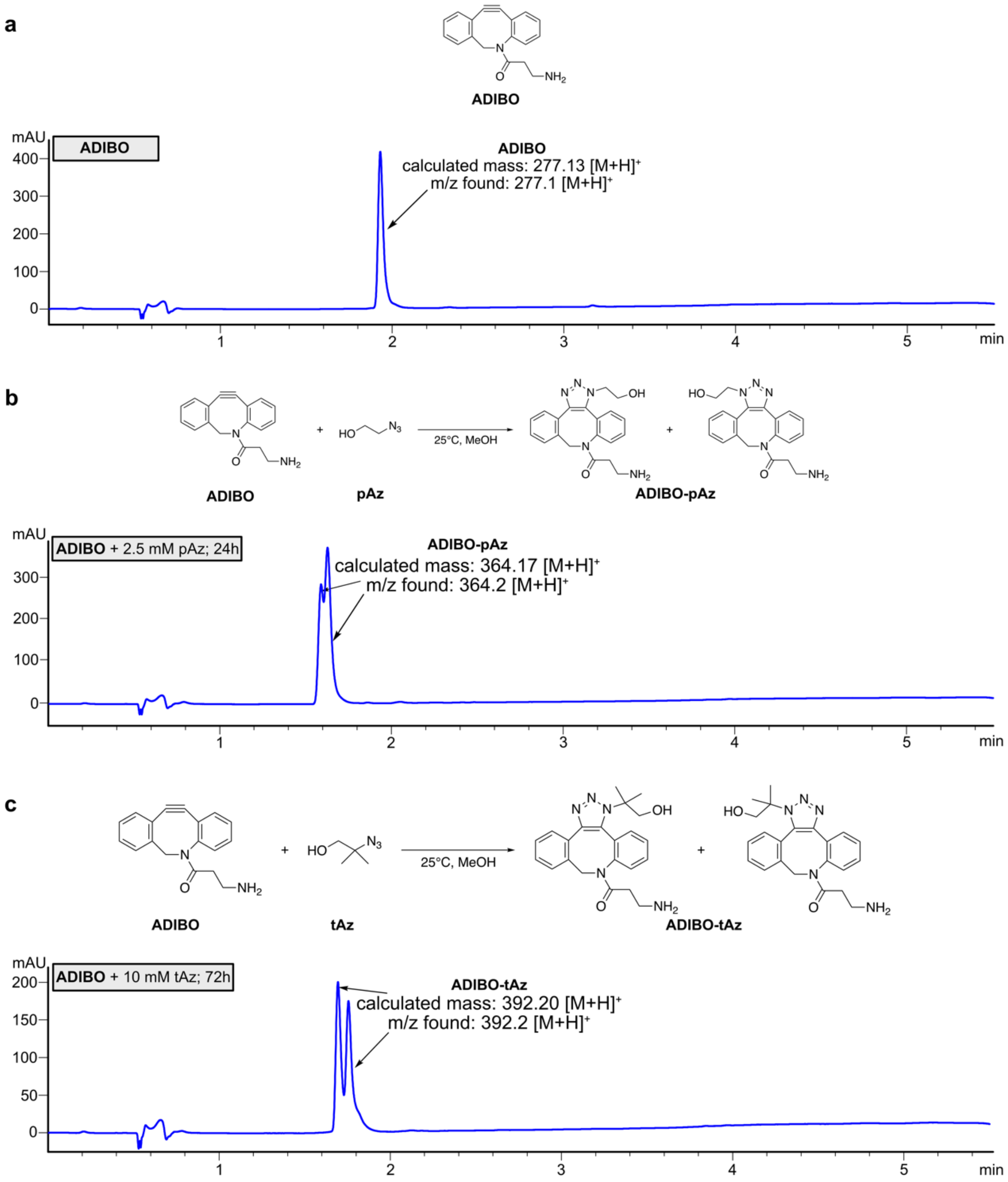

Supplementary Fig S9: LC-MS assay of **ADIBO** and its reaction with primary or tertiary azides (cyclooctyne concentration is 250 µM for all experiments). a) The structure of **ADIBO** and the corresponding LC-MS absorption trace (254 nm). b) Depicts the reaction equation of **ADIBO** and **pAz** as well as the LC-MS absorption trace (254 nm) of the reaction after 24 hours showing full conversion of **ADIBO** to the SPAAC product **ADIBO-pAz**. c) Depicts the reaction equation of **ADIBO** and **tAz** as well as the LC-MS absorption trace (254 nm) of the reaction after 72 hours showing full conversion of **ADIBO** to the SPAAC product **ADIBO-tAz**. For b) and c): Note that two peaks are observed since **ADIBO** is not symmetrical and yields two different reaction products (diastereomers).

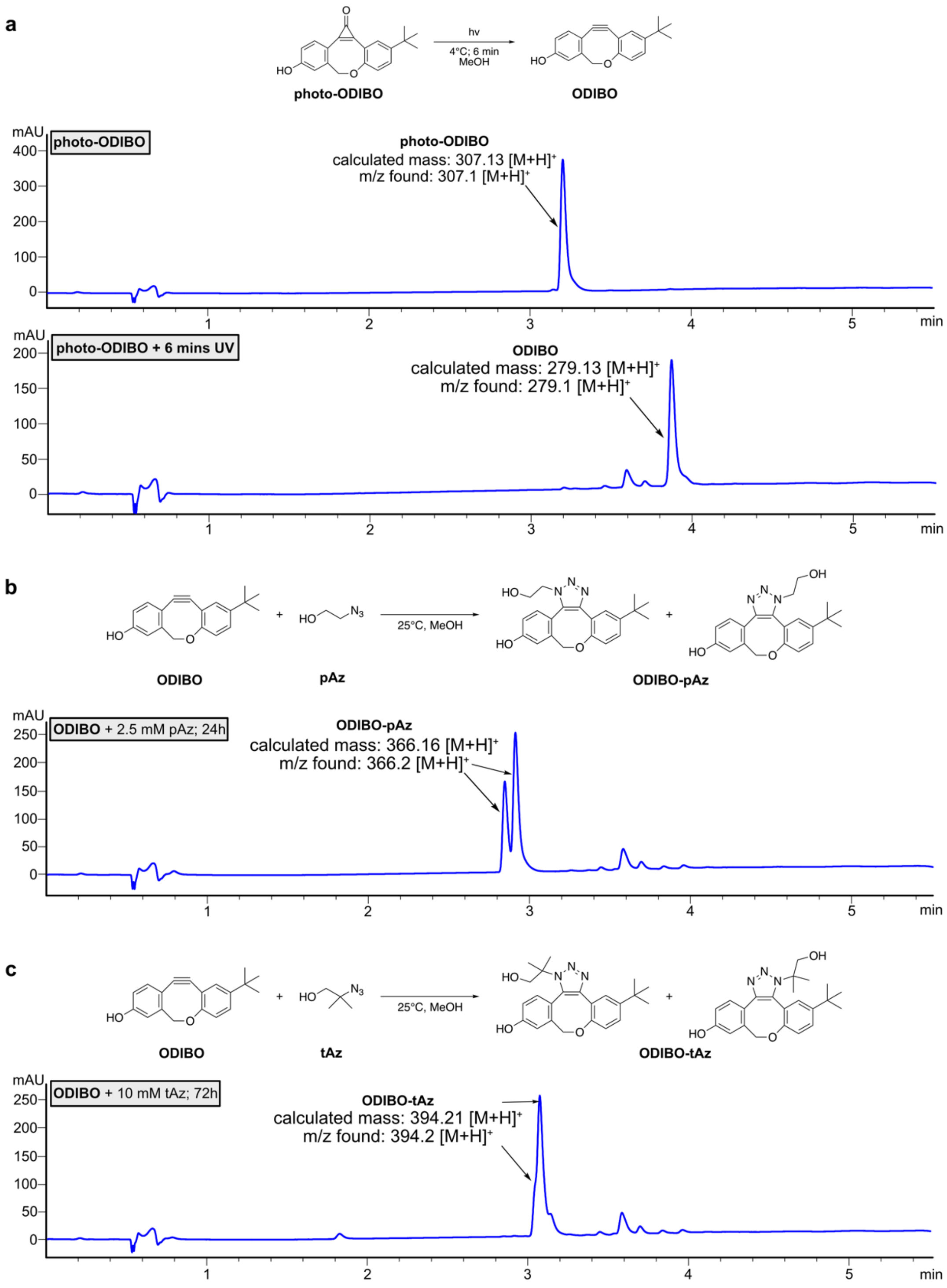

Supplementary Fig S10: LC-MS assay of **photo-ODIBO**, **ODIBO** and its reaction with primary or tertiary azides (cyclooctyne concentration is 250 µM for all experiments). a) Shows the photo-deprotection reaction of **photo-ODIBO** to **ODIBO** and the corresponding LC-MS absorption trace (254 nm) of **photo-ODIBO** and **ODIBO** after 6 minutes of irradiation with 365 nm UV-light. b) Depicts the reaction equation of **ODIBO** and **pAz** as well as the LC-MS absorption trace (254 nm) of the reaction after 24 hours showing full conversion of **ODIBO** to the SPAAC product **ODIBO-pAz**. c) Depicts the reaction equation of **ODIBO** and **tAz** as well as the LC-MS absorption trace (254 nm) of the reaction after 72 hours showing full conversion of **ODIBO** to the SPAAC product **ODIBO-tAz**. For b) and c): Note that two peaks are observed since **ODIBO** is not symmetrical and yields two different reaction products (diastereomers.

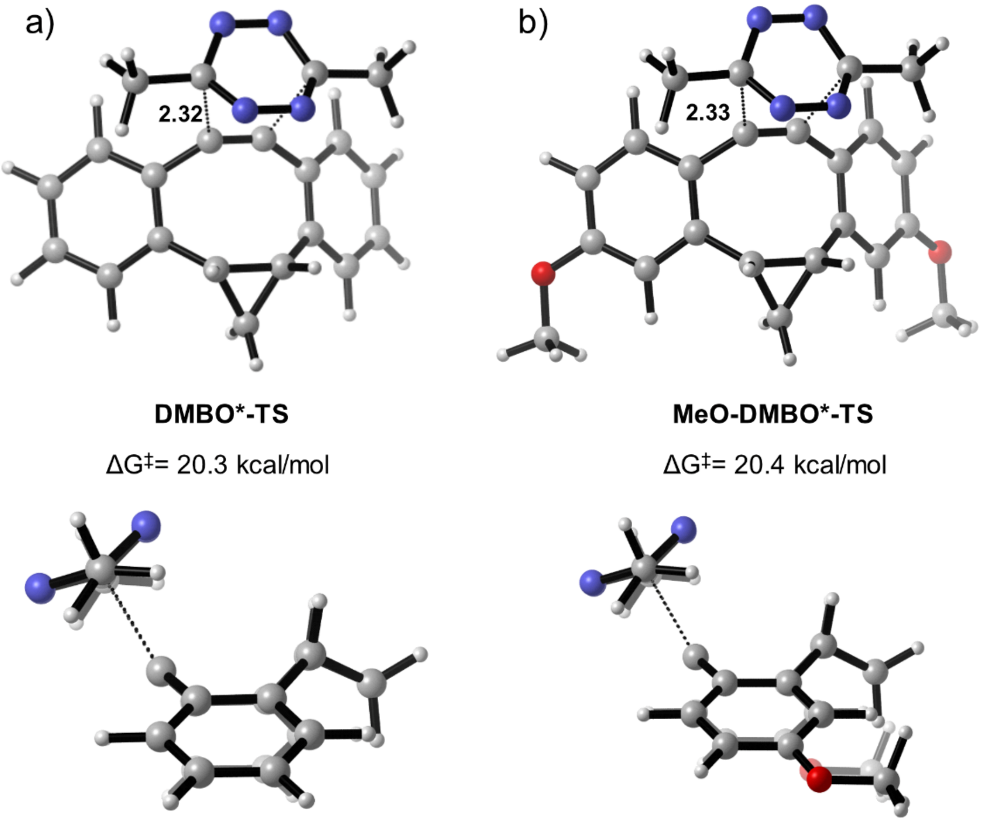

Supplementary Fig S11: Transition states for Diels-Alder reactions between **MeMeTet** and **DMBO*** or **MeO-DMBO*** highlighting that the methoxy groups of DMBO have little effect on barrier height and geometries. Calculated at the M06-2X-D3/6-311+G(d,p) SMD(water) level of theory.

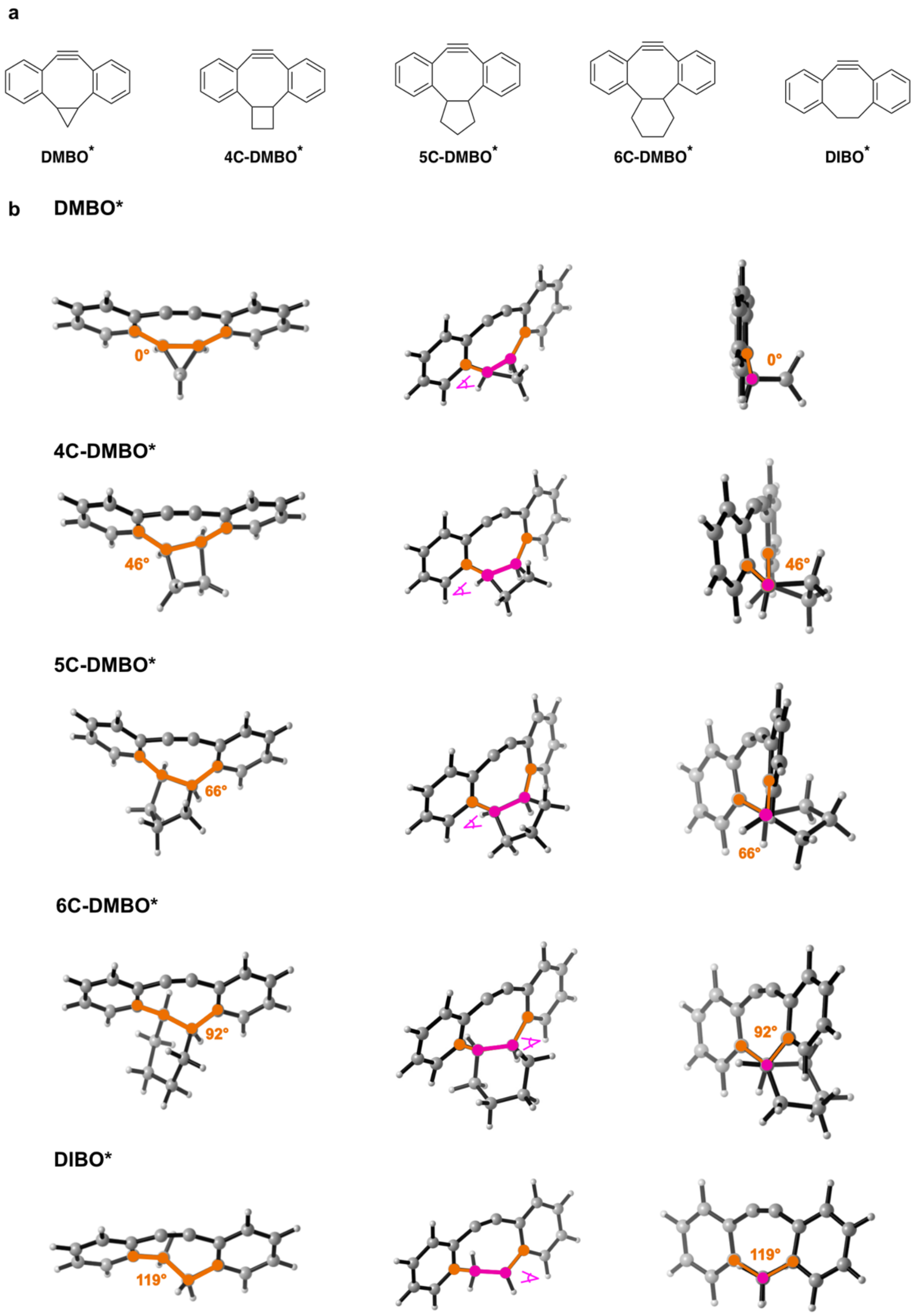

Supplementary Fig S12: Dibenzocyclooctynes fused with differently sized cycloalkanes and their effect on structure. a) Chemical structures of the dibenzoannulated cyclooctynes used in DFT calculations. b) DFT geometries of **DMBO***, **4C-**, **5C-**, **6C-DMBO*** and **DIBO*** calculated using M06-2X-D3/6-311+G(d,p) SMD(water). The dihedral angle of the C-4 backbone unit is highlighted in orange and absolute deviation from a planar 0° angle is given. Geometries are shown from three different angles. Looking along the pink-marked bond is used to the evaluate the structure of the cyclooctyne core and better visualization of the dihedral angle in the C-4 backbone unit.

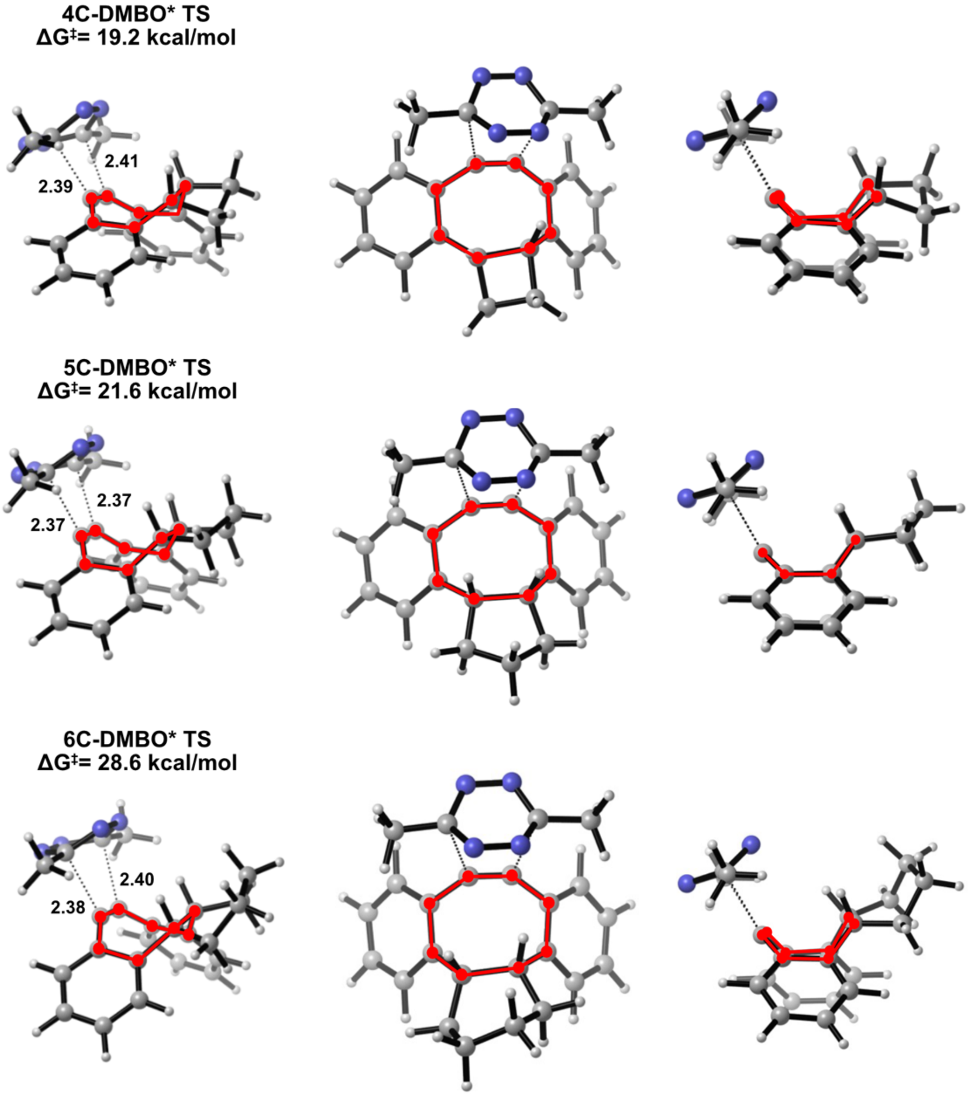

Supplementary Fig S13: DFT geometries of the transition states in the reaction between **4C-**, **5C-** or **6C-DMBO*** and **MeMe-Tet** calculated using M06-2X-D3/6-311+G(d,p) SMD(water). All cycloalkane fused dibenzocyclooctynes show exclusive *face* approach of **MeMe-Tet** and a tub-like structure in their transition state (marked red). Despite the similar geometry, larger cycloalkanes exhibit a higher activation barrier with **6C-DMBO*** only being 1.3 kcal/mol lower than unsubstituted **DIBO***. The transition sates are shown from three different angles and the cyclooctyne core is marked in red.

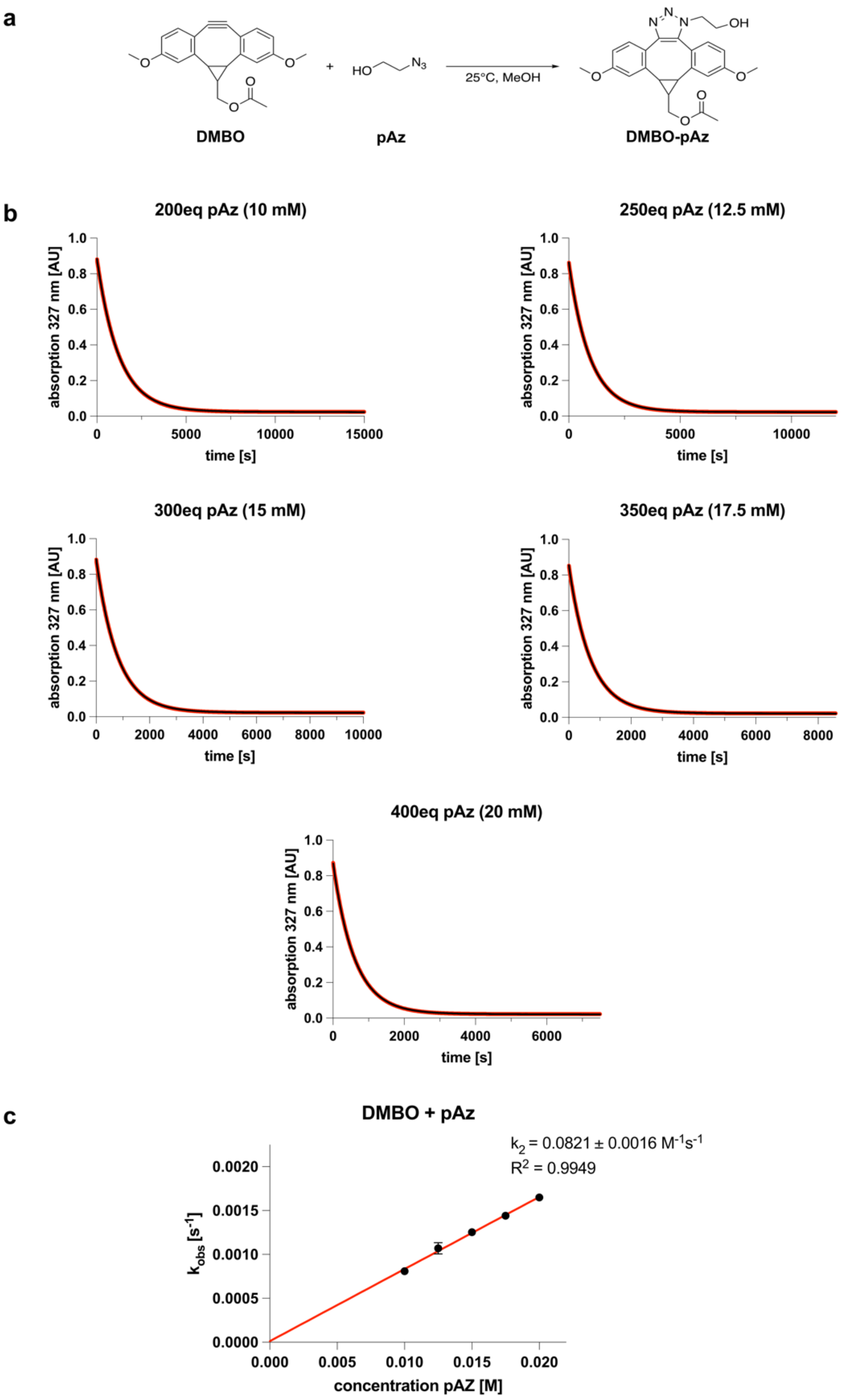

Supplementary Fig S14: Pseudo-first order kinetics between **DMBO** and **pAz**. a) Reaction equation between **DMBO** and primary azide (**pAz**). b) Shown is the decay of the characteristic **DMBO** absorption at 327 nm (data points are the black dots) in the presence of varying **pAz** concentrations (10 mM – 20 mM) over time and the nonlinear regression calculated by prism software (fit curve is the red line). Note: only one of the triplicate runs is shown. c) Average k_{obs} from triplicate measurements are plotted against **pAz** concentration and linear regression by prism software yielded the second-order rate constant k_2 between **DMBO** and **pAz** (slope of line). Note: Error bars for k_{obs} are not visible if they are smaller than the data point for the corresponding concentration of **pAz**. **DMBO** concentration is 50 µM in MeOH.

a

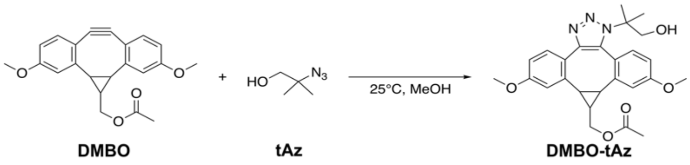

b

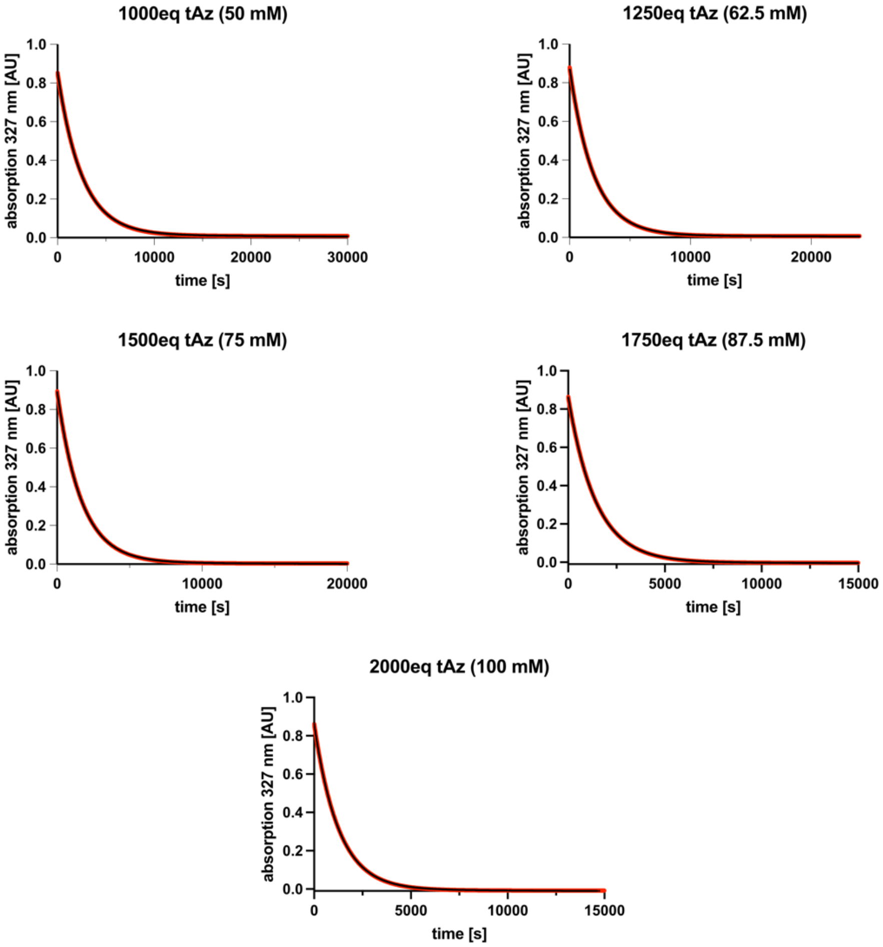

c

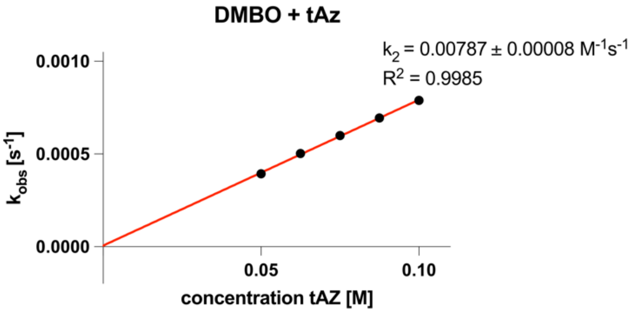

Supplementary Fig S15: Pseudo-first order kinetics between **DMBO** and **tAz**. a) Reaction equation between **DMBO** and tertiary azide (**tAz**). b) Shown is the decay of the characteristic **DMBO** absorption at 327 nm (data points are the black dots) in the presence of varying **tAz** concentrations (50 mM – 100 mM) over time and the nonlinear regression calculated by prism software (fit curve is the red line). Note: only one of the triplicate runs is shown. c) Average k_{obs} from triplicate measurements are plotted against **tAz** concentration and linear regression by prism software yielded the second-order rate constant k_2 between **DMBO** and **tAz** (slope of line). Note: Error bars for k_{obs} are not visible if they are smaller than the data point for the corresponding concentration of **tAz**. **DMBO** concentration is 50 μM in MeOH.

a

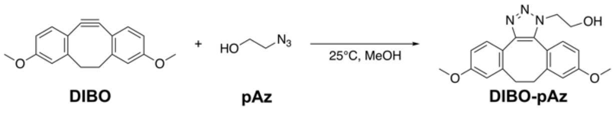

b

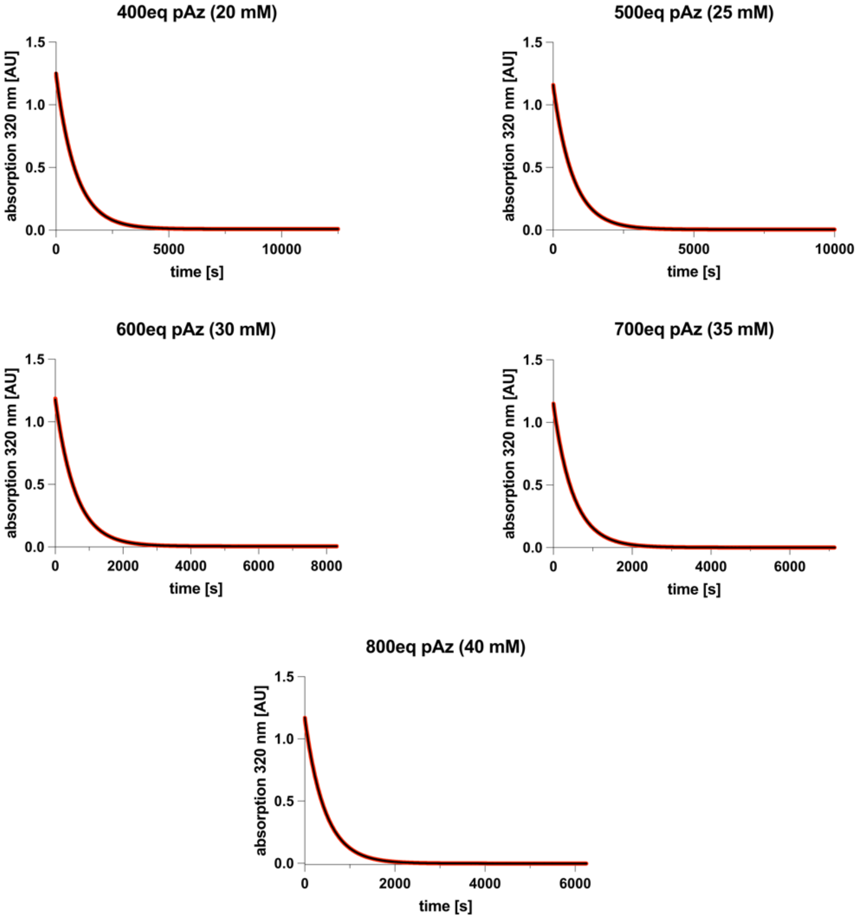

c

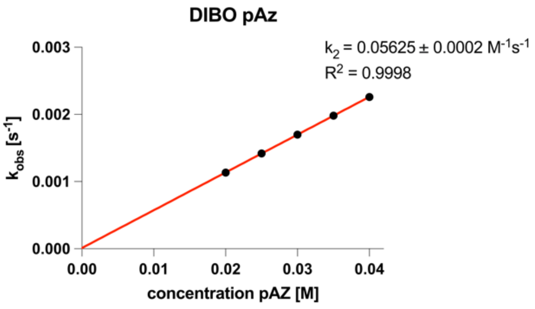

Supplementary Fig S16: Pseudo-first order kinetics between **DIBO** and **pAz**. a) Reaction equation between **DIBO** and primary azide (**pAz**). b) Shown is the decay of the characteristic **DIBO** absorption at 320 nm (data points are the black dots) in the presence of varying **pAz** concentrations (20 mM – 40 mM) over time and the nonlinear regression calculated by prism software (fit curve is the red line). Note: only one of the triplicate runs is shown. c) Average k_{obs} from triplicate measurements are plotted against **pAz** concentration and linear regression by prism software yielded the second-order rate constant k_2 between **DIBO** and **pAz** (slope of line). Note: Error bars for k_{obs} are not visible if they are smaller than the data point for the corresponding concentration of **pAz**. **DIBO** concentration is 50 µM in MeOH.

a

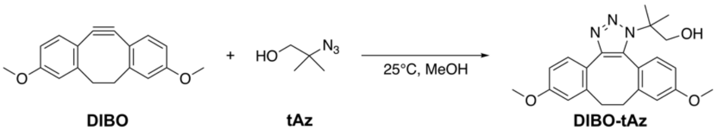

b

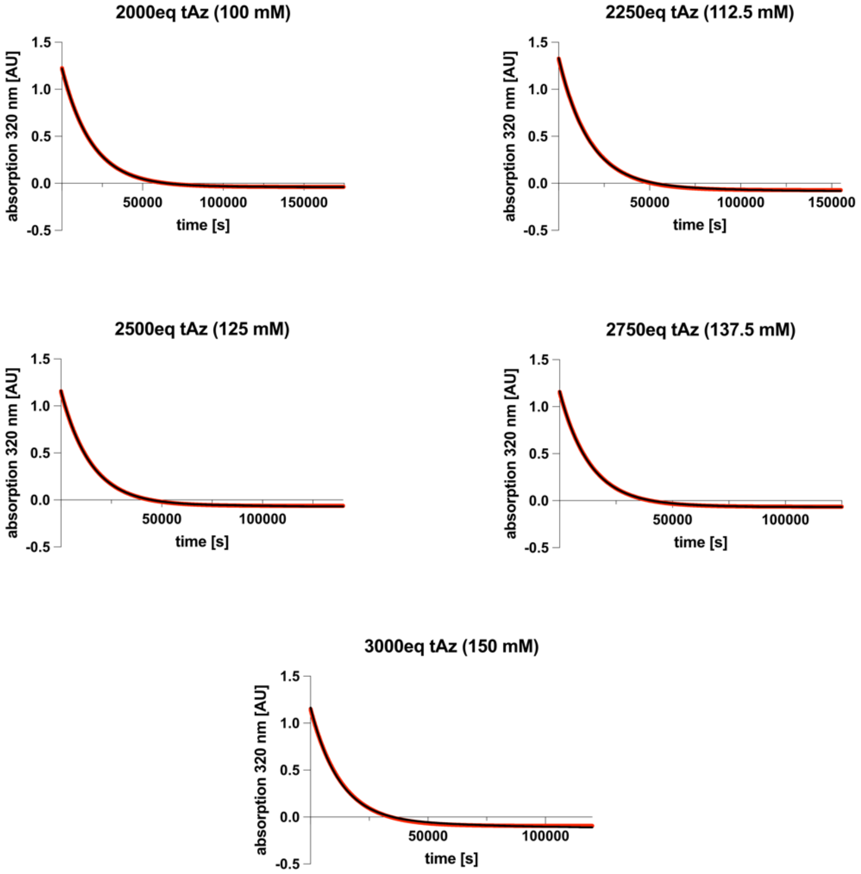

c

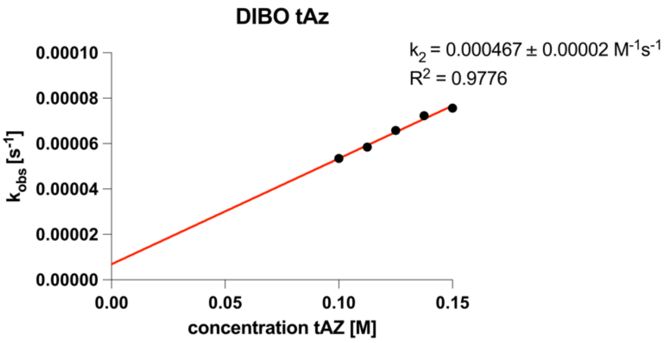

Supplementary Fig S17: Pseudo-first order kinetics between **DIBO** and **tAz**. a) Reaction equation between **DIBO** and tertiary azide (**tAz**). b) Shown is the decay of the characteristic **DIBO** absorption at 320 nm (data points are the black dots) in the presence of varying **tAz** concentrations (100 mM – 150 mM) over time and the nonlinear regression calculated by prism software (fit curve is the red line). Note: only one of the triplicate runs is shown. c) Average k_{obs} from triplicate measurements are plotted against **tAz** concentration and linear regression by prism software yielded the second-order rate constant k_2 between **DIBO** and **tAz** (slope of line). Note: Error bars for k_{obs} are not visible if they are smaller than the data point for the corresponding concentration of **tAz**. **DIBO** concentration is 50 µM in MeOH.

a

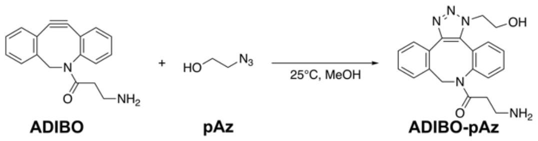

b

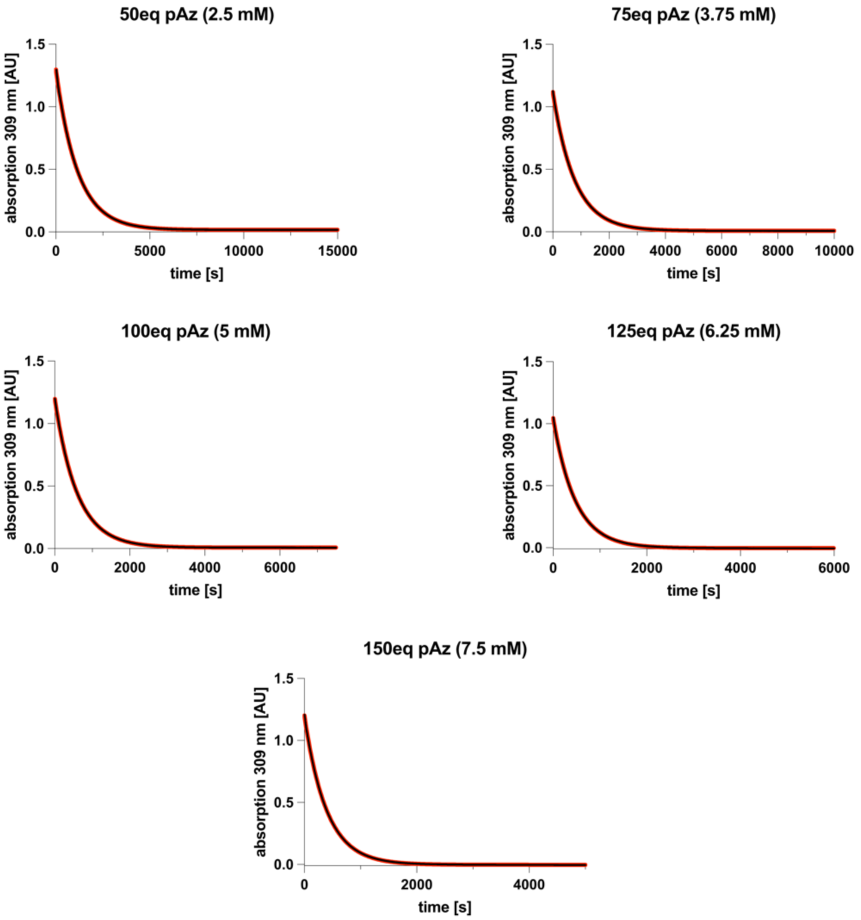

c

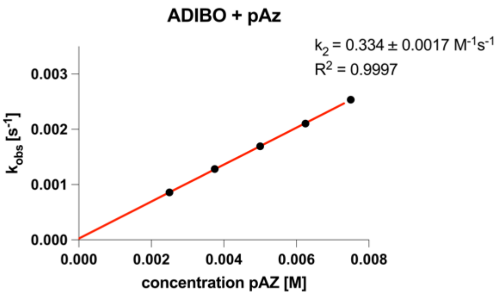

Supplementary Fig S18: Pseudo-first order kinetics between **ADIBO** and **pAz**. a) Reaction equation between **ADIBO** and primary azide (**pAz**). b) Shown is the decay of the characteristic **ADIBO** absorption at 309 nm (data points are the black dots) in the presence of varying **pAz** concentrations (2.5 mM – 7.5 mM) over time and the nonlinear regression calculated by prism software (fit curve is the red line). Note: only one of the triplicate runs is shown. c) Average k_{obs} from triplicate measurements are plotted against **pAz** concentration and linear regression by prism software yielded the second-order rate constant k_2 between **ADIBO** and **pAz** (slope of line). Note: Error bars for k_{obs} are not visible if they are smaller than the data point for the corresponding concentration of **pAz**. **ADIBO** concentration is 50 µM in MeOH.

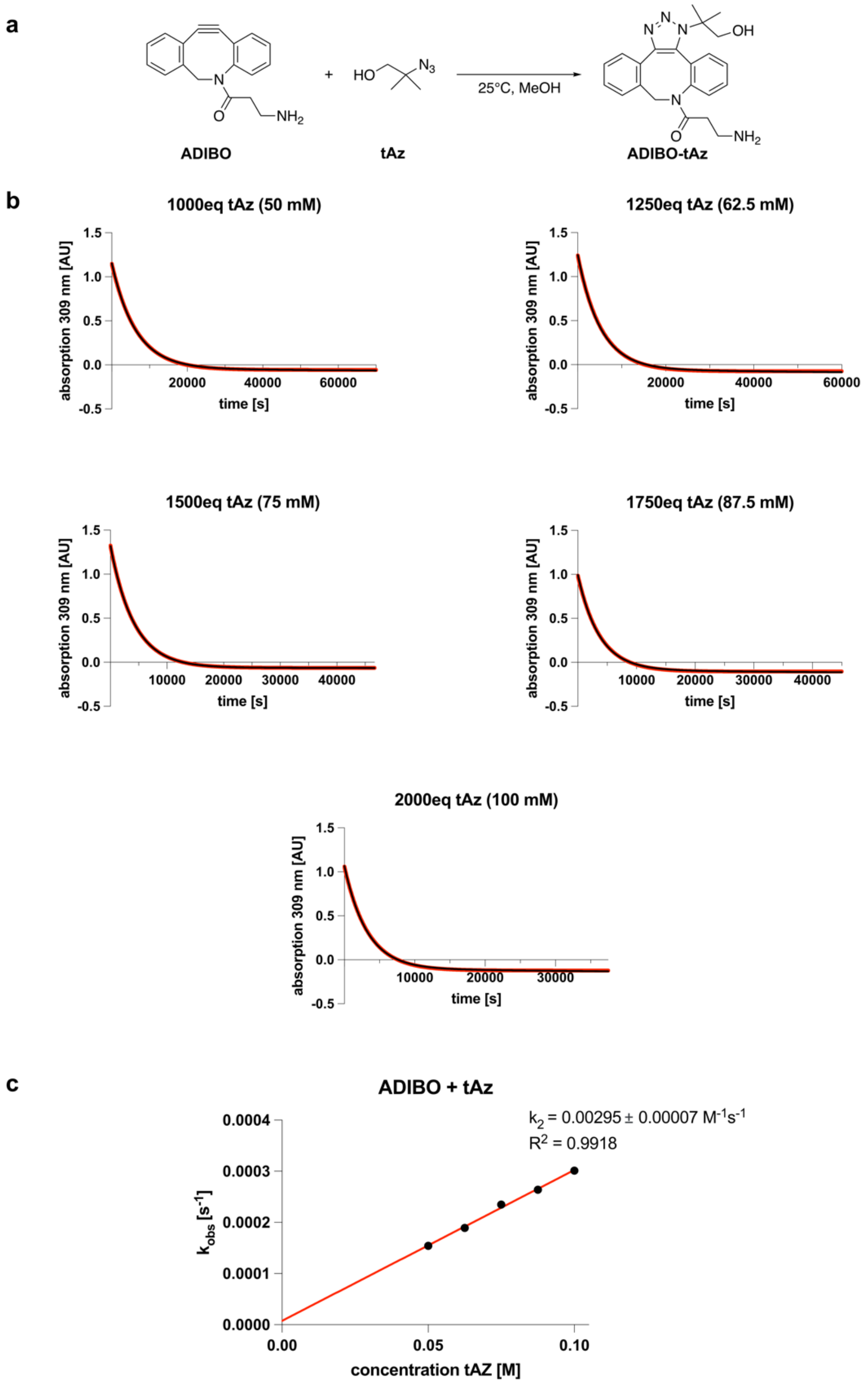

Supplementary Fig S19: Pseudo-first order kinetics between **ADIBO** and **tAz**. a) Reaction equation between **ADIBO** and tertiary azide (**tAz**). b) Shown is the decay of the characteristic **ADIBO** absorption at 309 nm (data points are the black dots) in the presence of varying **tAz** concentrations (50 mM – 100 mM) over time and the nonlinear regression calculated by prism software (fit curve is the red line). Note: only one of the triplicate runs is shown. c) Average k_{obs} from triplicate measurements are plotted against **tAz** concentration and linear regression by prism software yielded the second-order rate constant k_2 between **ADIBO** and **tAz** (slope of line). Note: Error bars for k_{obs} are not visible if they are smaller than the data point for the corresponding concentration of **tAz**. **ADIBO** concentration is 50 µM in MeOH.

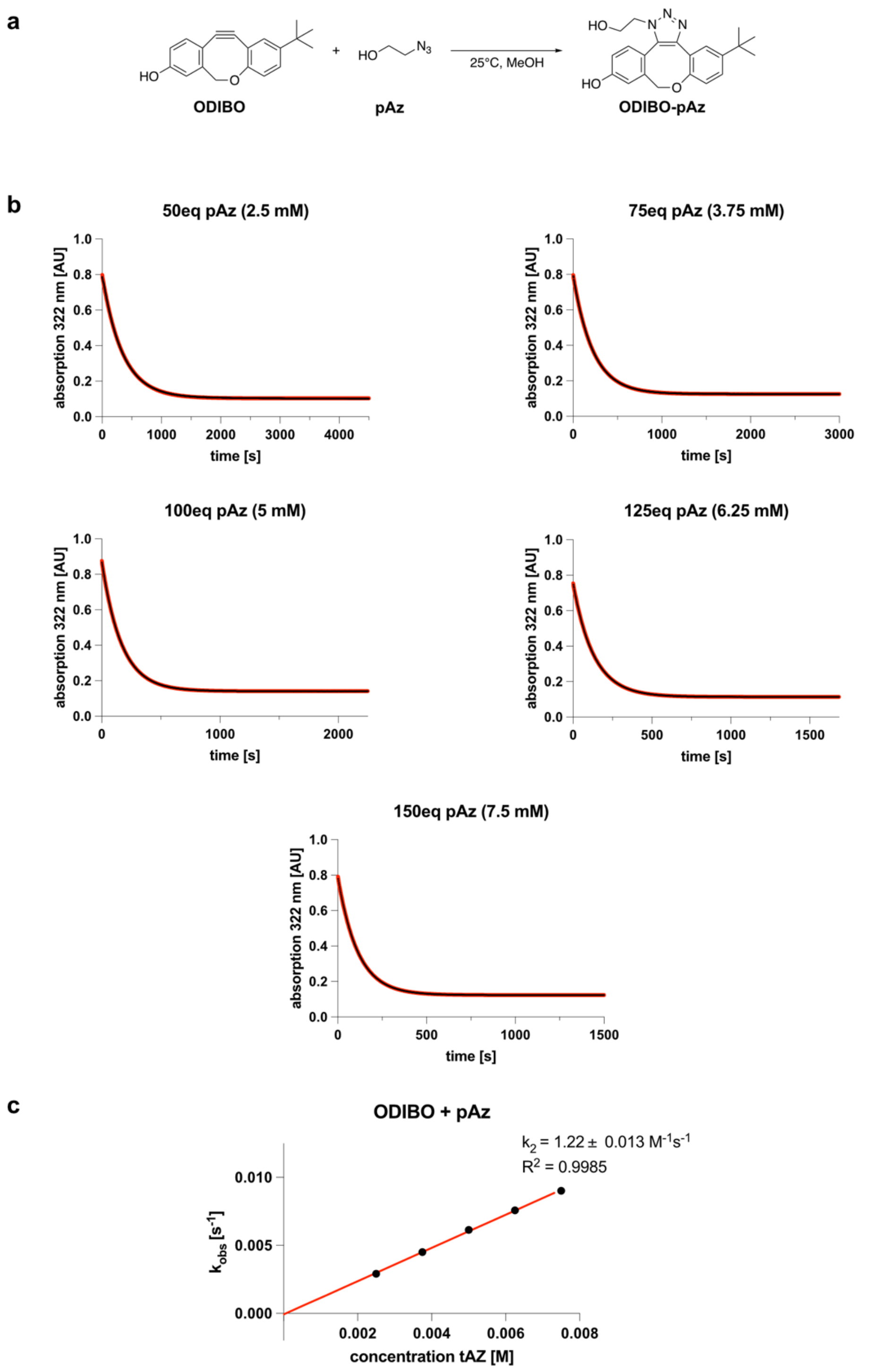

Supplementary Fig S20: Pseudo-first order kinetics between **ODIBO** and **pAz**. a) Reaction equation between **ODIBO** and primary azide (**pAz**). b) Shown is the decay of the characteristic **ODIBO** absorption at 322 nm (data points are the black dots) in the presence of varying **pAz** concentrations (2.5 mM – 7.5 mM) over time and the nonlinear regression calculated by prism software (fit curve is the red line). Note: only one of the triplicate runs is shown. c) Average k_{obs} from triplicate measurements are plotted against **pAz** concentration and linear regression by prism software yielded the second-order rate constant k_2 between **ODIBO** and **pAz** (slope of line). Note: Error bars for k_{obs} are not visible if they are smaller than the data point for the corresponding concentration of **pAz**. **ODIBO** concentration is 50 μM in MeOH.

a

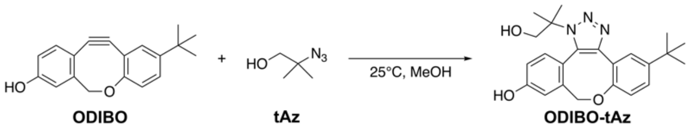

b

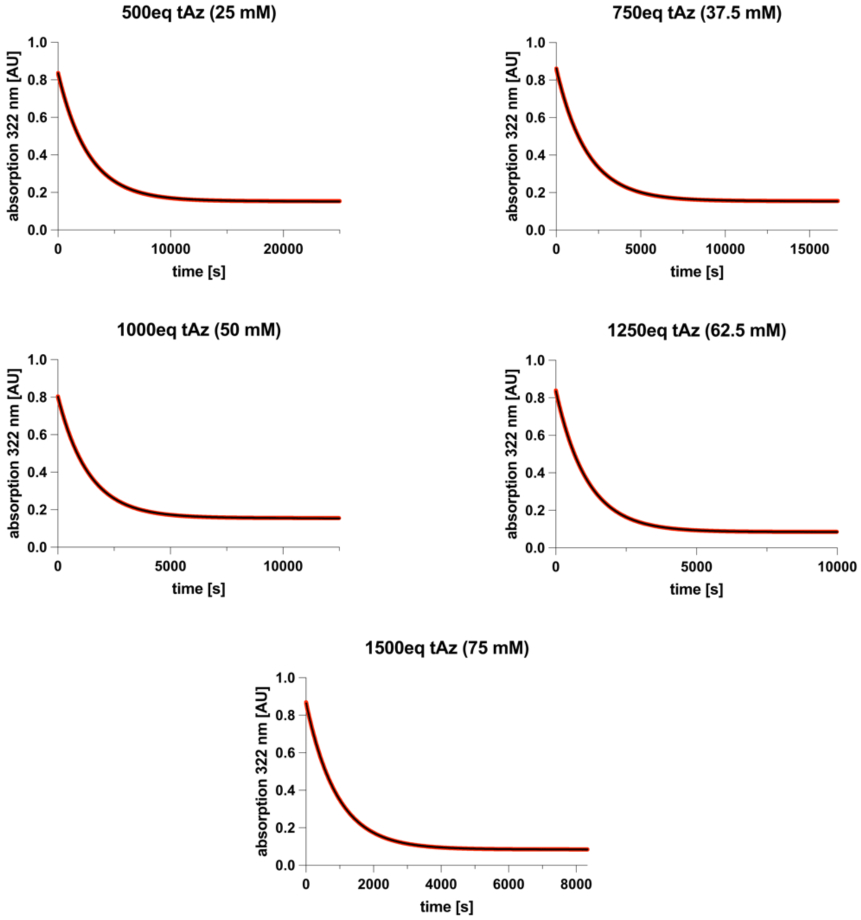

c

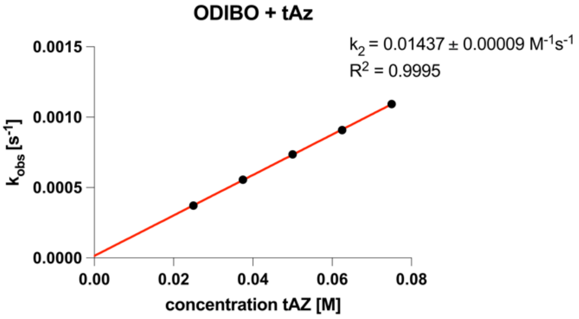

Supplementary Fig S21: Pseudo-first order kinetics between **ODIBO** and **tAz**. a) Reaction equation between **ODIBO** and tertiary azide (**tAz**). b) Shown is the decay of the characteristic **ODIBO** absorption at 322 nm (data points are the black dots) in the presence of varying **tAz** concentrations (25 mM – 75 mM) over time and the nonlinear regression calculated by prism software (fit curve is the red line). Note: only one of the triplicate runs is shown. c) Average k_{obs} from triplicate measurements are plotted against tAz concentration and linear regression by prism software yielded the second-order rate constant k_2 between **ODIBO** and **tAz** (slope of line). Note: Error bars for k_{obs} are not visible if they are smaller than the data point for the corresponding concentration of **tAz**. **ODIBO** concentration is 50μM in MeOH.

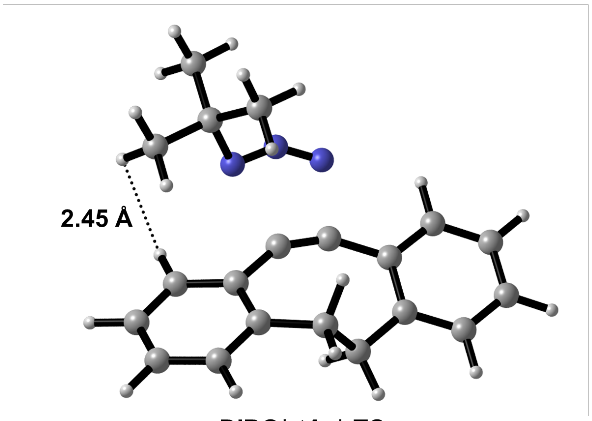

DIBO*-tAz*-TS

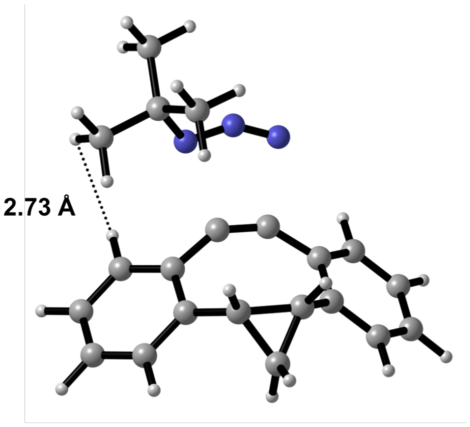

DMBO*-tAz*-TS

Supplementary Fig S22: Transition states for SPAAC reactions between **DIBO*** and **tAz*** and **DMBO*** and **tAz***. Shortest H–H distances in the **DIBO*-tAz*** and **DMBO*-tAz*** transition states are shown and indicated by dashed lines. Calculated at the M06-2X-D3/6-311+G(d,p) SMD(methanol) level of theory.

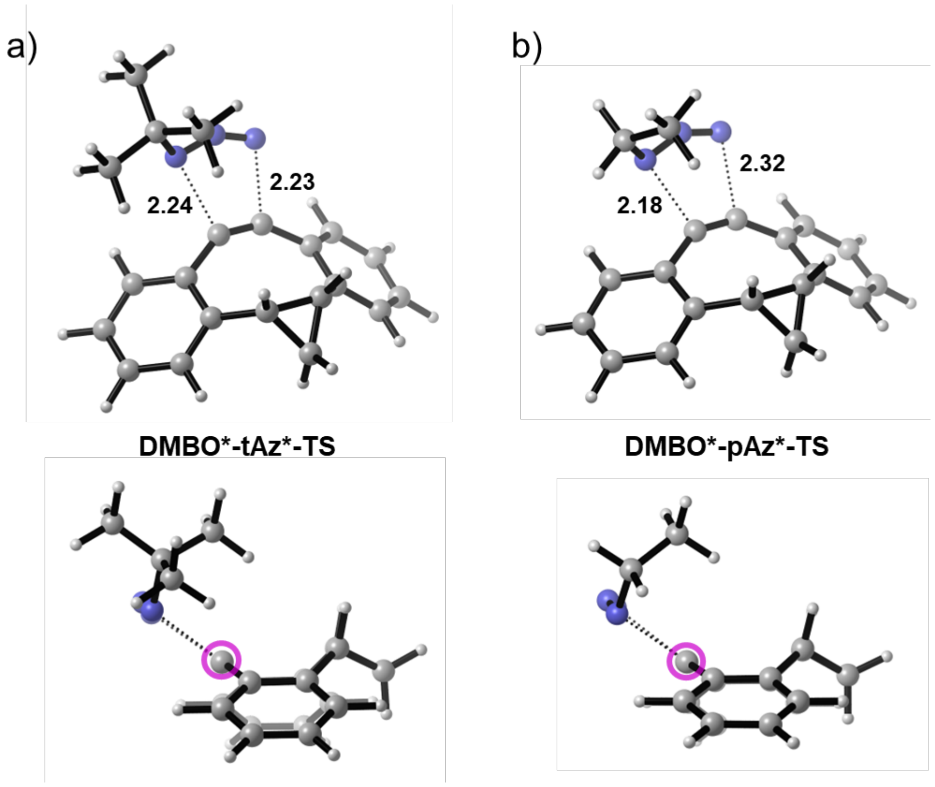

Supplementary Fig S23: Transition states for SPAAC reactions between **DMBO*** and **tAz*** or **pAz*** highlighting the reduced steric demand for the primary azide as evident by the longer forming bond length on the alkylated nitrogen. Calculated at the M06-2X-D3/6-311+G(d,p) SMD(methanol) level of theory.

References

1. F. Friscourt, C. J. Fahrni and G.-J. Boons, *J. Am. Chem. Soc.*, 2012, **134**, 18809-18815.
2. C. D. McNitt and V. V. Popik, *Org. Biomol. Chem.*, 2012, **10**, 8200-8202.
3. E. D. Goddard-Borger and R. V. Stick, *Org Lett*, 2007, **9**, 3797-3800.
4. M. Frisch, G. Trucks, H. Schlegel, G. Scuseria, M. Robb, J. Cheeseman, G. Scalmani, V. Barone, B. Mennucci and G. Petersson, *Gaussian 16 Revision A. 03, Gaussian Inc. Wallingford CT*, 2009.
5. Y. Zhao and D. G. Truhlar, *Theor. Chem. Acc.*, 2007, **120**, 215-241.
6. R. Krishnan, J. S. Binkley, R. Seeger and J. A. Pople, *J. Chem. Phys.*, 1980, **72**, 650-654.
7. S. Grimme, J. Antony, S. Ehrlich and H. Krieg, *J. Chem. Phys.*, 2010, **132**, 154104.
8. L. Goerigk, A. Hansen, C. Bauer, S. Ehrlich, A. Najibi and S. Grimme, *Physical Chemistry Chemical Physics*, 2017, **19**, 32184-32215.
9. A. V. Marenich, C. J. Cramer and D. G. Truhlar, *J. Phys. Chem. B*, 2009, **113**, 6378-6396.
10. P. Pracht, F. Bohle and S. Grimme, *Phys. Chem. Chem. Phys.*, 2020, **22**, 7169-7192.
11. C. Bannwarth, S. Ehlert and S. Grimme, *J. Chem. Theory. Comput.*, 2019, **15**, 1652-1671.
12. G. Luchini, J. V. Alegre-Requena, I. Funes-Ardoiz and R. S. Paton, *F1000Research*, 2020, **9**.
13. W. J. Zeist, C. F. Guerra and F. M. Bickelhaupt, *J. Comput. Chem.*, 2008, **29**, 312-315.
14. G. te Velde, F. M. Bickelhaupt, E. J. Baerends, C. Fonseca Guerra, S. J. A. van Gisbergen, J. G. Snijders and T. Ziegler, *J. Comput. Chem.*, 2001, **22**, 931-967.
15. C. Fonseca Guerra, J. G. Snijders, G. te Velde and E. J. Baerends, *Theor. Chem. Acc.*, 1998, **99**, 391-403.
16. , *ADF2018, SCM, Theoretical Chemistry, Vrije Universiteit, Amsterdam, The Netherlands,* http://www.scm.com.
17. E. Van Lenthe and E. J. Baerends, *J. Comput. Chem.*, 2003, **24**, 1142-1156.
18. D. Svatunek and K. N. Houk, *J. Comput. Chem.*, 2019, **40**, 2509-2515.

NMR Spectra

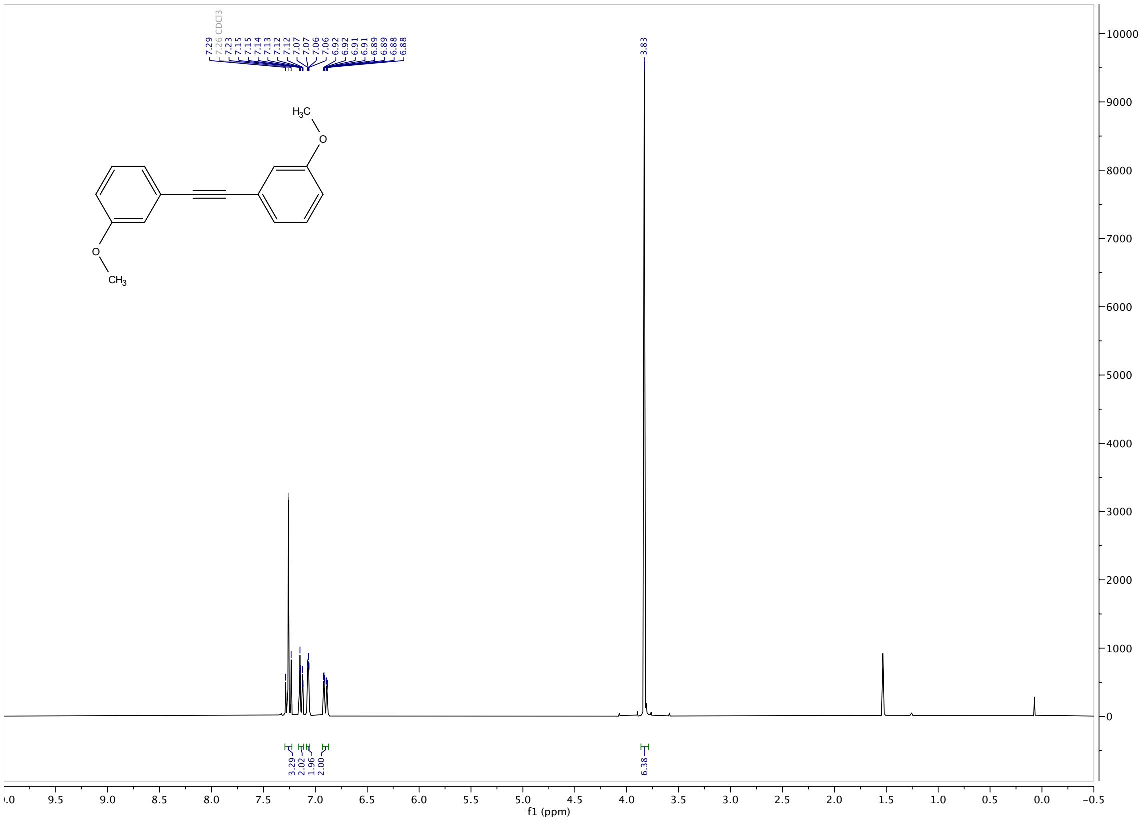

NMR-spectrum 1: ^{1}H-spectrum of **1**.

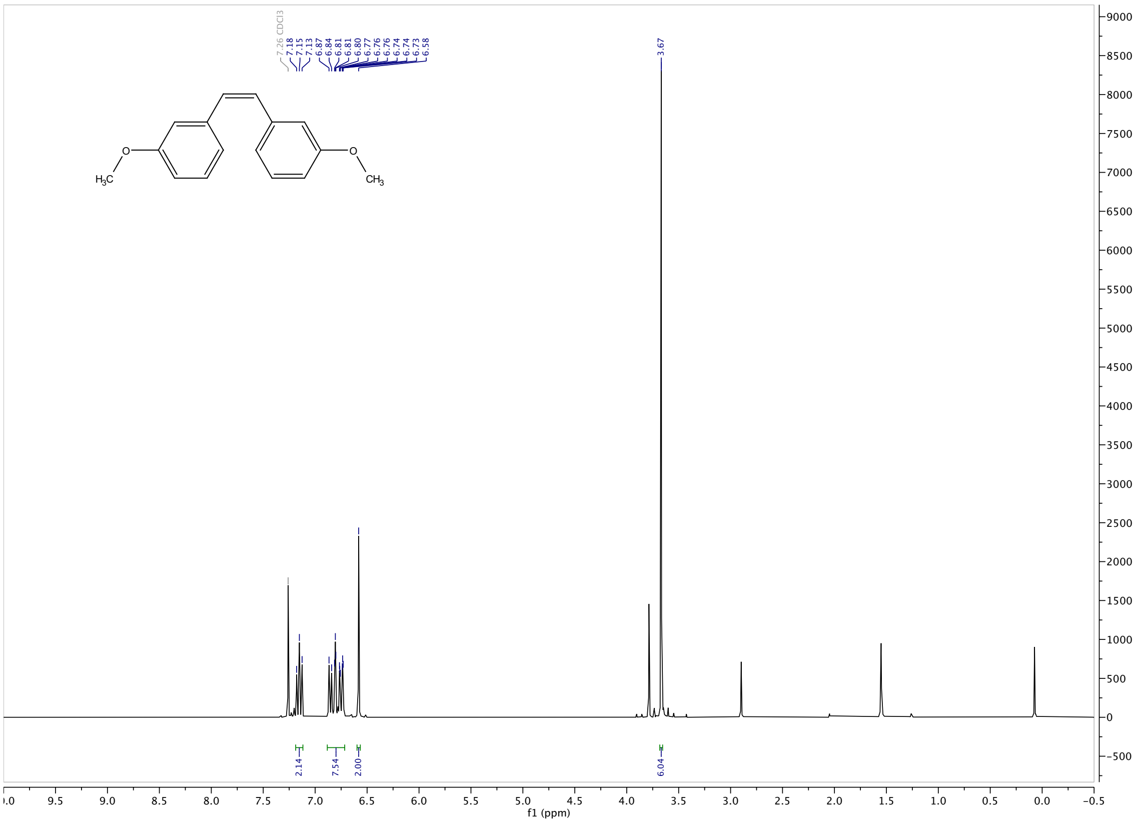

NMR-spectrum 2: ^{1}H-spectrum of **2**.

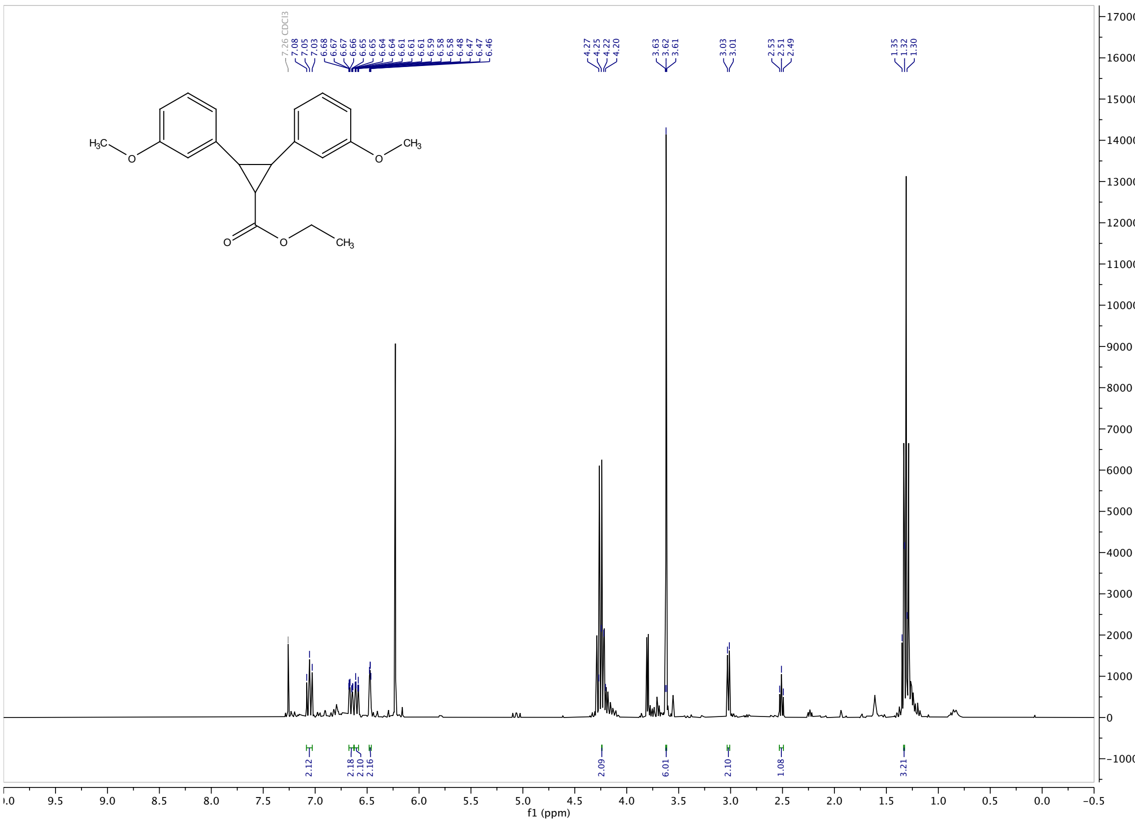

NMR-spectrum 3: ^{1}H-spectrum of **3**.

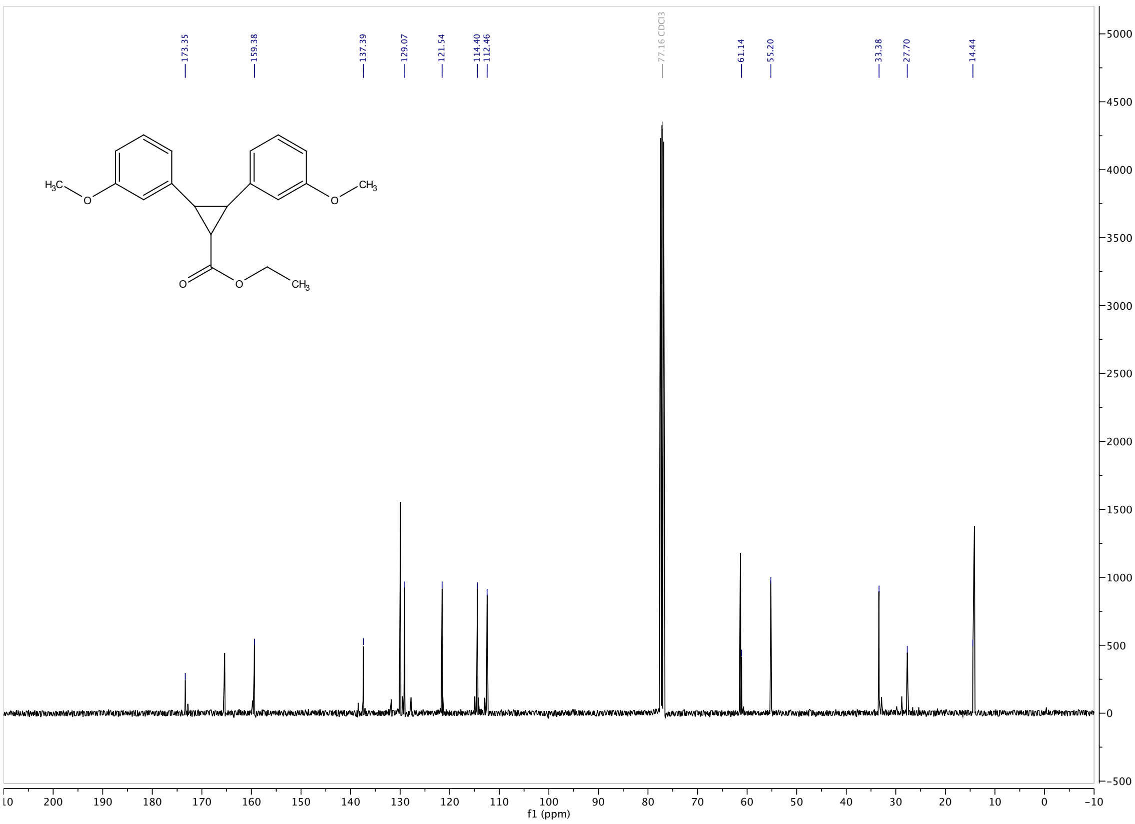

NMR-spectrum 4: ^{13}C-spectrum of **3**.

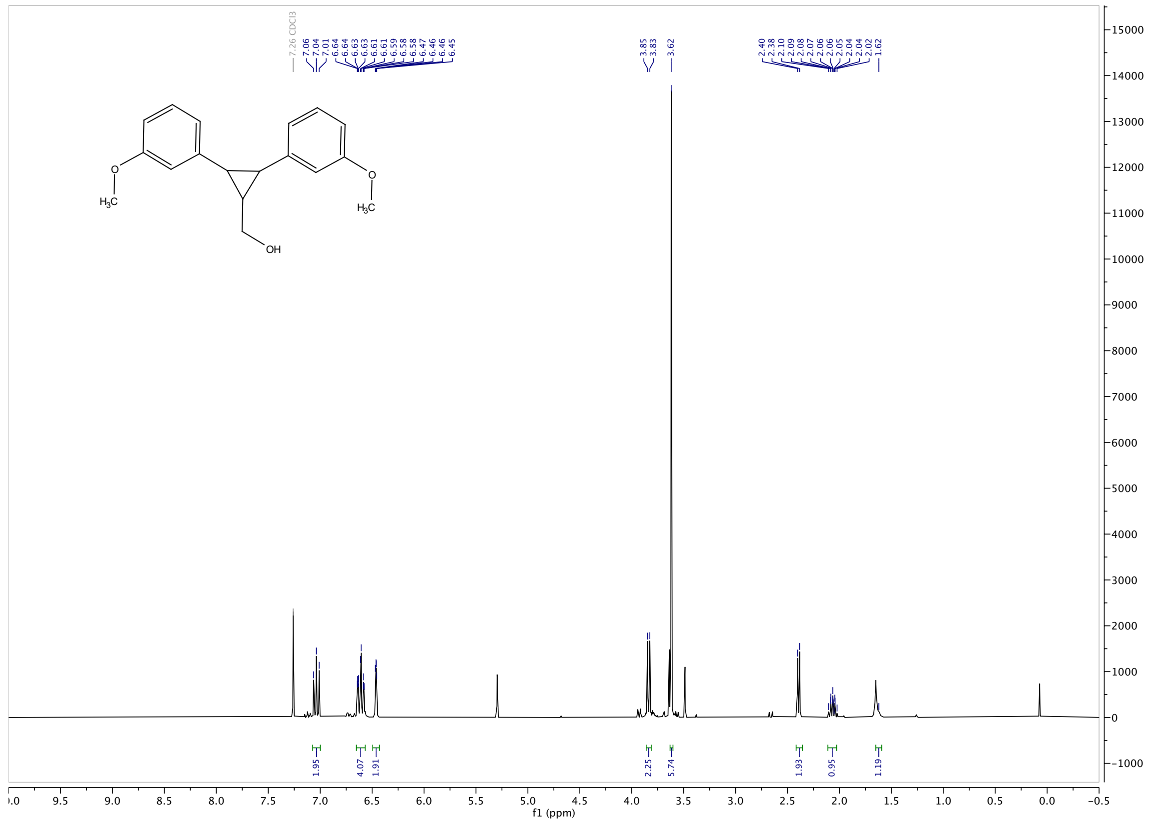

NMR-spectrum 5: ^{1}H-spectrum of **4**.

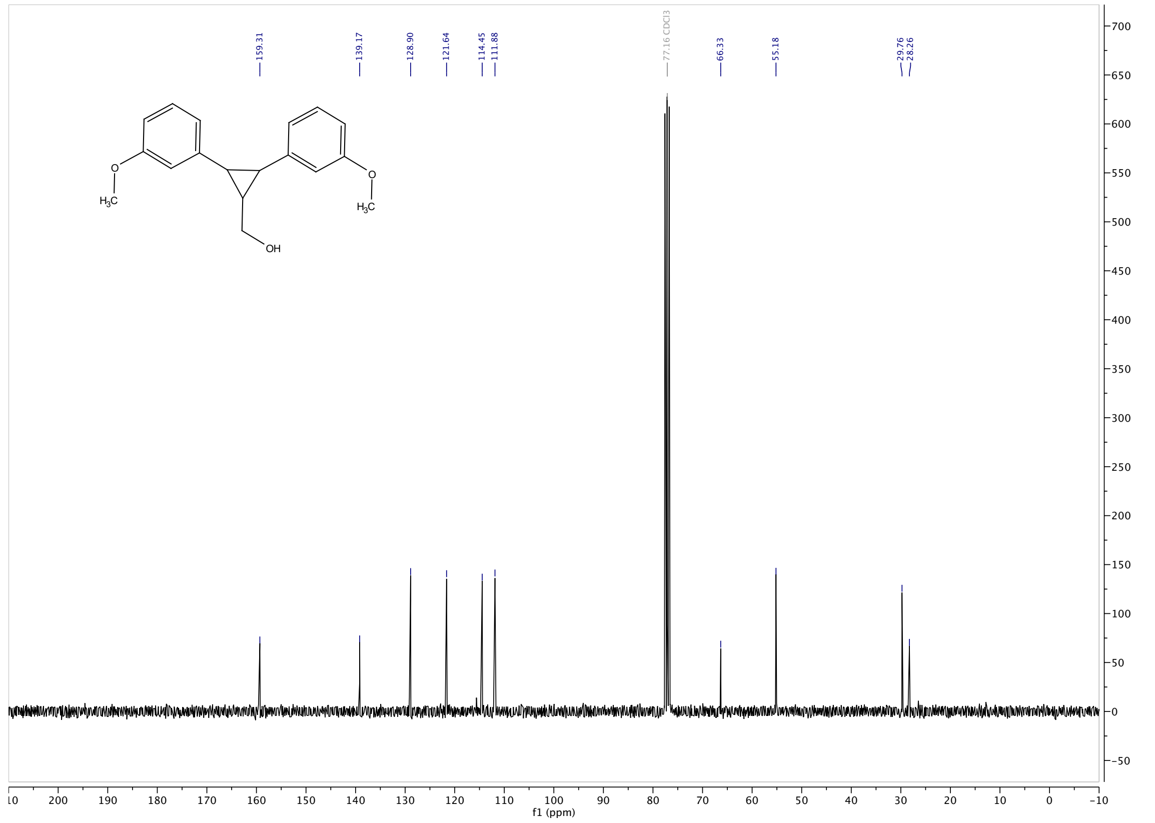

NMR-spectrum 6: ^{13}C-spectrum of **4**.

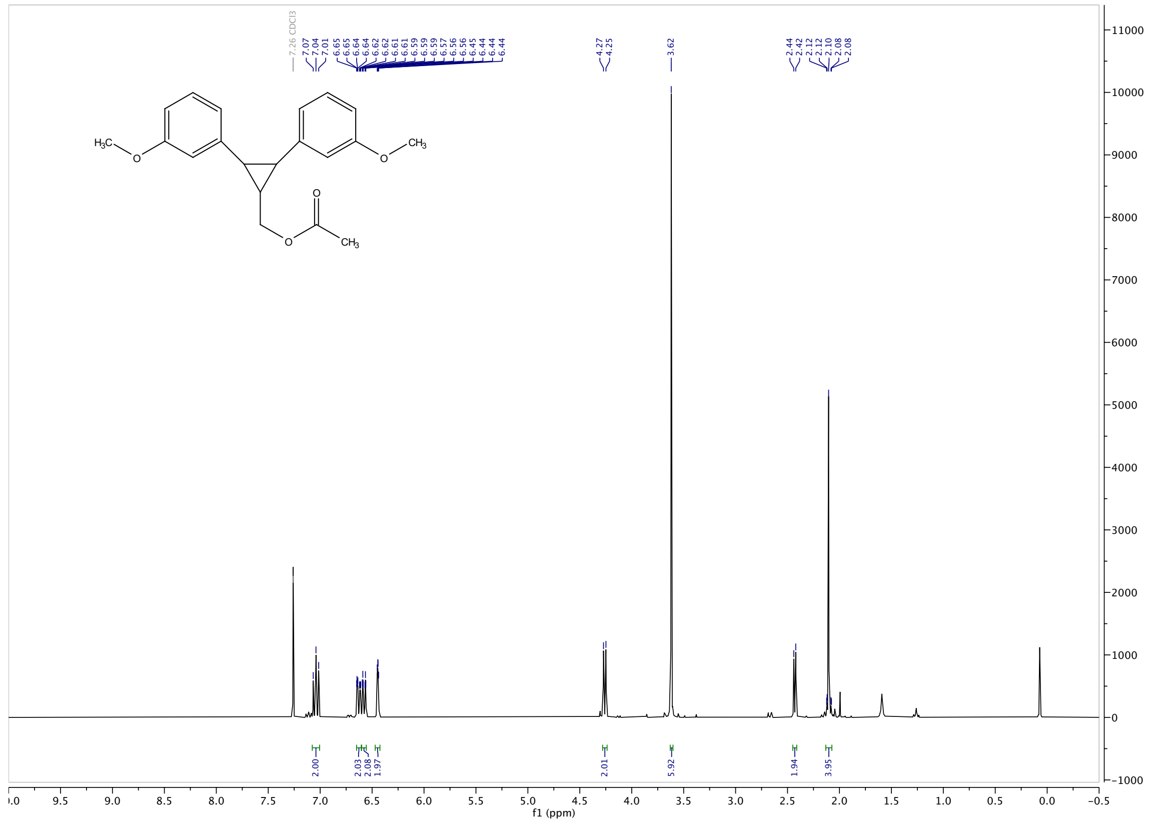

NMR-spectrum 7: ^{1}H-spectrum of **5**.

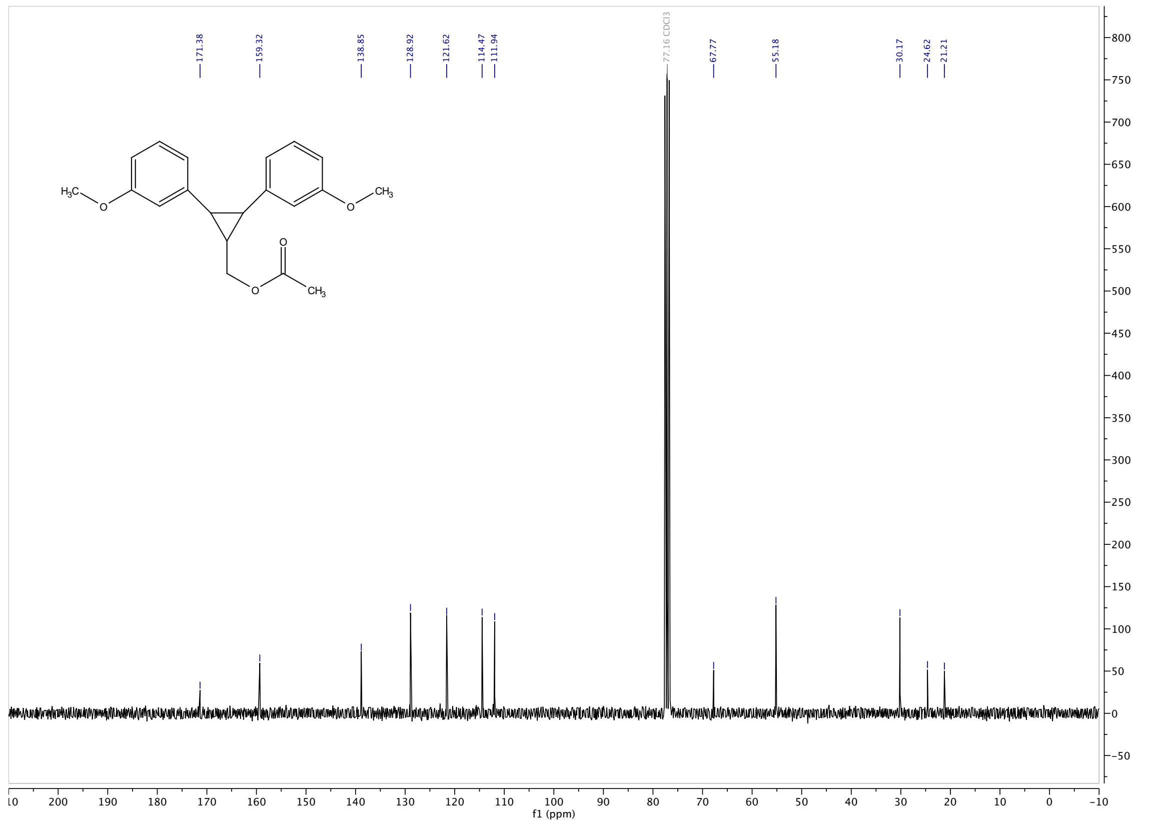

NMR-spectrum 8: ^{13}C-spectrum of **5**.

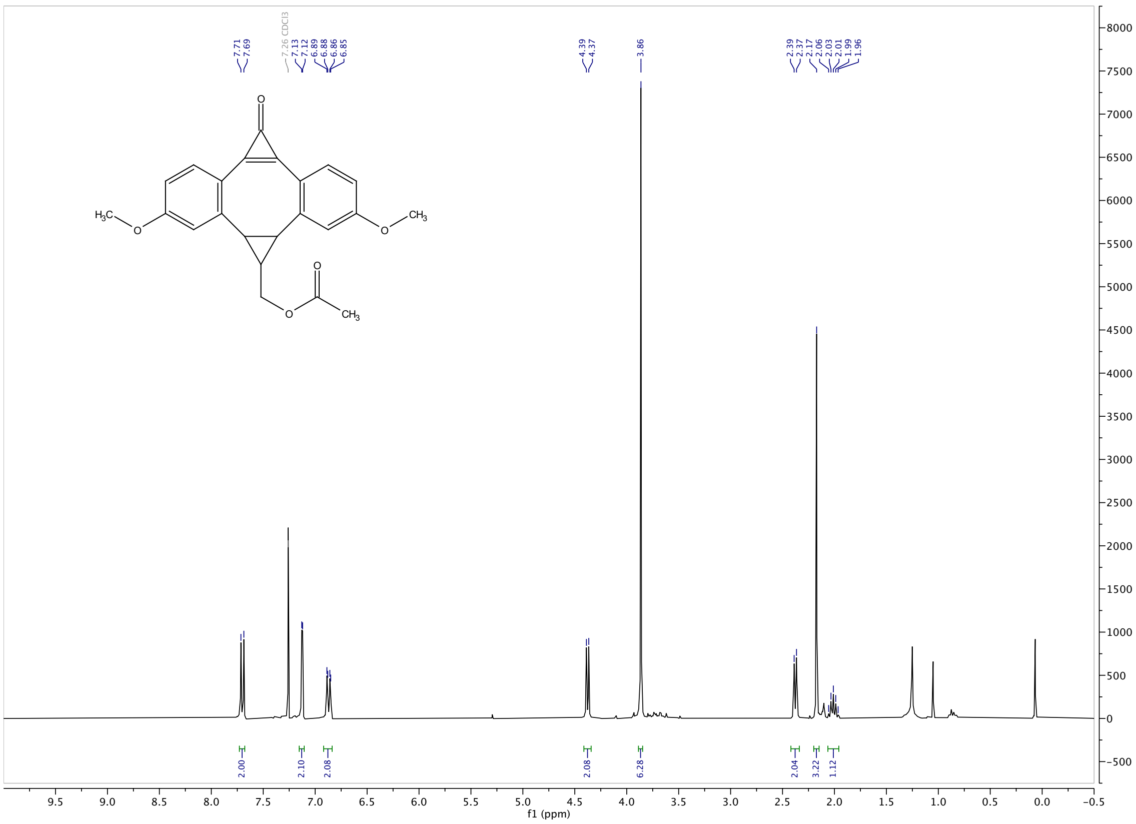

NMR-spectrum 9: ^{1}H-spectrum of **photo-DMBO**.

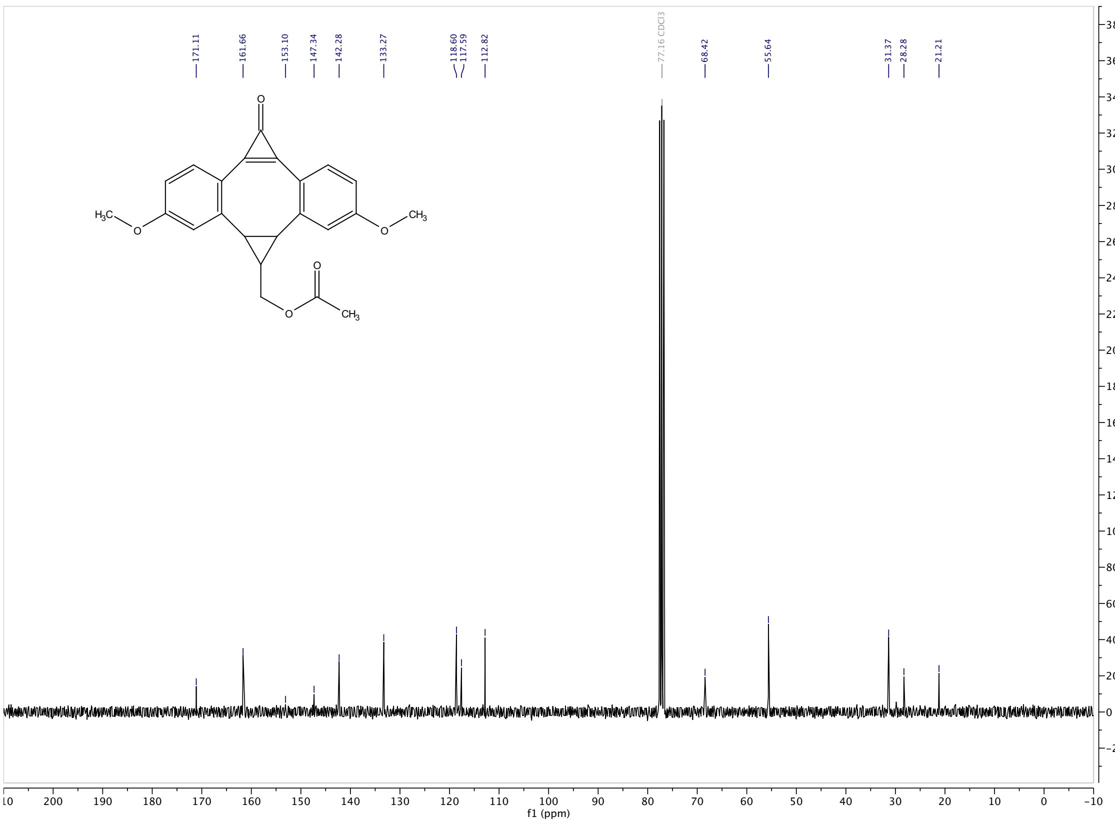

NMR-spectrum 10: ^{13}C-spectrum of **photo-DMBO**.

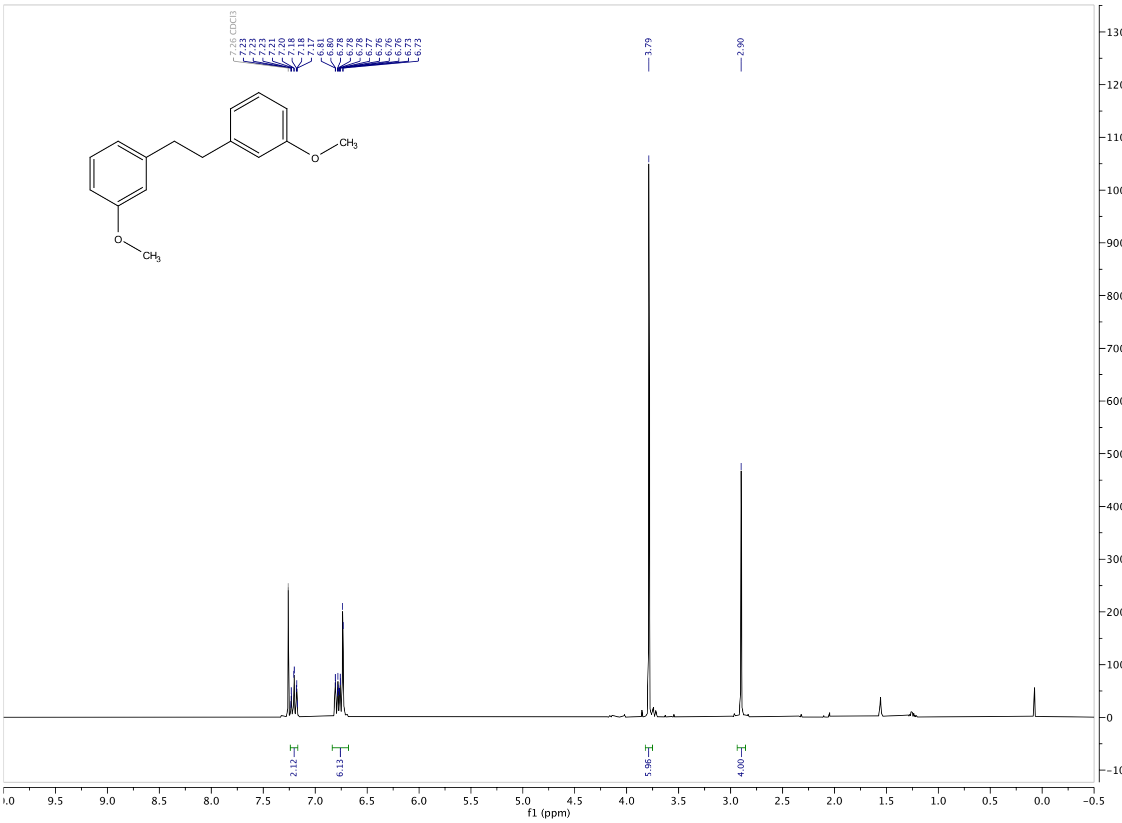

NMR-spectrum 11: ^{1}H-spectrum of **6**.

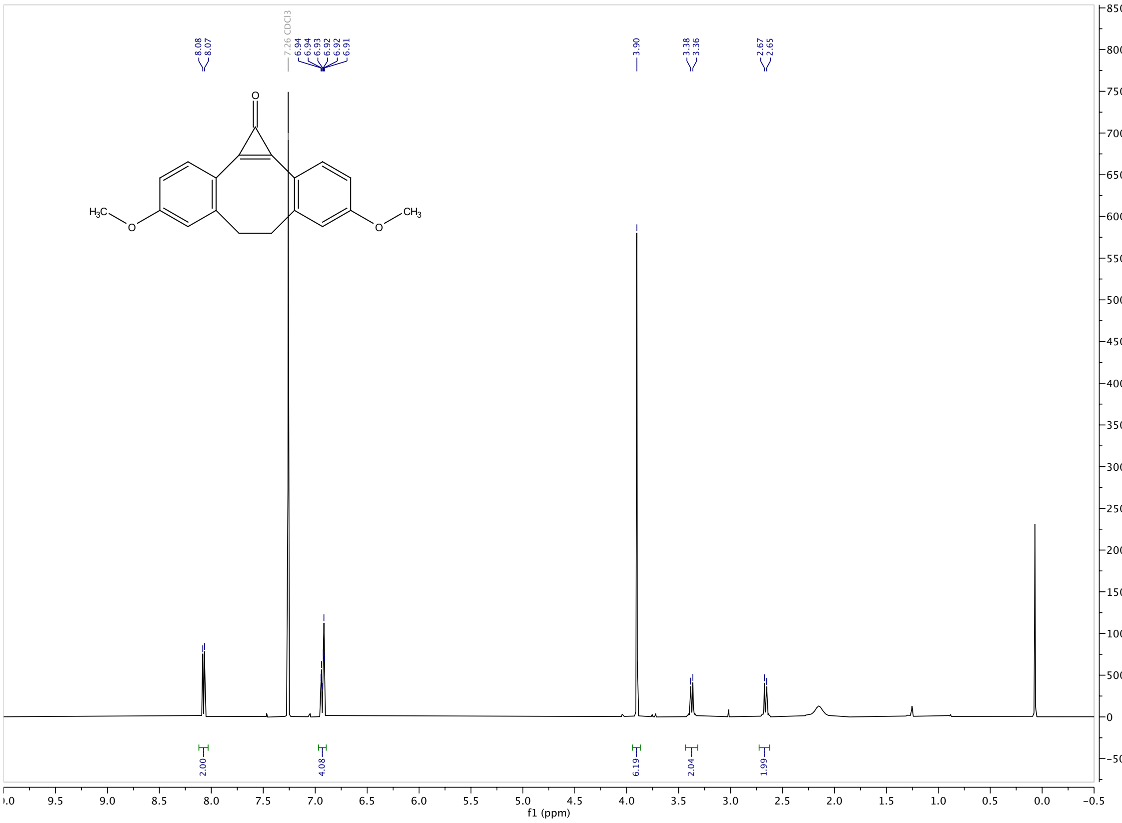

NMR-spectrum 12: ^{1}H-spectrum of **photo-DIBO**.

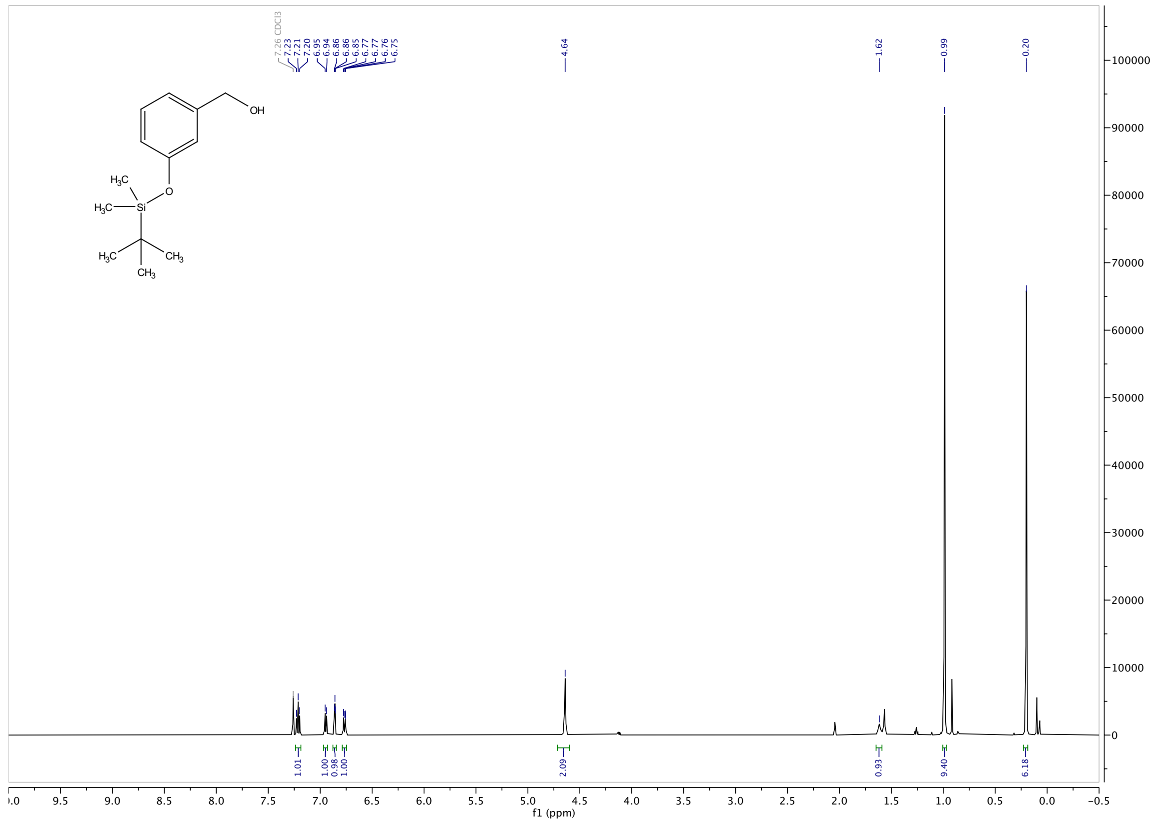

NMR-spectrum 13: ^{1}H-spectrum of **7**.

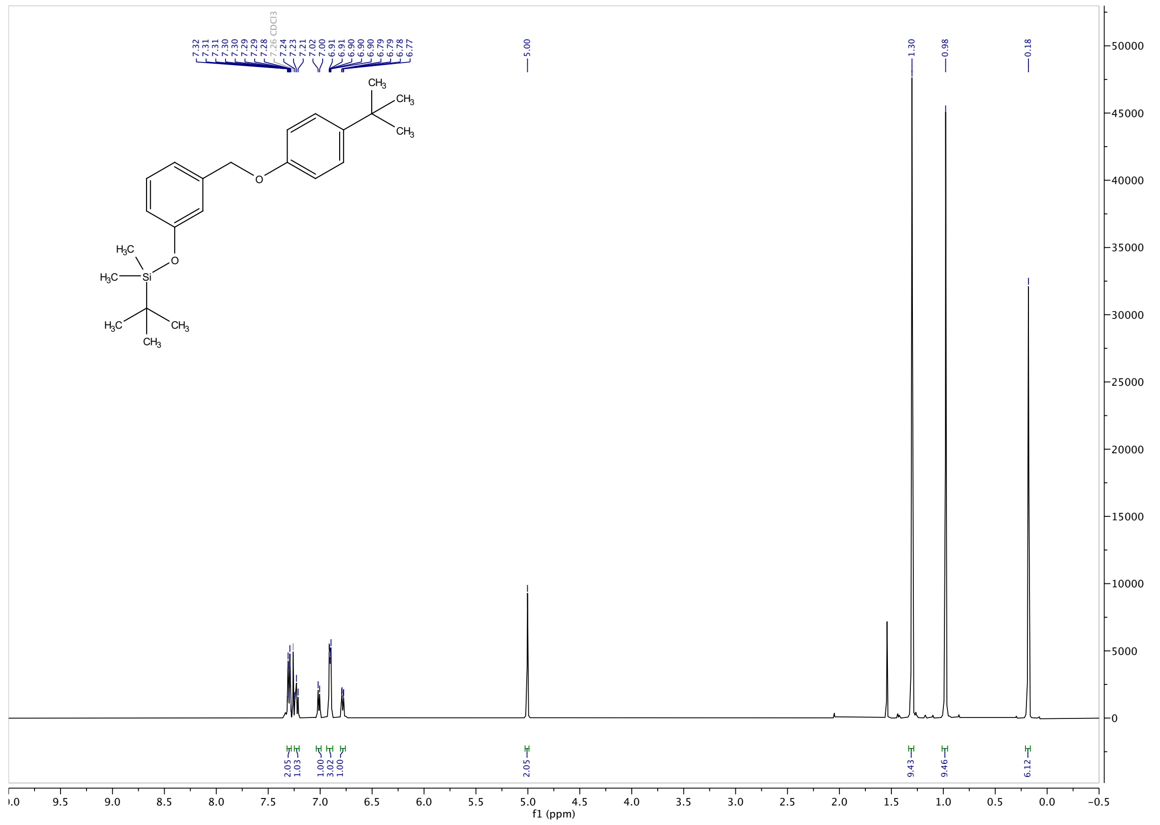

NMR-spectrum 14: ^{1}H-spectrum of **8**.

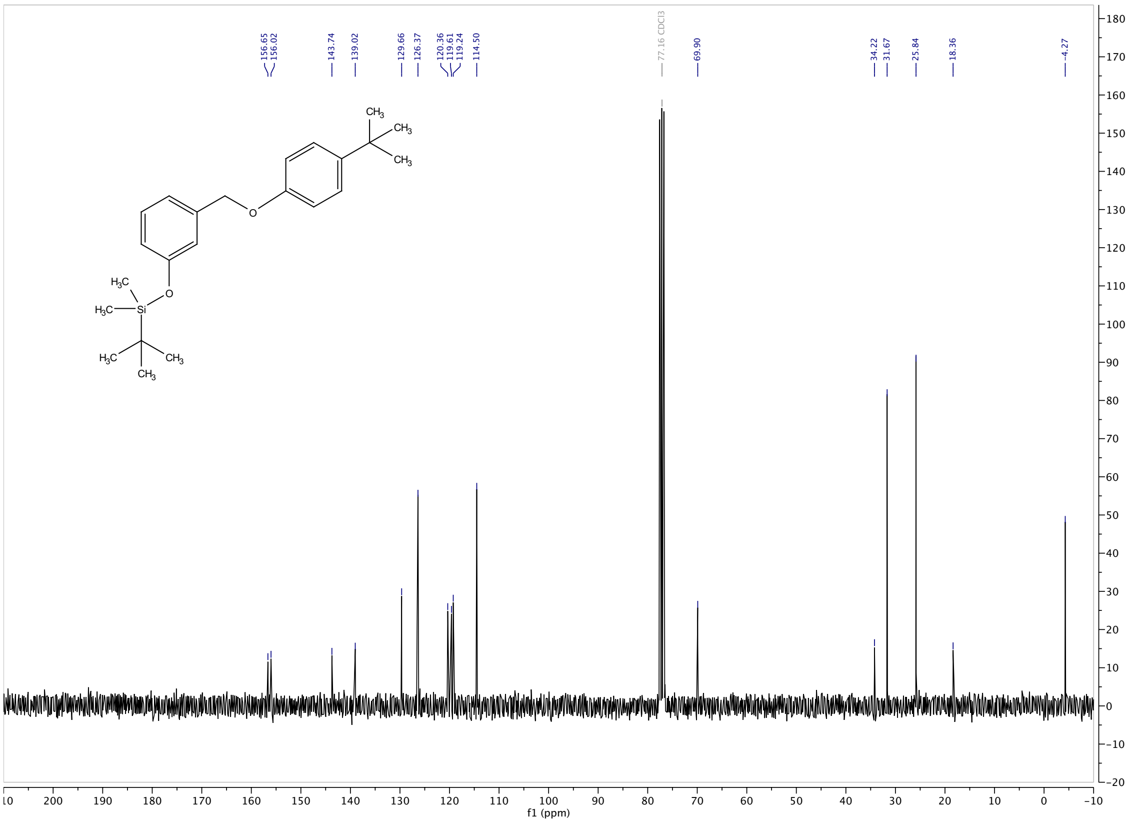

NMR-spectrum 15: ^{13}C-spectrum of **8**.

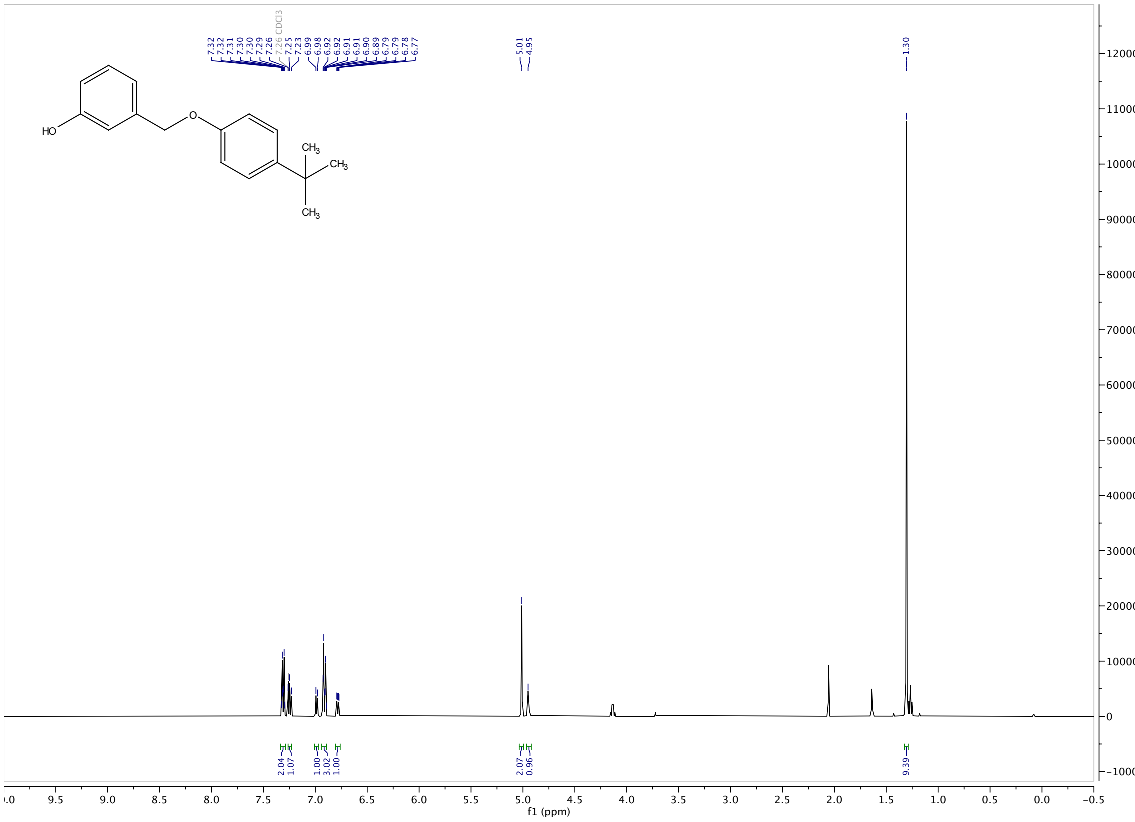

NMR-spectrum 16: ^{1}H-spectrum of **9**.

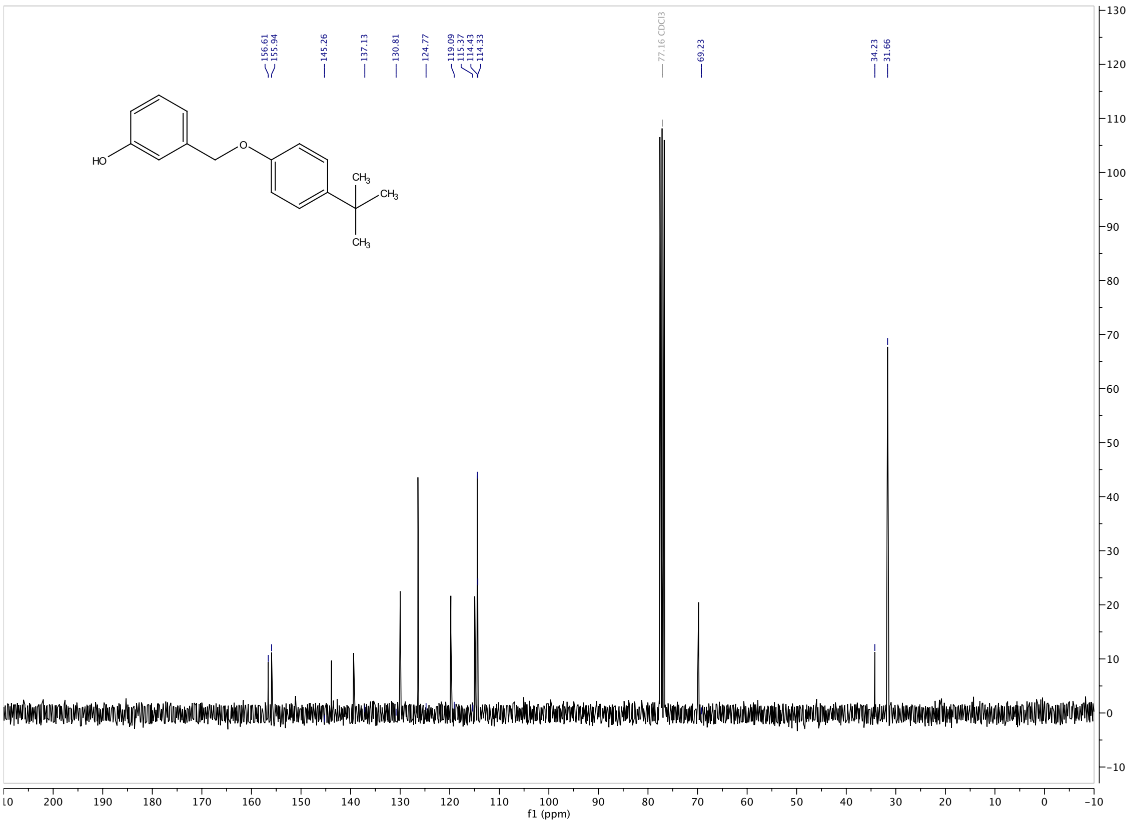

NMR-spectrum 17: ^{13}C-spectrum of **9**.

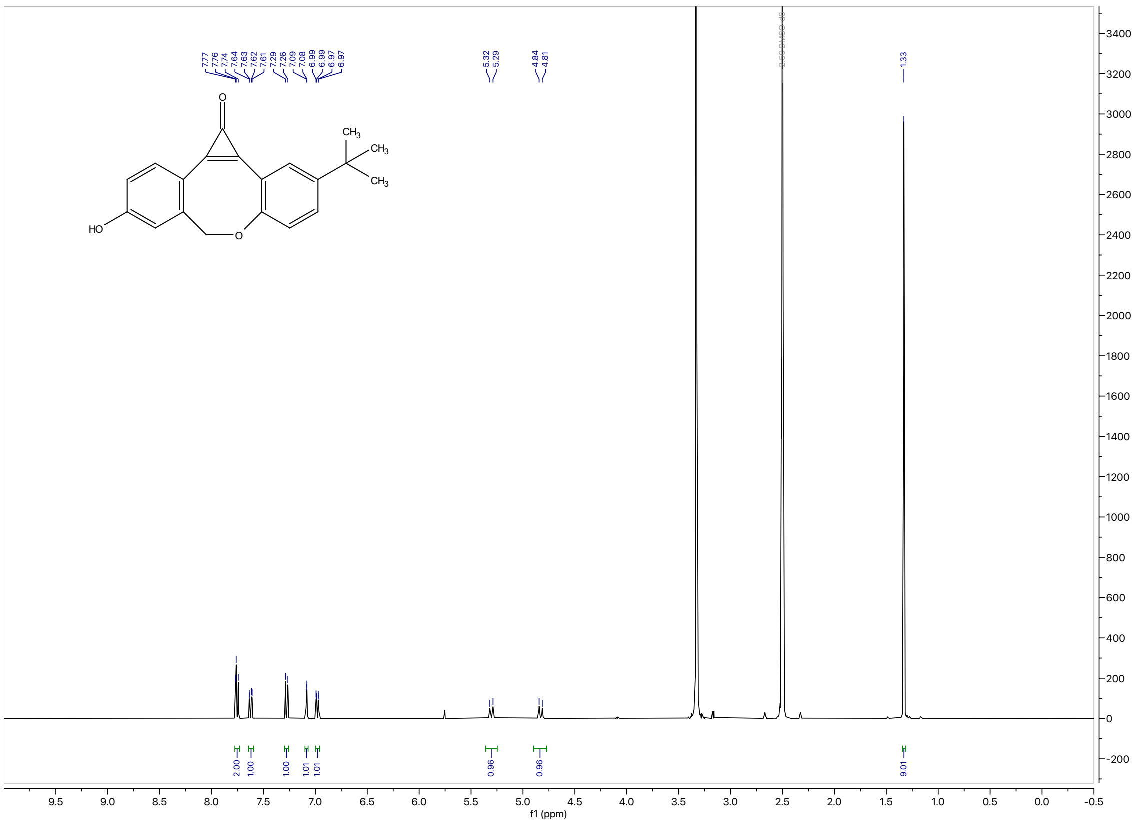

NMR-spectrum 18: ^{1}H-spectrum of **photo-ODIBO**.

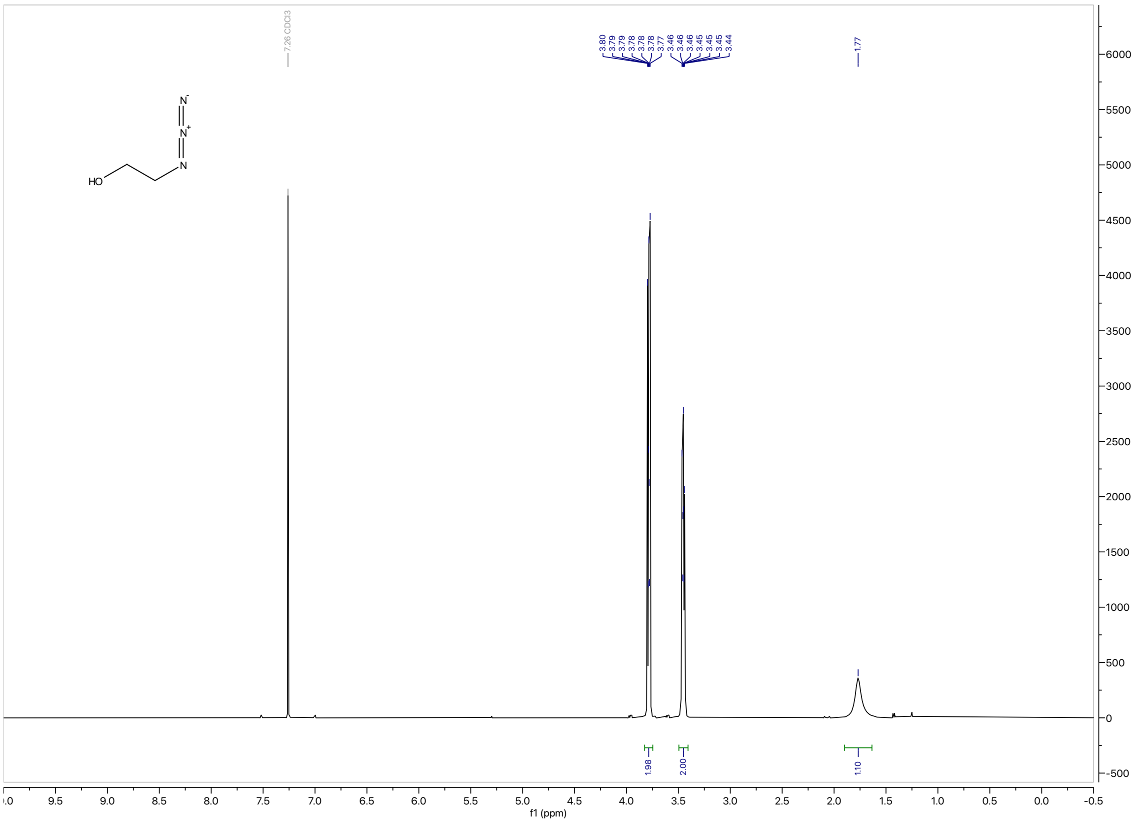

NMR-spectrum 19: 1H-spectrum of **pAz**.

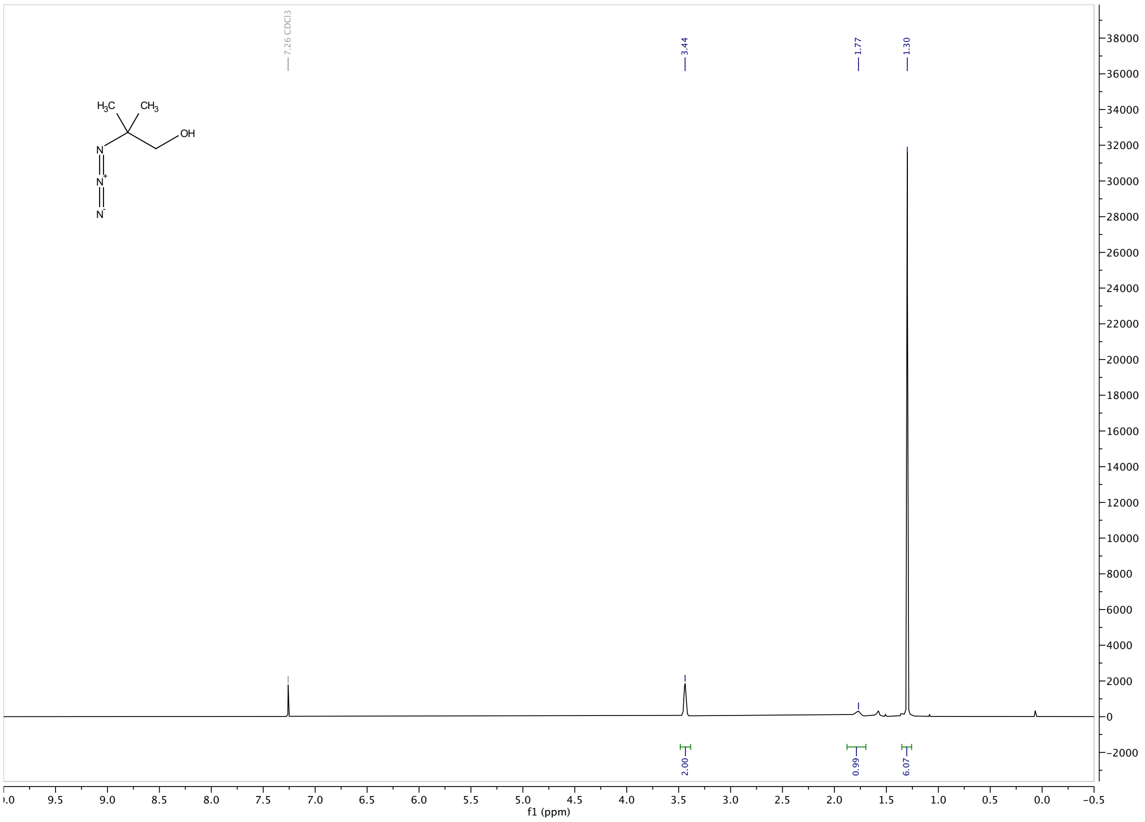

NMR-spectrum 20: 1H-spectrum of **tAz**.

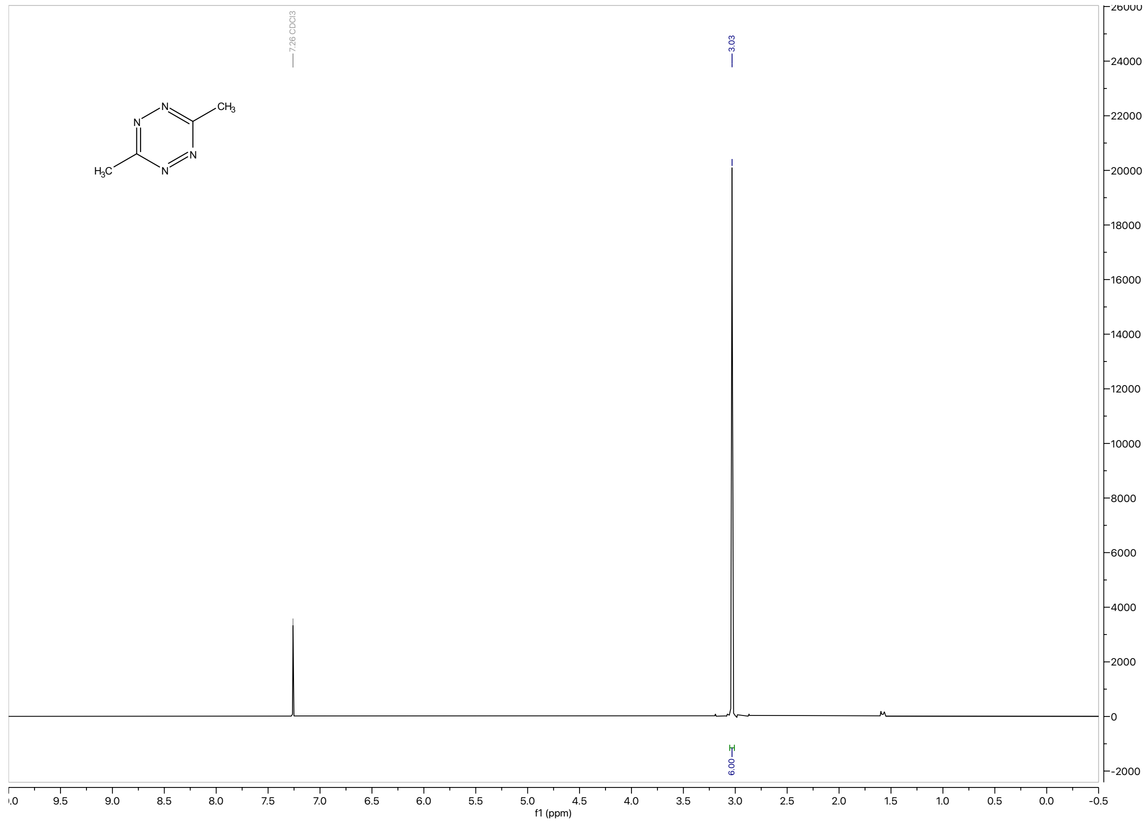

NMR-spectrum 21: ^{1}H-spectrum of **MeMe-Tet.**
